# Discovery of Sphingosine Kinase Inhibition by Modified Quinoline-5,8-Diones

**DOI:** 10.3390/ph18020268

**Published:** 2025-02-18

**Authors:** Ryan D. Kruschel, Kyle Malone, Alison N. Walsh, Christian Waeber, Florence O. McCarthy

**Affiliations:** 1 School of Chemistry and ABCRF, University College Cork, Western Road, T12K8AF Cork, Ireland; 111380606@umail.ucc.ie (R.D.K.);; 2 School of Pharmacy, University College Cork, Pharmacy Building, College Road, T12K8AF Cork, Ireland; kylem8414@gmail.com (K.M.); c.waeber@ucc.ie (C.W.); 3 Department of Pharmacology and Therapeutics, School of Medicine, University College Cork, T12XF62 Cork, Ireland

**Keywords:** sphingosine kinase, quinoline-5,8-dione, fragment-based design, kinase inhibition, cancer

## Abstract

**Background:** Sphingosine kinase (SphK) overexpression is observed in many cancers, including breast, renal and leukaemia, which leads to increased cellular proliferation, survival and growth. SphK inhibition has been an attractive target for anticancer drug development for the past decade, with SphK inhibitors such as PF-543 and opaganib exhibiting clinical antitumour effects. By exploiting both CB5468139 and PF-543 as structural leads, we hereby report on the first quinoline-5,8-dione-based SphK inhibitor using a fragment-based approach. **Methods:** The quinoline-5,8-dione framework was developed to incorporate two defined regions, namely a polar quinoline core, which links to an aryl lipophilic chain. All synthetic molecules were characterized by NMR and HRMS and assayed against SphK 1 and 2, and molecular docking studies were performed. A subset of compounds was screened for anticancer activity. **Results:** As the binding site of SphK accommodates the lipophilic tail of sphingosine, we initially set out to explore the substitution of the C(7) aryl moiety to attain eight novel C(7) ether-linked quinoline-5,8-diones, which were screened for SphK1 and SphK2 activity with good potency identified. To improve SphK binding, structural fragments were adapted from PF-543 to participate in hydrogen bonding within the binding site of SphK1. A model study was performed to yield novel compounds through activated C(2) formyl intermediates. Two pyrrolidine-based quinoline-5,8-diones were assayed for SphK activity, with **21** revealing an improvement of SphK1 binding efficacy relative to the parent compound and **20** (and its precursor **4**). Molecular modelling on the pyrrolidine quinoline-5,8-dione construct revealed favourable docking, low binding energies and opportunities for further improvement. **Conclusions:** Although the screening of anticancer activity was inconclusive, low micromolar dual SphK1/2 inhibition with the quinoline-5,8-dione framework has been identified for the first time, and a plausible new binding mode has been identified.

## 1. Introduction

Sphingosine kinase (SphK) upregulation is observed in many cancers, including breast, renal, prostate and leukaemia [1,2,3,4]. SphK catalyses the phosphorylation of ceramide-derived sphingosine to sphingosine-1-phosphate (S1P), a biologically active lipid mediator [5]. Two isoforms of SphK exist, SphK1 and SphK2, which catalyse the same chemical transformation but differ in their respective subcellular localization, biochemical properties and biological function [6]. S1P has an important role in regulating cell growth, survival and migration and has been implicated in diseases such as cancer, Alzheimer’s disease, pulmonary hypertension and multiple sclerosis [7,8,9,10]. In cancer cells, an increase in intracellular S1P is observed by an upregulation of SphK, triggering an increase in cellular growth, survival and migration. It is proposed that the inhibition of SphK would cease S1P production and increase pro-apoptotic ceramide production through a process known as the S1P/ceramide rheostat, a well-documented equilibrium that exists between the two lipids [11]. siRNA-mediated knockdown of SphK expression inhibits proliferation and increases ceramide levels to cause apoptosis of neuroblastoma tumour cell lines [12].

**Figure 1:**
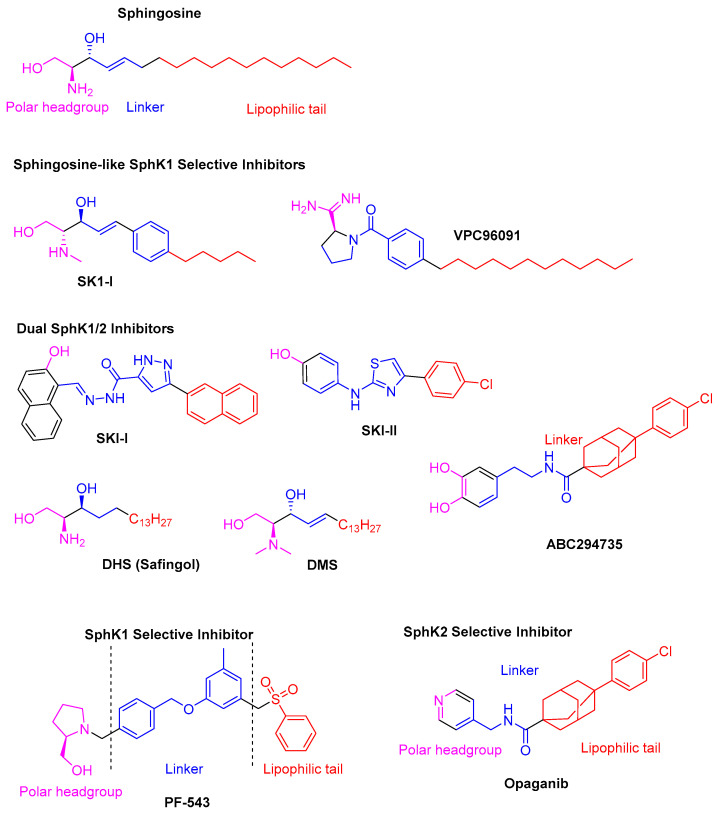
Sphingosine and representative examples of sphingosine kinase inhibitors. Representative fragments are coloured for the polar headgroup (pink), the linker (blue) and lipophilic tail (red) moieties.

Early drug development targeted SphK1 for oncology purposes (Figure 1); however, further studies have shown that both isoforms are involved in regulating cancer cell processes [2,13,14,15,16]. PF-543 is the most potent SphK1-specific inhibitor reported to date (Ki = 3.6 nM) (Figure 1) [1]. Like many potent SphK inhibitors, its structure is based on endogenous sphingosine, namely a polar headgroup, linker and lipophilic tail [11]. This is clearly seen in Figure 1, which highlights the polar headgroup, linking group and lipophilic tail common to the majority of SphK inhibitors.

The selective SphK1 inhibitor PF-543 exhibits cytotoxic effects on both colorectal cancer cells and head and neck squamous cell carcinoma (SCC) [17]. Colorectal cancer cell lines HCT-116, HT-29 and DLD-1 all exhibited necrosis once treated with PF-543 (10 μM), albeit when used at 2.5–10-fold higher concentrations than required for S1P reduction in cells. Interestingly, the HT-29 colorectal cancer cell line, which expresses the highest SphK1 levels, was more vulnerable to PF-543 treatment than DLD-1, which has the lowest SphK1 expression. Long-term in vivo studies on HCT-116 tumour-presenting mice administered PF-543 resulted in tumour shrinkage and longer survival with no apparent toxicity observed [18]. SCC cell treatment with PF-543 (25 μM) decreased the proportion of viable cells in a time- and dose-dependent manner. PF-543 is thought to induce cell autophagy, apoptosis and necrosis, with evidence of all three confirmed using flow cytometry. Interestingly, the addition of the ROS scavenger *N*-acetyl-L-cysteine reduced the cytotoxic effects of PF-543 on SCC cells, with necrotic SCC cells induced by PF-543 decreased from 48.6% to 26.3% [19].

SphK2 is also a well-established anticancer target, with the SphK2 inhibitor opaganib (ABC294640, K_i_ = 9.8 μM) successfully reaching phase 2 clinical trials for prostate cancer (Figure 1) [20,21,22,23,24]. Though no crystal structure currently exists of SphK2, opaganib acts as a competitive inhibitor relative to sphingosine and is predicted to bind in the lipid-binding site [21]. Interestingly, the related compound ABC294735, incorporating a change in the *N*-substituent, is identified as a dual SphK1/2 inhibitor [22].

In the analysis of SphK inhibitors, the majority conform structurally to sphingosine, with a high degree of lipophilicity and flexibility to aid in binding in the narrow lipid-binding pocket of SphK [1,25]. Recent studies with a number of isoform-selective SphK1 and SphK2 inhibitors have not clinically outperformed PF-543 and opaganib, so there is merit in further exploration of the model [22]. In identifying SphK inhibitors with clinical benefit, the dual SphK1/2 inhibitor safingol has been effective in combination with cisplatin in Phase 1 clinical trials against glioma [26]. In addition, another dual SphK1/2 inhibitor, SKI-II, has been identified to be effective in combination with temolozomide at sublethal doses in glioblastoma cell lines [27].

**Figure 2:**
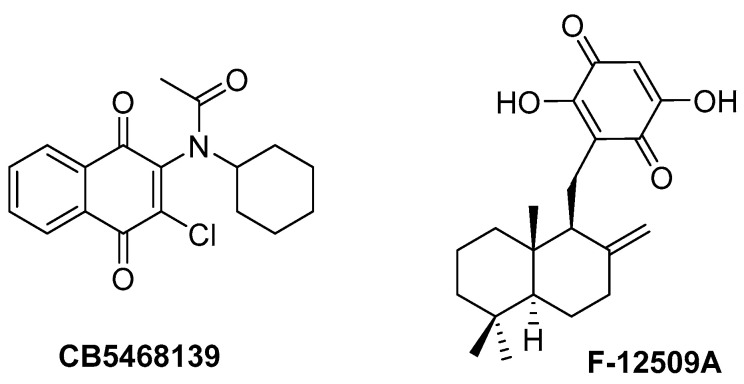
Quinone-based SphK inhibitors CB5468139 and F12509A.

Looking at other reported SphK inhibitors, there are a number of compounds that incorporate a quinone. The quinone-based SphK inhibitor CB5468139 was first identified in 2012 by screening the Chembridge Diverset collection for SphK1 inhibitors (Figure 2). CB5468139 exhibited high potency towards SphK1 (Ki = 0.3 µM) without affecting SphK2 activity up to 100 µM [28]. Moderate cytotoxicity is reported with the reduced proliferation of A498 kidney adenocarcinoma cells (EC_50_ = 10–15 μM). However, since CB5468139 has been reported to inhibit multiple kinases, including Met, MST2 and CLK1 (suggesting it is not selective for SphK1), it is difficult to define whether its effect on cells is due to on or off SphK target interactions [1]. Another quinone, F-12509A, was isolated from the fungus *Trichopezizella barbata* and identified very early as an SphK inhibitor and fits the polar/linker/lipophilic framework with the potential for covalent/irreversible activity [29].

**Figure 3:**
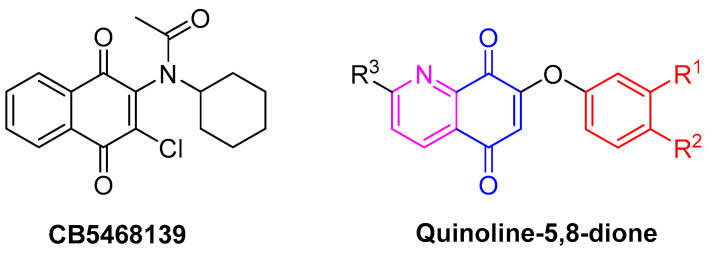
Naphthoquinone CB5468139 and our quinoline-5,8-dione SphK inhibitor framework.

Our approach to developing new inhibitors of SphK sees quinoline-5,8-dione as a key fragment analogous to the naphthoquinone-based CB5468139. It is a known pharmacophore in NAD(P)H:quinone oxidoreductase 1 (NQO1) inhibition capable of redox cycling in vivo and can result in the oxidation of critical amino acids in targeted enzymes [30,31,32]. Our initial premise is to exploit the structural relationship between naphthoquinone-based CB5468139 and quinoline-5,8-dione (Figure 3) to generate a polar pyridine and quinone central quinoline-5,8-dione core and an ether-connected lipophilic tail. We set out to install lipophilic aryl moieties to fill the space of the cyclohexane ring and to probe the lipophilic binding site of SphK. The activity of novel quinoline-5,8-diones was screened for SphK inhibition, followed by anticancer activity to test the viability of the framework as an anticancer agent. We hereby report the development of a new framework for SphK inhibition.

## 2. Results and Discussion

### 2.1. Development of the Quinoline-5,8-Dione Intermediate

**Scheme 1:**
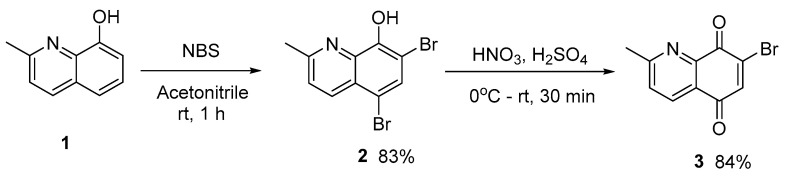
Route to versatile quinoline-5,8-dione framework **3**.

Our initial premise exploits the structural relationship between naphthoquinone-based CB5468139 and quinoline-5,8-dione (Figure 3). It was proposed that the replacement of the tertiary amine of CB5468139 would allow the exploration of new chemical space with an ether linker to a lipophilic tail analogous to that of PF-543. The synthetic approach adopted utilised a two-step route to successfully furnish 7-bromo-quinoline-5,8-dione **3** in 70% overall yield (Scheme 1) [33]. This approach is favoured as the quinone moiety is installed from the outset, removing the need to oxidise post-substitution (which, on scoping the transformation, proved problematic). Literature precedence suggests that the presence of the C(7) bromine can be exploited to guide the chemical transformation [34,35,36,37]. In addition, the presence of the 2-methyl group can be probed as a chemical handle.

### 2.2. Synthetic Route to 7-Phenoxy Quinoline-5,8-Diones

**Scheme 2:**
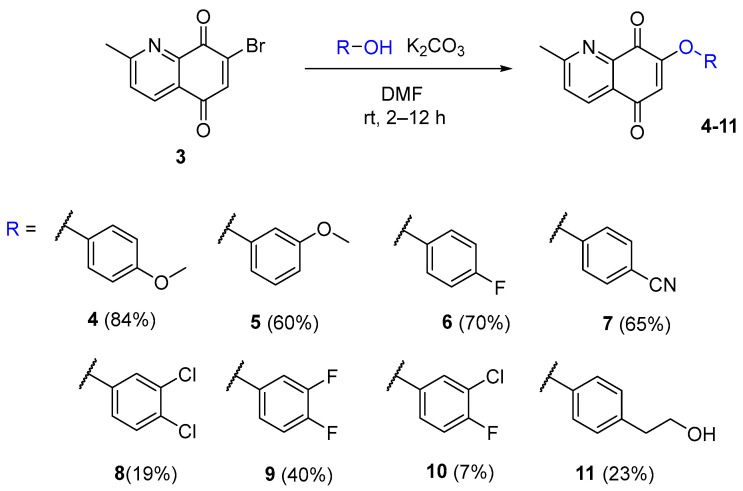
C(7) substitution to furnish the phenoxy ether products.

The synthesis of the target framework commences from 7-bromo-quinoline-5,8-dione **3** (Scheme 1) [32]. The lipophilic tail can be installed in the core by employing a robust and facile chemoselective displacement of the C(7) bromine using a range of phenols (Scheme 2). The incorporation of substrate lipophilicity is well understood to increase molecular interactions, and our primary screen incorporated phenols, including halogen substitution, to probe the lipid-binding site of SphK [38,39,40]. The displacement of bromide with the requisite phenoxide led to the formation of nine novel quinoline-5,8-dione analogues [36]. This reaction proceeded under ambient conditions with variable yields (7–84%). It is noteworthy that a *meta*-chloro substituent on the phenol moiety resulted in a reduced yield of **8** (19%) and **10** (7%).

### 2.3. Screening of Quinoline-5,8-Diones for Sphingosine Kinase Inhibition

**Figure 4:**
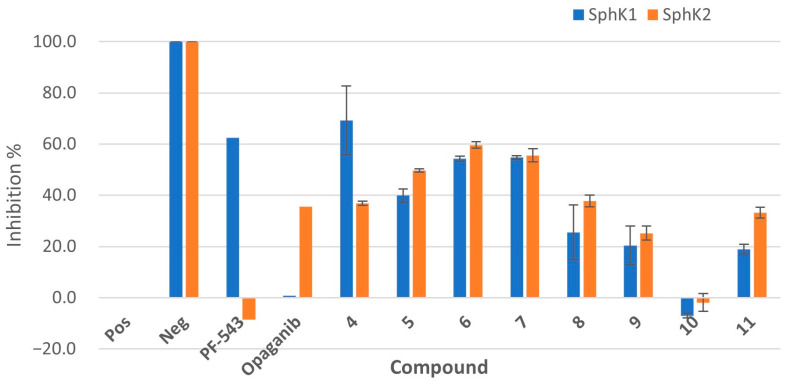
SphK assay with representative subset of 7-phenoxy quinoline-5,8-diones. Measurement of fluorescence intensity with excitation at 485 nm and emission at 535 nm reported as a percentage of negative control. Novel quinoline-5,8-diones and opaganib were incubated at 10 μM conc., PF-543 = 100 nM conc.

Preliminary sphingosine kinase inhibition screening was performed on eight analogues (**4**–**11**), incorporating PF-543 and opaganib as reference SphK1 and SphK2 inhibitors, respectively. The fluorescence-based assay involved incubating the quinoline-5,8-diones with SphK isoforms individually (recombinant SphK1 and SphK2 in duplicate) [41]. The inhibitory activity (percentage of positive control) of the compounds against both SphK isoforms is illustrated in Figure 4 (see fluorescence data in Supplementary Materials, Tables S2–S5). With respect to the inhibitory references, PF-543 was tested at a 100 nM concentration (within its reported inhibitory range) and, as expected, exhibited high SphK1 selectivity with 63% inhibition; Opaganib at 10 µM exhibited high SphK2 selectivity and 36% inhibition (reference Ki = 9.8 μM) [19,22]. The investigational quinoline-5,8-diones were tested at a 10 μM concentration, with all but one of our initial panels exhibiting the inhibition of SphK1 and SphK2 with as high as 69% inhibition at the 10 μM concentration. The majority of compounds yielded dual effects on SphK1 and SphK2, with compounds **5**–**7** and **9** equipotent against both isoforms. The *para*-F **6** and *para*-CN **7** were the best dual inhibitors, both exhibiting ≥ 54% inhibition against SphK1 and ≥ 56% against SphK2. The *para*-methoxy derivative **4** exhibited a level of selectivity for SphK1 (and compounds **8** and **11** against SphK2, albeit at lower potency). Increasing the lipophilicity via the addition of two halogens to the aryl moiety to yield **8**, **9** and **10** surprisingly resulted in a reduction in SphK1 inhibition (26%, 20% and 0%, respectively), so increasing lipophilicity does not improve binding. Highlighting this, *para*-F and *meta*-Cl **10** exhibited 0% inhibition against both isoforms. *Para*-hydroxyethyl **11** is identified to have good potency and signals that there is room for substitution and, indeed, H-bonding groups in the binding orientation of this compound class. There does not appear to be a linear relationship between the electronic effects of Cl, F, OMe and CN and binding. However, the performance of compounds **4**–**8** signalled further potential, and we set out to probe whether improved potency and selectivity could be achieved.

### 2.4. Extension of the Quinoline-5,8-Dione Framework to Target SphK1 Inhibition

**Figure 5:**
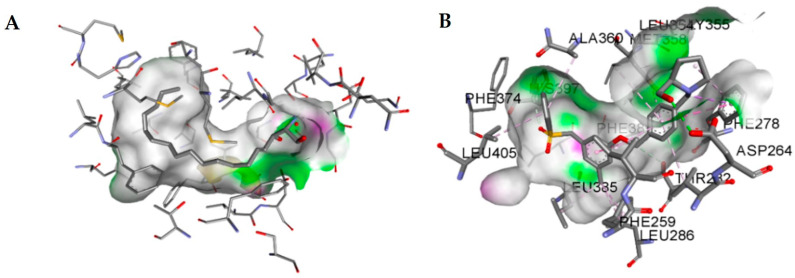
(**A**) Visualisation of sphingosine in lipid-binding site of SphK1 (PDB 3VZB); (**B**) PF-543 in ligand-binding site of SphK1. Areas of green represent H-bonding accepting sites, while areas of purple represent H-bonding donor sites (PDB 4V24) [48].

Encouraged by the identification of SphK inhibition using an entirely novel compound library, a modelling approach was adopted to improve SphK1 inhibition of the quinoline-5,8-dione framework. Kim et al. reported that alteration to the linker aromatic region of PF-543 could result in more cytotoxic analogues, and, indeed, many others have identified chemical space within the binding pocket [42,43,44,45,46,47]. Similar to sphingosine (Figure 5A), the flexibility of PF-543 allows it to feed down the narrow ‘J-channel’ of SphK1, as illustrated in Figure 5B. SphK1 selectivity of PF543 originates from the structural difference in the ligand-binding site between SphK1 and SphK2. The lipid-binding sites of SphK1 and SphK2 differ by only three amino acid residues at positions 374, 260 and 358; SphK1 has Phe, Ile and Met and SphK2 has Cys, Val and Leu in the respective positions [48,49]. The structural differences result in a larger binding site in SphK2 [50].

**Scheme 3:**
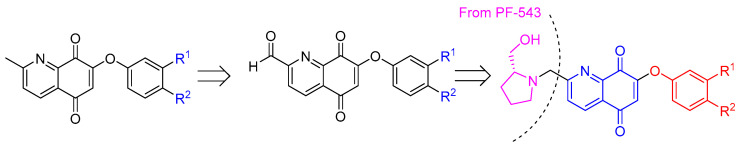
Incorporating the pyrrolidine headgroup into the quinoline-5,8-dione.

In addition to the multiple hydrophobic interactions observed throughout the ‘J-channel’ of SphK1, one key formal hydrogen bond interaction is observed between Asp264 and the pyrrolidine hydroxyl group [48]. Thus, we undertook the incorporation of a D-prolinol fragment at the 2-position of quinoline to probe the effect on SphK1 binding (Scheme 3). This has the effect of extending the polar headgroup from the pyridine (as in opaganib) towards that of an SphK1 selective inhibitor, PF-543. The pyrrolidone headgroup offers known hydrogen-bonding interactions within the lipid-binding site of SphK1, and thus, a synthetic route was designed in the quinoline-5,8-dione framework to improve SphK1 inhibition potency [1,49].

### 2.5. Synthesis of the Quinoline-5,8-Dione Framework to Target SphK1 Inhibition

**Scheme 4:**
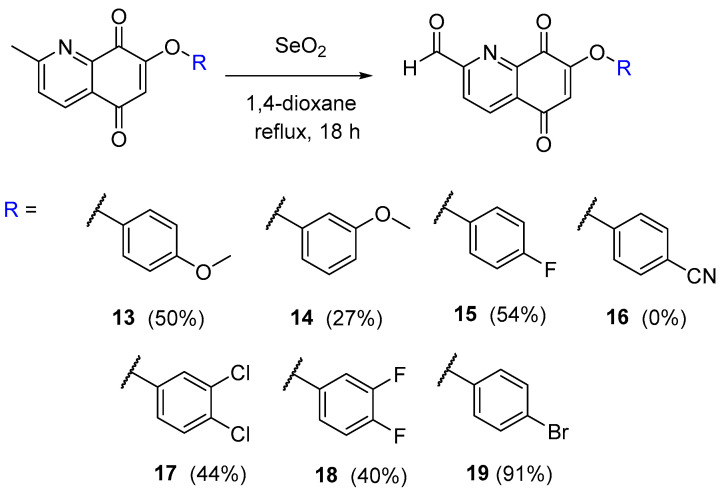
Selenium dioxide oxidation to functionalise the C(2) position.

The C(2) methyl group offers a useful chemical handle, employing selenium dioxide as an oxidant. A similar oxidative approach is utilised to functionalise the unreactive C(2) position of methyl quinoline-5,8-dione precursors to the anticancer agent Lavendamycin [51,52,53]. A selection of seven phenyl ether quinoline-5,8-diones from Scheme 2 was chosen (**4**–**9**), and, in addition, the 4-bromo analogue **12** was included (**12** was prepared as in Scheme 2 in 66%). The oxidation of the C(2) position proceeded with variable isolated yields (27–91%, Scheme 4).

**Figure 6:**
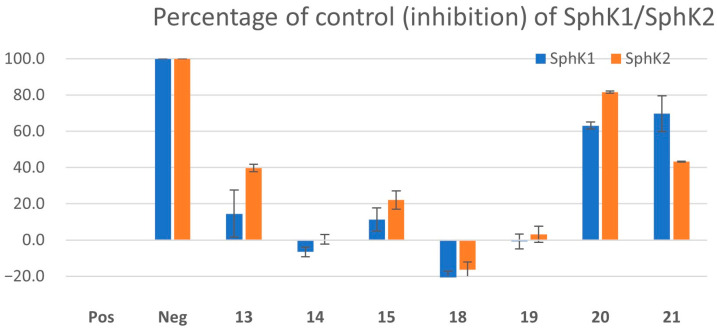
SphK inhibitory assay with representative subset of 2-formyl and 2-pyrrolidinyl quinoline-5,8-diones. (2-substituted quinoline-5,8-diones incubated at 10 μM conc.).

The oxidation of nitrile **7** was unsuccessful after multiple attempts (the aldehyde product **16** was identified on NMR analysis, but the complex product mixture was inseparable using chromatographic techniques). Five of the aldehyde products (**13**–**15**, **18**–**19**) were chosen for screening against sphingosine kinases, and there was a noticeable drop in inhibition, with activity only evident in the *para*-methoxy **13** and *para*-fluoro **15** derivatives (Figure 6). Subsequent efforts progressed with these two aldehydes for reductive amination to form the pyrrolidine.

**Scheme 5:**
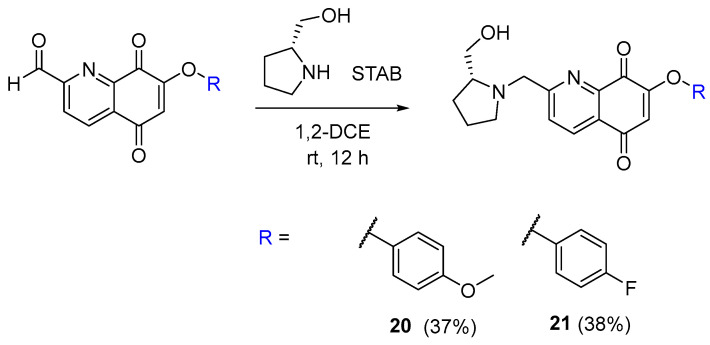
Reductive amination to attach the pyrrolidine headgroup.

D-Prolinol was reacted with the formyl group of **13** and **15** through a reductive amination to furnish the pyrrolidine headgroup [54]. The products **20** and **21** were isolated as colourless oils in moderate yields (37–38%) (Scheme 5).

Both **20** and **21** are directly related to the most potent SphK inhibitors identified from preliminary screening (**4** and **6**) and were screened for SphK inhibitory activity (Figure 6). *Para*-Fluoro **21** exhibited an improvement in SphK1 inhibition potency (70%) with a slight reduction in SphK2 inhibition potency (43%) relative to the parent compound **6** (54% and 60%, respectively). Conversely, **20** exhibits better inhibition of SphK2 (82% vs. 37% for precursor **4**) and slightly worse for SphK1 (63% vs. 69% for **4**).

**Figure 7:**
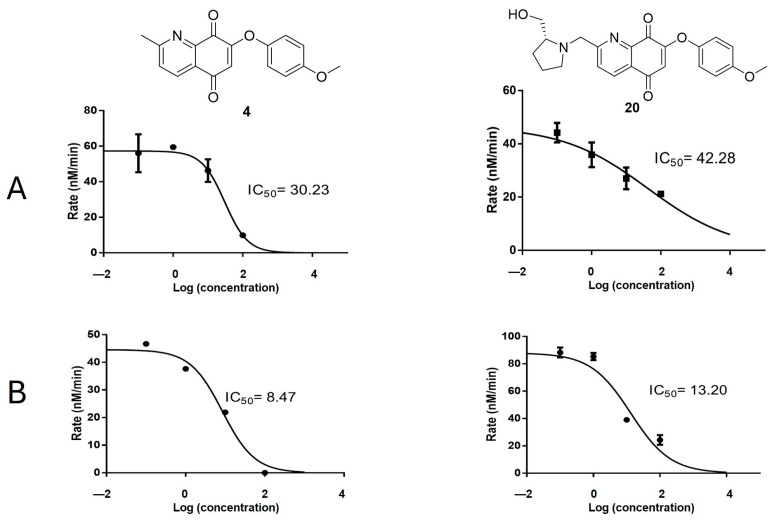
SphK inhibition assay with two lead quinoline-5,8-diones **4** and **20**. (**A**) = SphK1 inhibition; (**B**) = SphK2 inhibition (IC_50_ values in µM).

In order to confirm the activity against SphKs, we next conducted an IC_50_ assay against SphK1 and SphK2. Two related compounds were chosen for this, **4** and **20**, which both possess the *para*-OMe phenol and differ at the 2-position, with **4** containing the lipophilic methyl and **20** the hydrophilic pyrrolidine (Figure 7). Compound **4** is identified with an IC_50_ of 30 and 8 µM against Sphk1 and SphK2, respectively (and **20** with values of 42 and 13 µM, respectively). It is interesting to note that upon incorporation of the pyrrolidine, no significant benefit is identified over the starting methyl substituent.

In summary, synthesis of a new panel of 2- and 7-substituted quinoline-5,8-diones is reported for the first time, which exhibits dual sphingosine kinase inhibition at clinically relevant concentrations.

### 2.6. Molecular Model of the Quinoline-5,8-Dione Framework in SphK1

Given the promising results, docking simulations for the SphK1 model were performed to identify predicted binding poses for SphK1 inhibitors in the series. Docking simulations were calculated using the CLC Drug Discovery Workbench 3.0, which utilises the docking scoring function PLANTS_PLP_ [55]. A crystal structure of PF-543 exists docked within the active site of SphK1 (PDB ID: 4V24), and this protein was selected for docking [48].

Pyrrolidine quinoline-5,8-dione **21** and precursor **6** were chosen as potent and consistent SphK1 inhibitors and docked into the lipid-binding site of SphK1, with PF-543 providing a comparison docking score. The docked PF-543 ligand exhibited a score of −109.2 (Table 1). The addition of the pyrrolidone headgroup derived from the SphK1 selective inhibitor PF-543 improved the predicted docking score from −56.5 for **6** to −82.7 for **21**. A decrease in lipophilicity from PF-543 to **21** is also evident.

**Figure 8:**
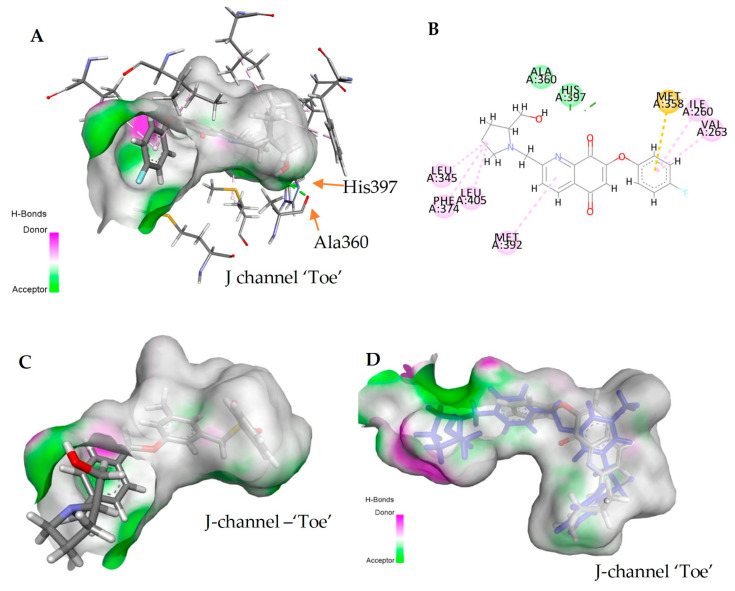
(**A**) Proposed interactions between fluoro analogue **21** and amino acid residues in the ligand-binding site of SphK1. (**B**) 2D representation of the 3D interactions described in (**A**). (green = hydrogen bond, pink = π-alkyl or alkyl, yellow = π-sulfur). (**C**) PF-543 in lipid-binding site. (**D**) PF-543 (blue) superimposed with **21** for docking comparison.

Further investigation into the binding pose of **21** suggests that the orientation of the pyrrolidine headgroup of **21** adopts a reversed orientation relative to PF-543 (Figure 8A,C,D). Unlike PF-543, which forms one hydrogen bond interaction with Asp264, **21** is predicted to engage in two hydrogen bonds with Ala360 and His397 located near the bottom ‘toe’ of the ‘J-channel’ (Figure 8A,B). Multiple hydrophobic interactions were observed throughout the ‘J-channel’, many of which are shared with PF-543, including the Met358 π-sulfur interaction, the Leu405 alkyl interaction, the Ile260 π-alkyl interaction and the Val263 π-alkyl interaction.

While the binding pose of **21** is unusual, it is worth noting that this predicted model is in agreement with the in vitro SphK1 inhibition (Figure 4), with the most potent inhibition observed from the *para*-substituted **4**, **6** and **7** moieties, which do not possess significant steric influence. Substituents in the *meta* position, or both the *para* and *meta* positions, would naturally result in poor SphK1 binding. Thus, preliminary modelling studies suggest that the pyrrolidine quinoline-5,8-dione framework can bind readily with the active site of SphK1 in silico, with promising hydrogen bonding interactions. There is an overlap with the bioassay data, and further hydrogen-bonding accepting regions are identified near the fluorine, which will be the scope of future work. Looking at Figure 1, this is an area of SphK that has not been targeted to date (components in red lack any terminal polar functionality) and is the focus of our current work.

### 2.7. Anticancer Screening of Quinoline-5,8-Diones

A preliminary anticancer study was conducted through the National Cancer Institute 60 human tumour cell line panel (NCI60) on a representative selection of the aryl-ether quinoline-5,8-diones (Table 2) [57]. Disappointingly, the mean cell percentage growth at a 10 µM concentration across the human cancer cell lines revealed no promising anticancer activity, with values ranging from 84–97% growth. In order to frame this, although PF-543 and opaganib have well-known anticancer activity, only opaganib has been assessed through the NCI cancer-screening platform (NSC796101) and is associated with a mean GI_50_ across 60 cell lines of 11.5 μM. While not unexpected, as active SphK inhibitors under clinical investigation are used in conjugation with a cytotoxic adjuvant therapy, there is further work necessary to improve potency and cellular activity.

It is worth noting that *para*-Br and -F analogues **6** and **12** exhibited specific activity against NCI-H522 (lung), ACHN (renal) and MDA-MB-468 (breast) cancer cell lines. For example, cell growths of −44% and −27% at 10 a µM concentration were recorded against the stage-two adenocarcinoma NSCLC cell line NCI-H522, which is sensitive to oxidative stress [58]. This may merit further investigation into the mechanisms of action.

## 3. Materials and Methods

### 3.1. Materials, Reagents and General Experimental Procedures

All solvents were distilled prior to use by the following methods: dichloromethane was distilled from phosphorus pentoxide, ethyl acetate was distilled from potassium carbonate and hexane was distilled prior to use. Organic phases were dried over anhydrous magnesium sulfate. All commercial reagents were procured from both international and local suppliers such as Fluorochem (Glossop, UK), Thermo Scientific (Dublin, Ireland) and Merck (Arklow, Ireland) and were used without further purification unless stated otherwise. Furthermore, 1H (300 MHz) and 13C (75 MHz) NMR spectra were recorded on a Bruker (Coventry, UK) Avance 300, 400 or 600 NMR spectrometer. All spectra were recorded at 20 °C in deuterated dimethylsulfoxide (DMSO-*d_6_*) or deuterated chloroform (CDCl_3_) using tetramethylsilane (TMS) as an internal standard unless otherwise stated. Chemical shifts (δH and δC) are reported in parts per million (ppm) relative to the reference peak. The order of citation in parentheses is (a) the number of protons, (b) multiplicity and (c) coupling constants (coupling constants (*J*) are reported in Hertz (Hz)). Infrared spectra were recorded as thin films on sodium chloride plates for oils or as potassium bromide (KBr) discs for solids on a Perkin Elmer (Dublin, Ireland) Spectrum 100 FT-IR spectrometer or a Perkin Elmer (Dublin, Ireland) Spectrum One FT-IR spectrometer. Nominal mass spectra were recorded on a Waters (Manchester, UK) Quattro Micro triple quadrupole spectrometer (QAA1202) in ESI mode using 50% acetonitrile–water containing 0.1% formic acid as eluent. High-resolution mass spectra (HRMS) were recorded on a Waters (Manchester, UK) LCT Premier Time-of-Flight spectrometer (KD160) or a Waters (Manchester, UK) Vion IMS mass spectrometer (SAA055 K) in ESI mode using 50% acetonitrile–water containing 0.1% formic acid as eluent. Samples (max. 1 mg) were dissolved in acetonitrile, water or 10% DMSO/acetonitrile. Melting points were measured on a uni-melt Thomas Hoover capillary melting point apparatus and were uncorrected. Thin-layer chromatography (TLC) was carried out on precoated silica gel plates (Merck 60 PF254). Visualisation was achieved by UV light detection (254 and 366 nm). Wet flash column chromatography was performed using Merck PF254 silica gel unless otherwise stated. The purity of each compound (>95%) was identified by LCMS.

### 3.2. Chemical Synthesis

#### 3.2.1. 5,7-Dibromo-2-methylquinolin-8-ol (**2**)

8-Hydroxyquinaldine **1** (6.02 g, 37.8 mmol) was dissolved in acetonitrile (200 mL) with stirring at room temperature. *N*-Bromosuccinimide (12.17 g, 68.4 mmol) was added portionwise. The mixture was stirred for 5 min, at which point a white precipitate was observed. Additional acetonitrile was added (200 mL), and the reaction mixture was allowed to stir for a further 1 h. The precipitate was isolated via suction filtration and washed with ice-cold acetonitrile (100 mL) to remove residual traces of succinimide. The sample was oven-dried at 80 °C, yielding the product as a light pink fluffy solid, which was used without further purification: **2** (9.89 g, 83%). m.p. 99.0–100.0 °C (lit. 126.9–128.5 °C) [33]; ν_max_/cm^−1^ (KBr): 2849, 2478, 1584, 1490, 1430, 1367, 1329, 1313, 1248, 1195, 1144, 932, 869, 736; δH (300 MHz, CDCl_3_): 2.76 (s, 3H), 7.41 (d, *J* = 8.6 Hz, 1H), 7.81 (s, 1H), 8.29 (d, *J* = 8.6 Hz, 1H); *m*/*z* (ESI-): 314.0 (M-H)^−^, 40%, 316.0 (M-H)^−^, 100%, 318.0 (M-H)^−^, 40%.

#### 3.2.2. 7-Bromo-2-methylquinoline-5,8-dione (**3**)

5,7-Dibromo-2-methylquinolin-8-ol **2** (1.48 g, 4.67 mmol) was dissolved portionwise in concentrated sulfuric acid (95–97%, 8.0 mL) with stirring, producing a yellow solution. The mixture was cooled to 0 °C using an ice bath. Nitric acid (65%, 0.90 mL, 14.0 mmol) was added dropwise over 1 min, producing a ruby red colour. The reaction was stirred for a further 30 min. After stirring, the reaction mixture was added to ice water (100 mL), resulting in a yellow solution. The product was extracted with dichloromethane (3 × 30 mL), washed with water (30 mL), dried over anhydrous magnesium sulfate and concentrated under reduced pressure, yielding the product as a pale orange solid, which was used without further purification: **3** (0.98 g, 84%). m.p. 150.0–152.0 °C (decomp.) (lit. 178.0 °C) [59]; ν_max_/cm^−1^ (KBr): 3045, 1685, 1656, 1581, 1429, 1386, 1310, 1280, 1129, 1104, 910, 847, 802, 711, 610; δH (300 MHz, CDCl_3_): 2.79 (s, 3H), 7.55 (s, 1H), 7.57 (d, *J* = 8.1 Hz, 1H), 8.29 (d, *J* = 8.1 Hz, 1H); *m*/*z* (ESI+): 252.1 (M+H)^+^, 95%, 254.1 (M+H)^+^, 100%.

#### 3.2.3. 7-(4-Methoxyphenoxy)-2-methyl-6,7-dihydroquinoline-5,8-dione (**4**)

Potassium carbonate (0.895 g, 6.47 mmol) and 4-methoxyphenol (0.251 g, 2.02 mmol) were added to dimethylformamide (10 mL) and stirred at room temperature for 10 min. 7-Bromo-2-methylquinoline-5,8-dione **3** (0.497 g, 1.97 mmol) was then charged, resulting in a dark brown suspension. The reaction mixture was then stirred for a further 2 h and poured over ice water (100 mL), forming a brown precipitate. The crude product was extracted with ethyl acetate (3 × 50 mL). The combined organic layers were washed with saturated sodium sulfite (3 × 50 mL) and brine (2 × 50 mL), dried over anhydrous magnesium sulfate and concentrated under reduced pressure, yielding a crude dark orange solid. Flash column chromatography was performed on this crude material (1:0 Hexane:Ethyl acetate—1:1 Hexane:Ethyl acetate), yielding the pure product as an orange solid: **4** (0.487 g, 84%) (Rf: 0.4, Hexane-Ethyl acetate 1:1). m.p. 151–152 °C; ν_max_/cm^−1^ (KBr): 3383, 3006, 2834, 1700, 1648, 1618, 1601, 1585, 1502, 1322, 1219, 1198, 1052; δH (300 MHz, CDCl_3_): 2.80 (s, 3H), 3.83 (s, 3H), 5.98 (s, 1H), 6.95–6.98 (m, 2H), 7.05–7.07 (m, 2H), 7.54 (d, *J* = 8.0 Hz, 1H), 8.26 (d, *J* = 8.0 Hz, 1H); δC (75 MHz, CDCl_3_): 25.2, 55.7, 112.3, 115.3 (2C), 121.9 (2C), 126.9, 128.0, 134.4, 145.9, 146.5, 157.9, 161.4, 164.7, 178.3, 183.9; *m*/*z* (ESI+): 296.2 (M+H)^+^, 100%; HRMS (ESI+): Exact mass calculated for C_17_H_14_NO_4_ 296.09173. Found 296.09201.

#### 3.2.4. 7-(3-Methoxyphenoxy)-2-methylquinoline-5,8-dione (**5**)

Potassium carbonate (0.83 g, 6.00 mmol) and 3-methoxyphenol (0.25 g, 2.01 mmol) were added to dimethylformamide (10 mL) and stirred at room temperature for 10 min. 7-bromo-2-methylquinoline-5,8-dione **3** (0.507 g, 2.01 mmol) was then charged, resulting in a dark brown suspension. The reaction mixture was then stirred for a further 2 h and poured over ice water (100 mL), forming a brown precipitate. The crude product was extracted with ethyl acetate (3 × 50 mL). The combined organic layers were washed with saturated sodium sulfite (3 × 50 mL) and brine (2 × 50 mL), dried over anhydrous magnesium sulfate and concentrated under reduced pressure, yielding a crude dark orange solid. Flash column chromatography was performed on this crude material (1:0 Hexane:Ethyl acetate—1:1 Hexane:Ethyl acetate), yielding the pure product as an orange solid: **5** (0.351 g, 60%) (Rf: 0.4, Hexane-Ethyl acetate 1:1). m.p. 153–155 °C; ν_max_/cm^−1^ (KBr): 3068, 3007, 2845, 1698, 1652, 1608, 1579, 1491, 1474, 1456, 1342, 1319, 1268,1219, 1172, 1136; δH (300 MHz, CDCl_3_): 2.81 (s, 3H), 3.83 (s, 3H), 6.05 (s, 1H), 6.69–6.75 (m, 2H), 6.87 (dd, *J* = 8.4, 2.0 Hz, 1H), 7.36 (t, *J* = 8.2 Hz, 1H), 7.55 (d, *J* = 8.0 Hz, 1H), 8.28 (d, *J* = 8.0 Hz, 1H); δC (75 MHz, CDCl_3_): 25.2, 55.5, 107.1, 112.4, 112.6, 113.0, 126.9, 128.0, 130.8, 134.5, 146.5, 153.5, 160.7, 161.3, 164.8, 178.2, 183.9; *m*/*z* (ESI+): 296.2 (M+H)^+^, 100%; HRMS (ESI+): Exact mass calculated for C_17_H_14_NO_4_ 296.0923. Found 296.0920.

#### 3.2.5. 7-(4-Fluorophenoxy)-2-methylquinoline-5,8-dione (**6**)

Potassium carbonate (0.661 g, 4.78 mmol) and 4-fluorophenol (0.178 g, 1.59 mmol) were added to dimethylformamide (8 mL) and stirred at room temperature for 10 min. 7-bromo-2-methylquinoline-5,8-dione **3** (0.403 g, 1.59 mmol) was then charged, resulting in a dark brown suspension. The reaction mixture was then stirred for a further 8 h and poured over ice water (100 mL), forming a light brown precipitate. The crude product was extracted with ethyl acetate (3 × 50 mL). The combined organic layers were washed with saturated sodium sulfite (3 × 50 mL) and brine (2 × 50 mL), dried over anhydrous magnesium sulfate and concentrated under reduced pressure, yielding a crude dark yellow solid. Flash column chromatography was performed on this crude material (1:0 Hexane:Ethyl acetate—4:6 Hexane:Ethyl acetate), yielding the pure product as a bright yellow solid: **6** (0.313 g, 70%) (Rf: 0.3, Hexane-Ethyl acetate 1:1). m.p. 205–206 °C (degrad.); ν_max_/cm^−1^ (KBr): 3367, 3299, 3070, 3041, 2994, 1693, 1659, 1594, 1585, 1501, 1338, 1314, 1237, 1215, 1190, 1139, 1051, 845, 836; δH (300 MHz, CDCl_3_): 2.80 (s, 3H), 5.97 (s, 1H), 7.13–7.17 (m, 4H), 7.55 (d, *J* = 8.0 Hz, 1H), 8.27 (d, *J* = 8.0 Hz, 1H); δC (75 MHz, CDCl_3_): 25.2, 112.5, 117.2 (d, *J* = 23.7 Hz, 2C), 122.6 (d, *J* = 8.5 Hz, 2C), 126.8, 128.1, 134.5, 146.4, 148.3 (d, *J* = 3.2 Hz), 160.6 (d, *J* = 248 Hz), 160.8, 164.9, 178.1, 183.7; δF (282 MHz, CDCl_3_): −114.9 [1F, tt, J 5.0, 5.0 Hz, C(4′)F]; *m*/*z* (ESI+): 284.2 (M+H)^+^, 100%; HRMS (ESI+): Exact mass calculated for C_16_H_11_FNO_3_ 284.07175. Found 287.07156.

#### 3.2.6. 2-Methyl-7-(4-(cyano)phenoxy)quinoline-5,8-dione (**7**)

Potassium carbonate (0.889 g, 6.40 mmol) and 4-cyanophenol (0.240 g, 2.01 mmol) were added to dimethylformamide (10 mL) and stirred at room temperature for 10 min. 7-bromo-2-methylquinoline-5,8-dione **3** (0.507 g, 2.00 mmol) was then charged, resulting in a dark brown/red suspension. The reaction mixture was then stirred for a further 3 h and poured over ice water (150 mL). The crude product was extracted with ethyl acetate (3 × 50 mL). The combined organic layers were washed with saturated sodium sulfite (3 × 50 mL) and brine (2 × 50 mL), dried over anhydrous magnesium sulfate and concentrated under reduced pressure, yielding a crude brown solid. Flash column chromatography was performed on this crude material (1:0 Hexane:Ethyl acetate—4:6 Hexane:Ethyl acetate), yielding the pure product as a bright yellow solid: **7** (0.381 g, 65%) (Rf: 0.3, Hexane-Ethyl acetate 1:1). m.p. 212–213 °C (degrad.); ν_max_/cm^−1^ (KBr): 3056, 2923, 2230, 1694, 1660, 1619, 1596, 1584, 1502, 1317, 1222, 1049; δH (300 MHz, CDCl_3_): 2.80 (s, 3H), 6.09 (s, 1H), 7.27–7.31 (m, 2H), 7.57 (d, *J* = 8.0 Hz, 1H), 7.78–7.81 (m, 2H), 8.29 (d, *J* = 8.0 Hz, 1H); δC (75 MHz, CDCl_3_): 25.2, 110.6, 114.3, 117.7, 121.9 (2C), 126.8, 128.3, 134.5, 134.6 (2C), 146.3, 156.2, 159.3, 165.2, 177.6, 183.4; *m*/*z* (ESI+): 291.2 (M+H)^+^, 100%; HRMS (ESI+): Exact mass calculated for C_17_H_11_N_2_O_3_ 291.07642. Found 291.07660.

#### 3.2.7. 7-(3,4-Dichlorophenoxy)-2-methylquinoline-5,8-dione (**8**)

Potassium carbonate (1.64 g, 11.9 mmol) and 3,4-dichlorophenol (0.65 g, 3.99 mmol) were added to dimethylformamide (20 mL) and stirred at room temperature for 10 min. 7-Bromo-2-methylquinoline-5,8-dione **3** (1.00 g, 3.98 mmol) was then charged, resulting in a dark brown suspension. The reaction mixture was then stirred for a further 20 h and poured over ice water (100 mL), forming a brown precipitate. The crude product was extracted with ethyl acetate (3 × 50 mL). The combined organic layers were washed with saturated sodium sulfite (3 × 50 mL) and brine (2 × 50 mL), dried over anhydrous magnesium sulfate and concentrated under reduced pressure, yielding a crude dark orange solid. Flash column chromatography was performed on this crude material (1:0 Hexane:Ethyl acetate—1:1 Hexane:Ethyl acetate), yielding the pure product as a yellow solid: **8** (0.256 g, 19%) (Rf: 0.5, Hexane-Ethyl acetate 1:1). m.p. 199–201 °C; ν_max_/cm^−1^ (KBr): 3364, 3301, 3069, 3051, 1692, 1616, 1582, 1466, 1392, 1257, 1054; δH (300 MHz, CDCl_3_): 2.80 (s, 3H), 6.04 (s, 1H), 7.04 (dd, *J* = 8.7, 2.8 Hz, 1H), 7.30 (d, *J* = 2.7 Hz, 1H), 7.56 (dd, *J* = 8.8, 8.0 Hz, 2H), 8.28 (d, *J* = 8.0 Hz, 1H); δC (75 MHz, CDCl_3_): 25.2, 113.2, 120.6, 123.3, 126.8, 128.2, 131.0, 131.8, 134.2, 134.6, 146.3, 151.3, 160.0, 165.1, 177.7, 183.5; *m*/*z* (ESI+): 334.0 (M+H)^+^, 45%; HRMS (ESI+): Exact mass calculated for C_16_H_10_^35^Cl_2_NO_3_ 334.0038. Found 334.0026.

#### 3.2.8. 7-(3,4-Difluorophenoxy)-2-methylquinoline-5,8-dione (**9**)

Potassium carbonate (1.64 g, 11.9 mmol) and 3,4-difluorophenol (0.52 g, 3.97 mmol) were added to dimethylformamide (20 mL) and stirred at room temperature for 10 min. 7-Bromo-2-methylquinoline-5,8-dione **3** (1.00 g, 3.97 mmol) was then charged, resulting in a dark brown suspension. The reaction mixture was then stirred for a further 20 h and poured over ice water (100 mL), forming a brown precipitate. The crude product was extracted with ethyl acetate (3 × 50 mL). The combined organic layers were washed with saturated sodium sulfite (3 × 50 mL) and brine (2 × 50 mL), dried over anhydrous magnesium sulfate and concentrated under reduced pressure, yielding a crude dark orange solid. Flash column chromatography was performed on this crude material (1:0 Hexane:Ethyl acetate—1:1 Hexane:Ethyl acetate), yielding the pure product as a yellow solid: **9** (0.481 g, 40%) (Rf: 0.3, Hexane-Ethyl acetate 1:1). m.p. 206–208 °C; ν_max_/cm^−1^ (KBr): 3362, 3300, 3067, 3041, 1690, 1658, 1618, 1510, 1437, 1377, 1318, 1251, 1204, 1052; δH (300 MHz, CDCl_3_): 2.80 (s, 3H), 6.01 (s, 1H), 6.91–6.97 (m, 1H), 7.01–7.08 (m, 1H), 7.24–7.33 (m, 1H), 7.56 (d, *J* = 8.0 Hz, 1H), 8.28 (d, *J* = 8.0 Hz, 1H); δC (75 MHz, CDCl_3_): 25.2, 111.2 (d, *J* = 19.4 Hz), 112.8, 117.3 (dd, *J* = 8.3, 3.6 Hz), 118.5 (dd, *J* = 19.4, 1.0 Hz), 126.8, 128.2, 134.5, 146.3, 148.1 [dd, *J* = 8.3, 3.6 Hz], 148.8 (dd, *J* = 251, 13.6 Hz), 150.7 (dd, *J* = 251, 13.6 Hz), 160.2, 165.1, 177.8, 183.5; *m*/*z* (ESI+): 302.2 (M+H)^+^, 100%; HRMS (ESI+): Exact mass calculated for C_16_H_10_F_2_NO_3_ 302.0620. Found 302.0629.

#### 3.2.9. 7-(3-Chloro-4-fluorophenoxy)-2-methylquinoline-5,8-dione (**10**)

Potassium carbonate (0.83 g, 6.00 mmol) and 3-chloro-4-fluorophenol (0.29 g, 2.01 mmol) were added to dimethylformamide (10 mL) and stirred at room temperature for 10 min. 7-Bromo-2-methylquinoline-5,8-dione **3** (0.50 g, 2.01 mmol) was then charged, resulting in a dark brown suspension. The reaction mixture was then stirred for a further 20 h and poured over ice water (100 mL), forming a brown precipitate. The crude product was extracted with ethyl acetate (3 × 50 mL). The combined organic layers were washed with saturated sodium sulfite (3 × 50 mL) and brine (2 × 50 mL), dried over anhydrous magnesium sulfate and concentrated under reduced pressure, yielding a crude dark orange solid. Flash column chromatography was performed on this crude material (1:0 Hexane:Ethyl acetate—1:1 Hexane:Ethyl acetate), yielding the pure product as a yellow solid: **10** (0.046 g, 7%) (Rf: 0.3, Hexane-Ethyl acetate 1:1). m.p. 200–202 °C; ν_max_/cm^−1^ (KBr): 3365, 3300, 3065, 3033, 1690, 1657, 1615, 1493, 1437, 1376, 1256, 1217, 1198, 1053; δH (300 MHz, CDCl_3_): 2.80 (s, 3H), 6.00 (s, 1H), 7.04–7.09 (m, 1H), 7.23–7.29 (m, 2H), 7.56 (d, *J* = 8.0 Hz, 1H), 8.28 (d, *J* = 8.0 Hz, 1H); δC (75 MHz, CDCl_3_): 25.2, 112.9, 118.0 (d, *J* = 23.1 Hz), 120.9 (d, *J* = 7.3 Hz), 122.7 (d, *J* = 19.6 Hz), 123.6, 126.8, 128.2, 134.5, 146.3, 148.4 (d, *J* = 3.4 Hz), 158.1, 160.3, 165.1, 177.8, 183.5; *m*/*z* (ESI+): 318.2 (M+H)^+^, 100%, 320.2 (M+H)^+^, 40%; HRMS (ESI+): Exact mass calculated for C_16_H_10_^35^ClFNO_3_ 318.0323. Found 318.0333.

#### 3.2.10. 7-(4-(2-Hydroxyethyl)phenoxy)-2-methylquinoline-5,8-dione (**11**)

Potassium carbonate (1.64 g, 11.8 mmol) and 2-(4-hydroxyphenol)-ethanol (0.55 g, 3.97 mmol) were added to dimethylformamide (20 mL) and stirred at room temperature for 10 min. 7-Bromo-2-methylquinoline-5,8-dione **3** (1.0 g, 3.97 mmol) was then charged, resulting in a dark brown suspension. The reaction mixture was then stirred for a further 20 h and poured over ice water (100 mL), forming a brown precipitate. The crude product was extracted with ethyl acetate (3 × 50 mL). The combined organic layers were washed with saturated sodium sulfite (3 × 50 mL) and brine (2 × 50 mL), dried over anhydrous magnesium sulfate and concentrated under reduced pressure, yielding a crude dark orange solid. Flash column chromatography was performed on this crude material (1:0 Hexane:Ethyl acetate—7:3 Hexane:Ethyl acetate), yielding the pure product as a yellow solid: **11** (0.288 g, 23%) (Rf: 0.2, Hexane-Ethyl acetate 1:1). m.p. 195–197 °C; ν_max_/cm^−1^ (KBr): 3404, 3100, 3060, 3020, 2960, 1693, 1610, 1517, 1462, 1442, 1376, 1248, 1089, 1071; δH (300 MHz, *_d6_*-DMSO): 2.68 (s, 3H), 3.04 (t, *J* = 7.0 Hz, 2H), 4.26 (t, *J* = 7.0 Hz, 2H), 6.42 (s, 1H), 6.74 (d, *J* = 8.4 Hz, 2H), 7.18 (d, *J* = 8.4 Hz, 2H), 7.72 (d, *J* = 6.0 Hz, 1H), 8.24 (d, *J* = 8.1 Hz, 1H), 9.27 (s, 1H); δC (75 MHz, *_d6_*-DMSO): 24.9, 33.7, 70.6, 109.9, 115.4, 115.6, 127.0, 128.0, 128.2, 130.1, 130.4, 134.4, 146.7, 156.4, 160.3, 163.9, 178.3, 184.4; *m*/*z* (ESI+): 310.3 (M+H)^+^, 80%; HRMS (ESI+): Exact mass calculated for C_18_H_16_NO_4_ 310.10738. Found 310.10697.

#### 3.2.11. 7-(4-Bromophenoxy)-2-methylquinoline-5,8-dione (**12**)

Potassium carbonate (0.885 g, 6.40 mmol) and 4-bromophenol (0.361 g, 2.09 mmol) were added to dimethylformamide (10 mL) and stirred at room temperature for 10 min. 7-bromo-2-methylquinoline-5,8-dione **3** (0.504 g, 1.99 mmol) was then charged, resulting in a dark brown suspension. The reaction mixture was then stirred for a further 6 h and poured over ice water (150 mL), forming a light brown precipitate. The crude product was extracted with ethyl acetate (3 × 50 mL). The combined organic layers were washed with saturated sodium sulfite (3 × 50 mL) and brine (2 × 50 mL), dried over anhydrous magnesium sulfate and concentrated under reduced pressure, yielding a crude dark yellow solid. Flash column chromatography was performed on this crude material (1:0 Hexane:Ethyl acetate—1:1 Hexane:Ethyl acetate), yielding the pure product as a yellow solid: **6** (0.4565 g, 66%) (Rf: 0.4, Hexane-Ethyl acetate 1:1). m.p. 174–176 °C; ν_max_/cm^−1^ (KBr): 3357, 3068, 2959, 2856, 1693, 1656, 1637, 1576, 1482, 1340, 1315, 1219, 1199, 1138, 1068, 1050, 1011, 843; δH (300 MHz, CDCl_3_): 2.80 (s, 3H), 5.99 (s, 1H), 7.04–7.07 (m, 2H), 7.55 (d, *J* = 8.0 Hz, 1H), 7.58–7.61 (m, 2H), 8.27 (d, *J* = 8.0 Hz, 1H); δC (75 MHz, CDCl_3_): 25.2, 112.7, 120.0, 122.9 (2C), 126.8, 128.1, 133.6 (2C), 134.5, 146.4, 151.6, 160.4, 165.0, 178.0, 183.6; *m*/*z* (ESI+): 344.1 (M+H)^+^, 80%, 346.1 (M+H)^+^, 100%; HRMS (ESI+): Exact mass calculated for C_16_H_11_^79^BrNO_3_ 343.99168. Found 343.99213.

#### 3.2.12. 7-(4-Methoxyphenoxy)-5,8-dioxo-5,8-dihydroquinoline-2-carbaldehyde (**13**)

7-(4-Methoxyphenoxy)-2-methyl-6,7-dihydroquinoline-5,8-dione **4** (0.147 g, 0.50 mmol) was dissolved in 1,4-dioxane (11 mL) at room temperature with stirring, resulting in an orange solution. Selenium dioxide (0.103 g, 0.93 mmol) was then added, and the reaction mixture was heated to 90 °C for 18 h, resulting in a dark orange solution with black precipitate. The reaction mixture was filtered through a Celite pad and washed through with dichloromethane (30 mL). The filtrate was concentrated under reduced pressure, yielding a dark orange oil. This crude material was re-dissolved in dichloromethane (30 mL) and washed with brine (2 × 15 mL), dried over anhydrous magnesium sulfate and concentrated under reduced pressure, yielding a crude orange solid. The crude solid was subjected to flash column chromatography (1:0 Hexane:Ethyl acetate—7:3 Hexane:Ethyl acetate), resulting in the product as an orange solid: **13** (0.0776 g, 50%) (Rf: 0.5, Hexane-Ethyl acetate 1:1). m.p. 230–233 °C (decomp.); ν_max_/cm^−1^ (KBr): 3402, 3061, 2963, 1703, 1704, 1650, 1601, 1505, 1317, 1225, 1196, 1128, 1040; δH (300 MHz, CDCl_3_): 3.85 (s, 3H), 6.13 (s, 1H), 6.98 (d, *J* = 9.1 Hz, 2H), 7.08 (d, *J* = 9.1 Hz, 2H), 8.31 (d, *J* = 8.0 Hz, 1H), 8.59 (d, *J* = 8.0 Hz, 1H), 10.34 (s, 1H); δC (75 MHz, CDCl_3_): 55.7, 112.9, 115.5 (2C), 121.8 (2C), 125.1, 131.2, 136.2, 145.6, 147.1, 155.5, 158.2, 162.0, 177.3, 182.7, 191.9; *m*/*z* (ESI+): 310.2 (M+H)^+^, 40%; HRMS (ESI+): Exact mass calculated for C_17_H_12_NO_5_ 310.07100. Found 310.07081.

#### 3.2.13. 7-(3-Methoxyphenoxy)-5,8-dioxo-5,8-dihydroquinoline-2-carbaldehyde (**14**)

7-(3-Methoxyphenoxy)-2-methylquinoline-5,8-dione **5** (0.303 g, 1.03 mmol) was dissolved in 1,4-dioxane (25 mL) with stirring at room temperature, resulting in a yellow solution. Selenium dioxide (0.227 g, 2.05 mmol) was then added. The reaction mixture was heated to 90 °C for 18 h, resulting in a yellow solution with a black precipitate. The solution was filtered into a Celite pad and washed with dichloromethane. Then, the solution was concentrated under reduced pressure, resulting in a red/orange powder. The powder was dissolved in dichloromethane (30 mL), and the solution was washed with brine (2 × 15 mL). The product was dried over anhydrous magnesium sulfate and concentrated under reduced pressure, yielding a red/orange powder. Flash column chromatography was performed on this crude material (1:0 Hexane:Ethyl acetate—1:1 Hexane:Ethyl acetate), yielding the pure product as a yellow/orange solid: **4** (0.085 g, 27%) (Rf: 0.6, Hexane-Ethyl acetate 1:1). m.p. 186–188 °C; ν_max_/cm_-1_ (KBr): 3376, 3065, 2962, 2922, 2839, 1711, 1696, 1657, 1610, 1586, 1487, 1319, 1221, 1168, 1126, 1046; δH (300 MHz, CDCl_3_): 3.84 (s, 3H), 6.18 (s, 1H), 6.70–6.77 (m, 2H), 6.89 (dd, *J* = 8.4, 2.4 Hz, 1H), 7.39 (t, *J* = 8.2 Hz, 1H), 8.31 (d, *J* = 8.0 Hz, 1H), 8.60 (dd, *J* = 8.0, 0.7 Hz, 1H), 10.34 (s, 1H); δC (75 MHz, CDCl_3_): 55.6, 107.0, 112.7, 112.8, 113.2, 125.1, 131.0, 131.2, 136.2, 147.1, 153.3, 155.5, 161.40, 161.43, 177.2, 182.7, 191.8; *m*/*z* (ESI+): 332.1 (M+Na)^+^, 84%, 350.1 (M+K)^+^, 100%; HRMS (ESI+): Exact mass calculated for C_17_H_11_NO_5_^23^Na 332.0535. Found 332.0523.

#### 3.2.14. 7-(4-Fluorophenoxy)-5,8-dioxo-5,8-dihydroquinoline-2-carbaldehyde (**15**)

7-(4-Fluorophenoxy)-2-methylquinoline-5,8-dione **8** (0.1507 g, 0.53 mmol) was dissolved in 1,4-dioxane (12 mL) at room temperature with stirring, resulting in a yellow solution. Selenium dioxide (0.121 g, 1.09 mmol) was then added, and the reaction mixture was heated to 90 °C for 18 h, resulting in an orange solution with black precipitate. The reaction mixture was filtered through a Celite pad and washed through with dichloromethane (30 mL). The filtrate was concentrated under reduced pressure, yielding a crude yellow solid. This crude material was re-dissolved in dichloromethane (30 mL) and washed with brine (2 × 15 mL), dried over anhydrous magnesium sulfate and concentrated under reduced pressure, yielding a crude yellow solid. The crude solid was subjected to flash column chromatography (1:0 Hexane:Ethyl acetate—1:1 Hexane:Ethyl acetate), resulting in the product as a pale yellow solid: **15** (0.0853 g, 54%) (Rf: 0.5, Hexane-Ethyl acetate 1:1). m.p. 219–220 °C (degrad.); ν_max_/cm^−1^ (KBr): 3078, 2872, 1718, 1706, 1654, 1608, 1593, 1503, 1351, 1382, 1217, 1037; δH (300 MHz, CDCl_3_): 6.11 (s, 1H), 7.16–7.20 (m, 4H), 8.32 (d, *J* = 8.0 Hz, 1H), 8.60 (dd, *J* = 8.0, 0.7 Hz, 1H), 10.34 (s, 1H); δC (75 MHz, CDCl_3_): 113.1, 117.4 (d, *J* = 24 Hz, 2C), 122.5 (d, *J* = 9.0 Hz, 2C), 125.2, 131.2, 136.2, 147.1, 148.1 (d, *J* = 2.8 Hz), 155.5, 160.9 (d, *J* = 247 Hz), 161.5, 177.0, 182.5, 191.8; *m*/*z* (ESI+): 298.1 (M+H)^+^, 25%, 320.1 (M+Na)^+^, 85%, 338.1 (M+K)^+^, 60%; HRMS (ESI+): Exact mass calculated for C_16_H_9_NO_4_F 298.05101. Found 298.05143.

#### 3.2.15. 7-(3,4-Dichlorophenoxy)-5,8-dioxo-5,8-dihydroquinoline-2-carbaldehyde (**17**)

7-(3,4-Dichlorophenoxy)-2-methylquinoline-5,8-dione **10** (0.122 g, 0.366 mmol) was dissolved in 1,4-dioxane (10 mL) with stirring at room temperature, resulting in a yellow solution. Selenium dioxide (0.081 g, 0.732 mmol) was then added. The reaction mixture was heated to 90 °C for 18 h, resulting in a yellow solution with a black precipitate. The solution was filtered into a Celite pad and washed with dichloromethane. Then, the solution was concentrated under reduced pressure, resulting in a red/orange powder. The powder was dissolved in dichloromethane (30 mL), and the solution was washed with brine (2 × 15 mL). The product was dried over anhydrous magnesium sulfate and concentrated under reduced pressure, yielding a red/orange powder. Flash column chromatography was performed on this crude material (1:0 Hexane:Ethyl acetate—7:3 Hexane:Ethyl acetate), yielding the pure product as a yellow/orange solid: **17** (0.056 g, 44%) (Rf: 0.7, Hexane-Ethyl acetate 1:1). m.p. 201–203 °C; ν_max_/cm^−1^ (KBr): 3391, 3052, 2924, 2854, 1722, 1704, 1652, 1614, 1585, 1467, 1253, 1042; δH (300 MHz, CDCl_3_): 6.18 (s, 1H), 7.07 (dd, *J* = 8.8, 2.7 Hz, 1H), 7.33 (d, *J* = 2.6 Hz, 1H), 7.59 (d, *J* = 8.8 Hz, 1H), 8.33 (d, *J* = 8.2 Hz, 1H), 8.61 (dd, *J* = 8.0, 0.8 Hz, 1H), 10.33 (d, *J* = 0.7 Hz, 1H); δC (75 MHz, CDCl_3_): 113.7, 120.5, 123.3, 125.3, 131.1, 131.4, 132.0, 134.4, 136.3, 147.0, 151.0, 155.6, 160.6, 176.7, 182.3, 191.7; *m*/*z* (ESI+): 365.9 (M+H)^+^, 60%, 367.9 (M+H)^+^, 40%, 369.9 (M+H)^+^, 30%; HRMS (ESI+): Exact mass calculated for C_16_H_10_^35^Cl_2_NO_5_ 365.9936. Found 365.9926.

#### 3.2.16. 7-(3,4-Difluorophenoxy)-5,8-dioxo-5,8-dihydroquinoline-2-carbaldehyde (**18**)

7-(3,4-Difluorophenoxy)-2-methylquinoline-5,8-dione **9** (0.168 g, 5.56 mmol) was dissolved in 1,4-dioxane (15 mL) with stirring at room temperature, resulting in a yellow solution. Selenium dioxide (0.124 g, 1.12 mmol) was then added. The reaction mixture was heated to 90 °C for 18 h, resulting in a yellow solution with a black precipitate. The solution was filtered into a Celite pad and washed with dichloromethane. Then, the solution was concentrated under reduced pressure, resulting in a red/orange powder. The powder was dissolved in dichloromethane (30 mL), and the solution was washed with brine (2 × 15 mL). The product was dried over anhydrous magnesium sulfate and concentrated under reduced pressure, yielding a yellow powder. Flash column chromatography was performed on this crude material (1:0 Hexane:Ethyl acetate—7:3 Hexane:Ethyl acetate), yielding the pure product as a yellow/orange solid: **18** (0.070 g, 40%) (Rf: 0.7, Hexane-Ethyl acetate 1:1). m.p. 194–196 °C; ν_max_/cm^−1^ (KBr): 3396, 3076, 3044, 2924, 2843, 1709, 1653, 1614, 1509, 1433, 1322, 1224, 1039; δH (300 MHz, CDCl_3_): 6.16 (s, 1H), 6.93–6.99 (m, 1H), 7.04–7.10 (m, 1H), 7.27–7.36 (m, 1H), 8.33 (d, *J* = 8.0 Hz, 1H), 8.61 (dd, *J* = 8.0, 0.8 Hz, 1H), 10.33 (d, *J* = 0.9 Hz, 1H); δC (75 MHz, CDCl_3_): 111.2 (d, *J* = 19.7 Hz), 113.5, 117.1 (d, *J* = 6.0 Hz), 118.7 (d, *J* = 19.7 Hz), 125.2, 131.1, 136.3, 147.0, 147.8 (dd, *J* = 6.0, 4.1 Hz), 149.1 (dd, *J* = 250, 13.5 Hz), 150.8 (dd, *J* = 250, 13.5 Hz), 155.6, 160.9, 176.7, 182.4, 191.7; *m*/*z* (ESI-): 316.2 (M-H)^−^, 47%; HRMS (ESI-): Exact mass calculated for C_16_H_8_F_2_NO_4_ 316.0421. Found 316.0410.

#### 3.2.17. 7-(4-Bromophenoxy)-5,8-dioxo-5,8-dihydroquinoline-2-carbaldehyde (**19**)

7-(4-bromophenoxy)-2-methylquinoline-5,8-dione **12** (0.413 g, 1.20 mmol) was dissolved in 1,4-dioxane (30 mL) at room temperature with stirring, resulting in an orange solution. Selenium dioxide (0.271 g, 2.44 mmol) was then added, and the reaction mixture was heated to 90 °C for 18 h, resulting in an orange solution with black precipitate. The reaction mixture was filtered through a Celite pad and washed through with dichloromethane (50 mL). The filtrate was concentrated under reduced pressure, yielding a crude yellow solid. This crude material was re-dissolved in dichloromethane (50 mL) and washed with brine (2 × 30 mL), dried over anhydrous magnesium sulfate and concentrated under reduced pressure, yielding a crude yellow solid. The crude solid was subjected to flash column chromatography (1:0 Hexane:Ethyl acetate—7:3 Hexane:Ethyl acetate), resulting in the product as a fluffy yellow solid: **19** (0.391 g, 91%) (Rf: 0.5, Hexane-Ethyl acetate 1:1). m.p. 230–232 °C (degrad.); ν_max_/cm^−1^ (KBr): 3063, 2868, 1718, 1704, 1647, 1610, 1578, 1563, 1482, 1354, 1320, 1235, 1200, 1039; δH (300 MHz, CDCl_3_): 6.13 (s, 1H), 7.07 (d, *J* = 9.0 Hz, 2H), 7.63 (d, *J* = 9.0 Hz, 2H), 8.32 (d, *J* = 8.0 Hz, 1H), 8.60 (dd, *J* = 8.0, 0.8 Hz, 1H), 10.33 (d, *J* = 0.8 Hz, 1H); δC (75 MHz, CDCl_3_): 113.3, 120.4, 122.8 (2C), 125.2, 131.2, 133.8 (2C), 136.2, 147.0, 151.4, 155.6, 161.0, 176.9, 182.4, 191.7; *m*/*z* (ESI+): 358.0 (M+H)^+^, 20%, 260.0 (M+H)^+^, 20%. HRMS (ESI+): Exact mass calculated for C_16_H_9_^79^BrNO_4_ 357.97095. Found 357.97081.

#### 3.2.18. (*R*)-2-((2-(Hydroxymethyl)pyrrolidin-1-yl)methyl)-7-(4-methoxyphenoxy)quinoline-5,8-dione (**20**)

Sodium triacetoxyborohydride (0.187 g, 0.88 mmol) was added to a solution of 7-(4-Methoxyphenoxy)-5,8-dioxo-5,8-dihydroquinoline-2-carbaldehyde **13** (0.171 g, 0.55 mmol) in dichloroethane (14 mL). D-Prolinol (0.059 g, 0.58 mmol) was weighed, and dichloroethane (2 mL) was added to the solution. This solution was added dropwise to the reaction mixture over 2 min, resulting in a cloudy brown suspension. The solution was stirred at room temperature for 12 h, resulting in an orange suspension. Dichloromethane (30 mL) was added, and the organic layer was washed with saturated sodium bicarbonate (3 × 30 mL), dried over anhydrous magnesium sulfate and concentrated under reduced pressure to yield a crude brown oil. The crude project was subjected to flash chromatography (0:1 Methanol: Dichloromethane—1:99 Methanol: Dichloromethane), resulting in an orange oil: **20** (0.0797 g, 37%); δH (300 MHz, CDCl_3_): 1.78–1.86 (m, 1H), 1.97–2.02 (m, 1H), 2.51 (q, *J* = 8.9 Hz, 1H), 2.92–2.96 (m, 1H), 3.08–3.12 (m, 1H), 3.18 (bs, 1H), 3.52 (dd, *J* = 11.2, 3.5 Hz, 1H), 3.64 (dd, *J* = 11.2, 3.5 Hz, 1H), 3.84 (s, 3H), 3.98 (d, *J* = 15.4 Hz, 1H), 4.31 (d, *J* = 15.4 Hz 1H), 6.00 (s, 1H), 6.95–6.99 (m, 2H), 7.03–7.07 (m, 2H), 7.84 (d, *J* = 8.0 Hz, 1H), 8.36 (d, *J* = 8.0 Hz 1H); δC (75 MHz, CDCl_3_): 23.7, 27.4, 55.1, 55.7, 60.4, 62.7, 65.5, 112.4, 115.4 (2C), 121.9 (2C), 127.0, 127.8, 134.9, 145.8, 146.3, 158.0, 161.4, 165.9, 178.1, 183.7; *m*/*z* (ESI+): 395.2 (M+H)^+^, 100%; HRMS (ESI+): Exact mass calculated for C_22_H_22_N_2_O_5_ 395.16015. Found 395.15927.

#### 3.2.19. (*R*)-7-(4-Fluorophenoxy)-2-((2-(hydroxymethyl)pyrrolidin-1-yl)methyl)quinoline-5,8-dione (**21**)

Sodium triacetoxyborohydride (0.0759 g, 0.36 mmol) was added to a solution of 7-(4 Fluorophenoxy)-5,8-dioxo-5,8-dihydroquinoline-2-carbaldehyde **15** (0.0651 g, 0.22 mmol) in dichloroethane (4 mL). D-Prolinol (0.0216 g, 0.21 mmol) was weighed, and dichloroethane (1 mL) was added to the solution. This solution was added dropwise to the reaction mixture over 2 min, resulting in a cloudy brown suspension. The solution was stirred at room temperature for 12 h, resulting in an orange suspension. Dichloromethane (15 mL) was added, and the organic layer was washed with saturated sodium bicarbonate (3 × 10 mL), dried over anhydrous magnesium sulfate and concentrated under reduced pressure to yield a crude brown oil. The crude project was subjected to flash chromatography (0:1 Methanol:Dichloromethane—3:97 Methanol:Dichloromethane), resulting in a light brown oil: **21** (0.031 g, 38%) (Rf: 0.6, Hexane-Ethyl acetate 1:1). δH (300 MHz, CDCl_3_): 1.76–2.01 (m, 2H), 2.50 (q, *J* = 8.9 Hz, 1H), 2.83 (bs, 1H), 2.90–2.93 (m, 1H), 3.07–3.09 (m, 1H), 3.50 (dd, *J* = 11.2, 3.5 Hz, 1H), 3.63 (dd, *J* = 11.2, 3.5 Hz, 1H), 3.97 (d, *J* = 15.2 Hz, 1H), 4.28 (d, *J* = 15.2 Hz, 1H), 5.98 (s, 1H), 7.12–7.20 (m, 4H), 7.85 (d, *J* = 8.2 Hz, 1H), 8.36 (d, *J* = 8.2 Hz, 1H); δC (75 MHz, CDCl_3_): 23.7, 27.4, 55.1, 60.4, 62.7, 65.5, 112.6, 117.2 (d, *J* = 23.7 Hz, 2C), 122.6 (d, *J* = 6.7 Hz, 2C), 127.1, 127.7, 135.0, 146.2, 148.3 (d, *J* = 2.9 Hz), 160.7 (d, *J* = 247 Hz), 160.9, 166.1, 177.8, 183.6; *m*/*z* (ESI+): 383.2 (M+H)^+^, 95%, 384.2 (M+2H)^+^, 25%; HRMS (ESI+): Exact mass calculated for C_21_H_19_FN_2_O_4_ 383.14016. Found 383.13978.

### 3.3. Sphingosine Kinase Assays

A fluorometric assay was developed using a sphingosine derivative labelled with a 7-nitrobenz-2-oxa-1,3-diazole moiety (NBD-Sphingosine, Sigma-Aldrich, Arklow, Ireland, #810205P) and recombinant human SphK1 and SphK2 enzymes (Sigma-Aldrich, Arklow, Ireland, #SRP0283, #SRP0287, respectively) [41]. The relevant enzymes and inhibitors were incubated in total reaction volumes of 100 µL with 10 µM NBD-sphingosine, 500 µM 4-deoxypyridoxine and 1 mM Mg-ATP in assay buffer (100 mM Tris/HCl (pH 7.4), 150 mM NaCl, 2 mM Na_3_VO_4_ and 10 mM NaF) for 30 min at 37 °C. A reaction mixture containing no test SphK inhibitor served as the positive control, while a reaction mixture containing no Mg-ATP served as the negative control. Post-incubation, 100 µL of potassium phosphate buffer, pH 8.5, was added to each Eppendorf, followed by 500 µL of 2:1 Chloroform/Methanol. After brief mixing with a vortex, phases were separated by centrifugation at 2000 rpm for 5 min. The aqueous phase (100 µL) of each sample was then transferred to wells of a black 96-well flat-bottom plate, and fluorescent intensity was measured at Ex/Em 485/535 nm using a Wallac Victor2 Multilabel plate reader (Perkin Elmer, Dublin, Ireland). Data are expressed as mean SD from a minimum of 2 independent experiments. To calculate the concentrations of fluorescent product generated, a standard curve of NBD-S1P (Sigma-Aldrich, #810207X) was set up in the range of 0.1–10 µM. From this, the IC_50_ values of several test inhibitors were calculated.

### 3.4. Cancer Cell Growth Assays (NCI60 Screening)

One-dose study: Tested compounds were initially solubilised in DMSO, diluted into RPMI 1640 and 5% fetal bovine serum/L-glutamine and added to 96-well plates containing cell lines previously cultured for 24 h. After 48 h of incubation, the media were removed, and the cells were fixed and stained with sulforhodamine B to determine overall percent growth/total protein content. Unbound dye was removed with five washes of 1% acetic acid, and the plates were allowed to air dry. The dye was then resolubilised in Tris buffer, and the colourimetric absorbance was measured (515 nm). Growth inhibition was measured relative to the response generated from proliferating cells cultured under identical conditions for 48 h. Data from one-dose experiments pertain to the percentage growth at 10 μM [60,61].

## 4. Conclusions

A series of novel C(7)-aryl-ether quinoline-5,8-diones were synthesised in yields of up to 84%. Structural modifications to the aryl region were performed to probe SphK and anticancer activities. This is the first time quinoline-5,8-diones have been reported to possess both SphK1 and SphK2 inhibitory activity. *Para*-substituted quinoline-5,8-diones **6** and **7** exhibited inhibition of both SphK1 and SphK2 (>50%), which is comparable to the clinically used anticancer agent opaganib, and **4** identified a level of selectivity for SphK1. The introduction of *meta* halogen substituents resulted in lower yields and reduced SphK activity.

Encouraged by these preliminary findings, a pyrrolidine headgroup was adapted from PF-543 and designed in the quinoline-5,8-dione framework to improve SphK1 inhibition. Two novel pyrrolidine quinoline-5,8-dione analogues were synthesised, **20** and **21,** which were accessed through novel C(2)-activated formyl intermediates. An improvement in SphK1 binding efficacy for **21** (70%) was observed relative to the parent compound **6** (54%). Molecular modelling was undertaken on pyrrolidine quinoline-5,8-dione **21,** revealing low binding energies and no major unfavourable steric hindrance. Compound **21** is revealed to access a polar region previously unknown for SphK inhibitors, which will be further developed in the future. Compound **4** is identified with an IC_50_ of 30 and 8 µM, respectively, against Sphk1 and SphK2 (and **20** with values of 42 and 13 µM, respectively). Although minimal anticancer activity was observed in vitro, low micromolar dual SphK1/2 inhibition with the quinoline-5,8-dione framework has been identified for the first time with a plausible binding mode to guide future efforts.

## Figures and Tables

**Table 1:** Pyrrolidine quinoline-5,8-dione **21** and precursor **6** docking scores within SphK1 ligand-binding site, with PF-543 as a docking reference. Negative scores correspond to a greater binding efficacy. ClogP was calculated using SwissADME [56].

Entry	Score	CLogP
**PF-543**	−109.2	4.75
**6**	−56.5	3.29
**21**	−82.7	2.41

**Table 2:** NCI60 one-dose anticancer screen of compounds **4**–**7** and **12**.

Compound	4	5	6	7	12
**Mean Growth (%) ^1^**	84.4	88.5	88.2	97.3	90.5

^1^ Compounds screened against the NCI-60 cell line panel at 10 μM concentration, value given is the mean growth percentage across 60 cell lines.

## Data Availability

All data are available through Supplementary Materials or by request.

## Supplementary Materials

The following supporting information can be downloaded at: https://www.mdpi.com/article/10.3390/ph18020268/s1. Data S1.1. ^1^H and ^13^C NMR Supporting Information for compounds **2**–**21**; Figure S1. HPLC chromatogram of compound **4**. Figure S2. HPLC chromatograms of compound **20**. Figure S3. NBD-S1P Fluorescence Standard Curve. Figure S4. Assay validation and standards. Figure S5. In silico ADME parameters of **4**. Figure S6. In silico ADME parameters of **20**. Figure S7 NCI One dose data for Compound **4**. Figure S8. NCI One dose data for Compound **5**. Figure S9. NCI One dose data for Compound **6**. Figure S10. NCI One dose data for Compound **7**. Figure S11. NCI One dose data for Compound **12**. Table S1. Peak purity of HPLC chromatogram for compounds **4** and **20**. Table S2. SK inhibition assay validation with control compounds PF-543 and ABC284640. Table S3. SK inhibition assay data for **5**-**7**, **11** and **20**. Table S4. SK inhibition assay data for **8**-**10**, **14**-**15** and **18**-**19**. Table S5. SK inhibition assay data for **4**, **13** and **21**. Table S6. In silico SphK1 Docking Score and Lipophilicity.

## Abbreviations

CLK1: CDC Like Kinase 1; DHS, dihydrosphingosine; DMS, dimethyl sphingosine; Met, Mesenchymal Epithelial Transition; MST2, Mammalian Ste-20-like Kinase 2; NQO1, NAD(P)H:quinone oxidoreductase 1; NBD, 7-nitrobenz-2-oxa-1,3-diazole; NCI, National Cancer Institute; PDB, Protein Data Bank; PLANTS, Protein−Ligand ANT System; PLP, Piecewise Linear Potential; SphK, Sphingosine Kinase; S1P, sphingosine-1-phosphate.
