# Supplementary material for: Discovery of Sphingosine Kinase Inhibition by Modified Quinoline-5,8-Diones
Source: Pharmaceuticals (Basel). 2025 Feb 18;18(2):268. doi: 10.3390/ph18020268 (PMC11859825; doi:10.3390/ph18020268)

Supplementary Information

Discovery of Sphingosine Kinase inhibition by modified Quinoline-5,8-diones

Ryan D. Kruschel[1], Kyle Malone[2], Alison N. Walsh,[1] Christian Waeber[2,3], Florence O. McCarthy [1,*]

[1] School of Chemistry, Analytical and Biological Chemistry Research Facility, University College Cork, Cork T12 K8AF, Ireland.

[2] School of Pharmacy, University College Cork, Pharmacy Building, College Road, Cork, Ireland, T12K8AF.

[3] Department of Pharmacology and Therapeutics, School of Medicine, University College Cork, Ireland T12 XF62

[*] Corresponding author f.mccarthy@ucc.ie (Florence O. McCarthy).

Contents

**S1: Chemical characterisation**	**Page**
**S1.1** ^{1}H and ^{13}C NMR Supporting Information for compounds **2-21**	S2
**S1.2** HPLC purity of **4** and **20** (*Figure S1-S2; Table S1*)	S20
**S2: Sphingosine kinase inhibitory assay data**	
**S2.1** Assay Validation and standards (*Figure S3-S4; Table S2*)	S21
**S2.2** SK inhibition assay data for **4**-**21** (Table S3-S5)	S22
**S3: Calculated Data**	
S3.1 In silico SphK1 Docking Score and Lipophilicity (*Table S6*)	S24
**S3.2** In silico ADME parameters of **4** (*Figure S5*)	S25
**S3.3** In silico ADME parameters of **20** (*Figure S6*)	S25
**S4: NCI 60 Cell Line Growth Percent for selected compounds at 10µM**	
**S4.1** NCI One dose data for Compound **4** (*Figure S7*)	S26
**S4.2** NCI One dose data for Compound **5** (*Figure S8*)	S27
**S4.3** NCI One dose data for Compound **6** (*Figure S9*)	S28
**S4.4** NCI One dose data for Compound **7** (*Figure S10*)	S29
**S4.5** NCI One dose data for Compound **12** (*Figure S11*)	S30

S1: **Chemical characterisation**

S1.1 **^{1}H and ^{13}C NMR Supporting Information for compounds 2-21**

Compound 2

^{1}H NMR

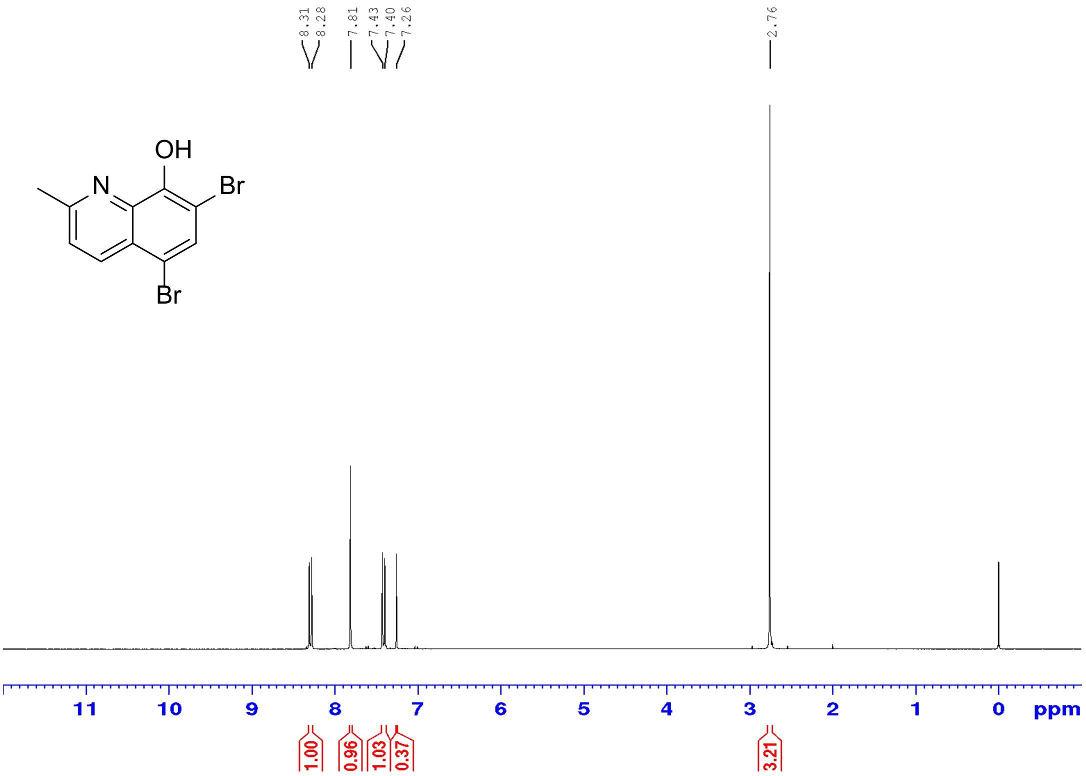

Compound 3

^{1}H NMR

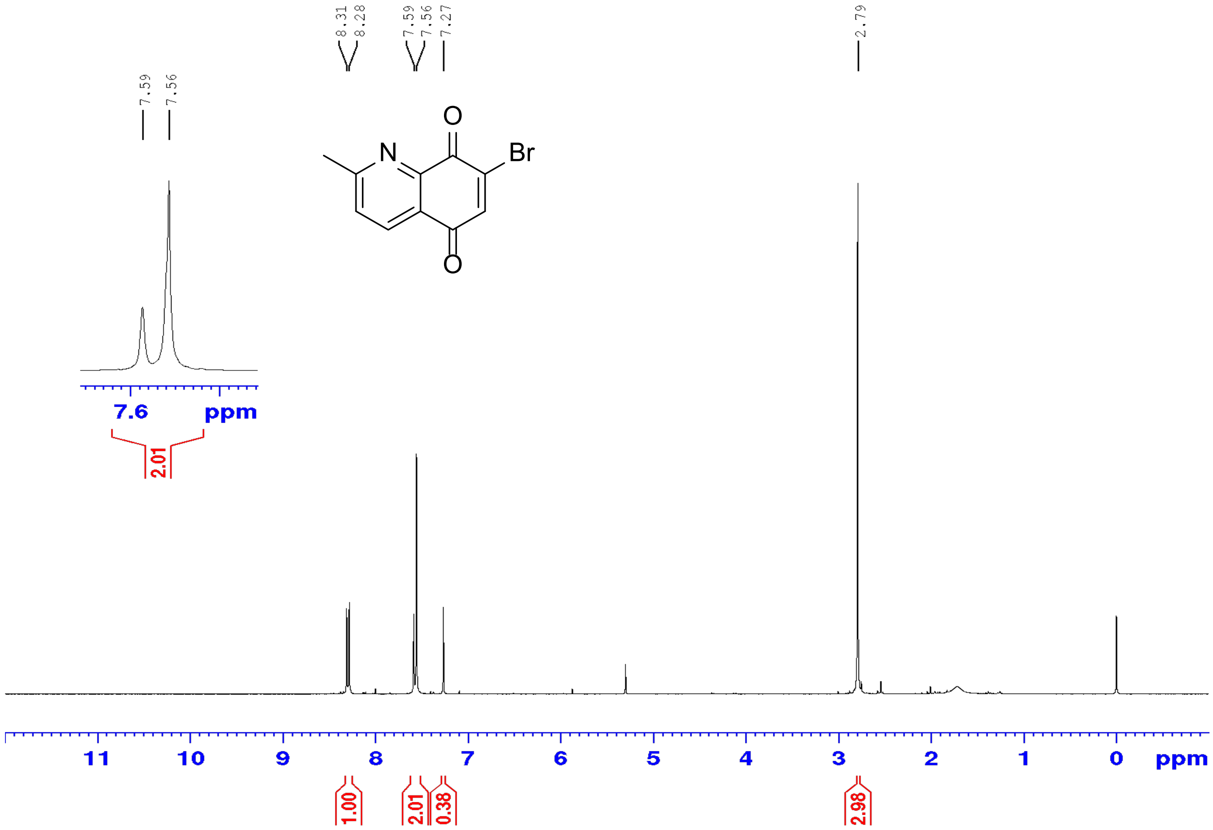

Compound 4
^{1}H NMR

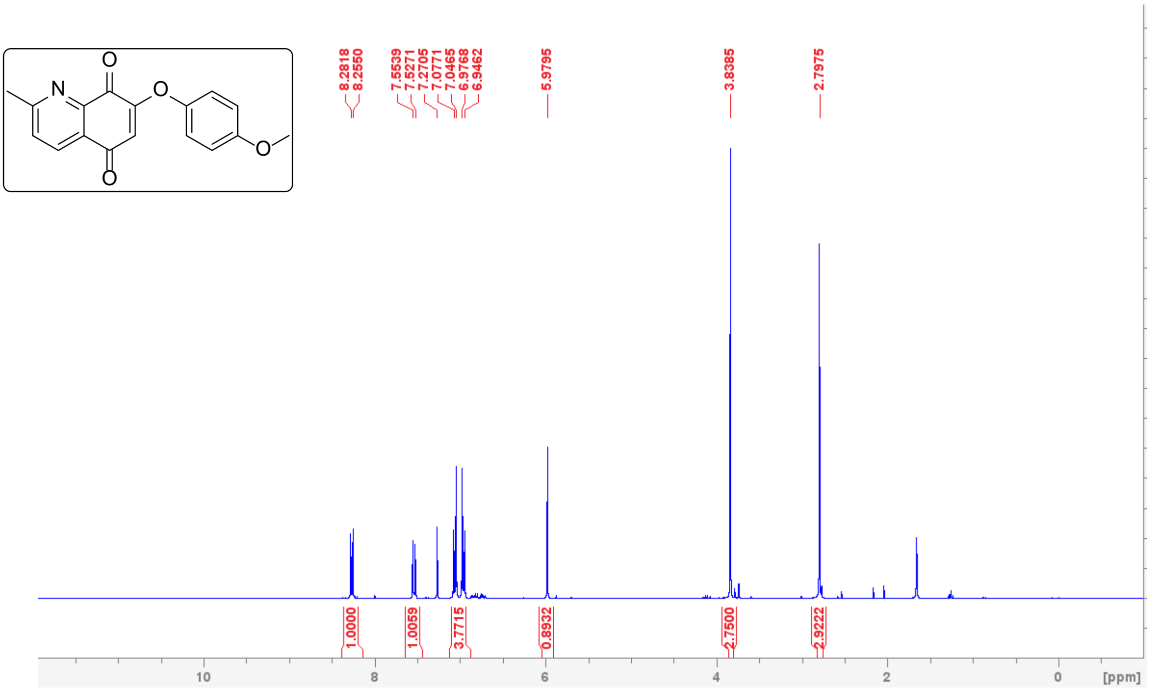

Compound 4
^{13}C NMR

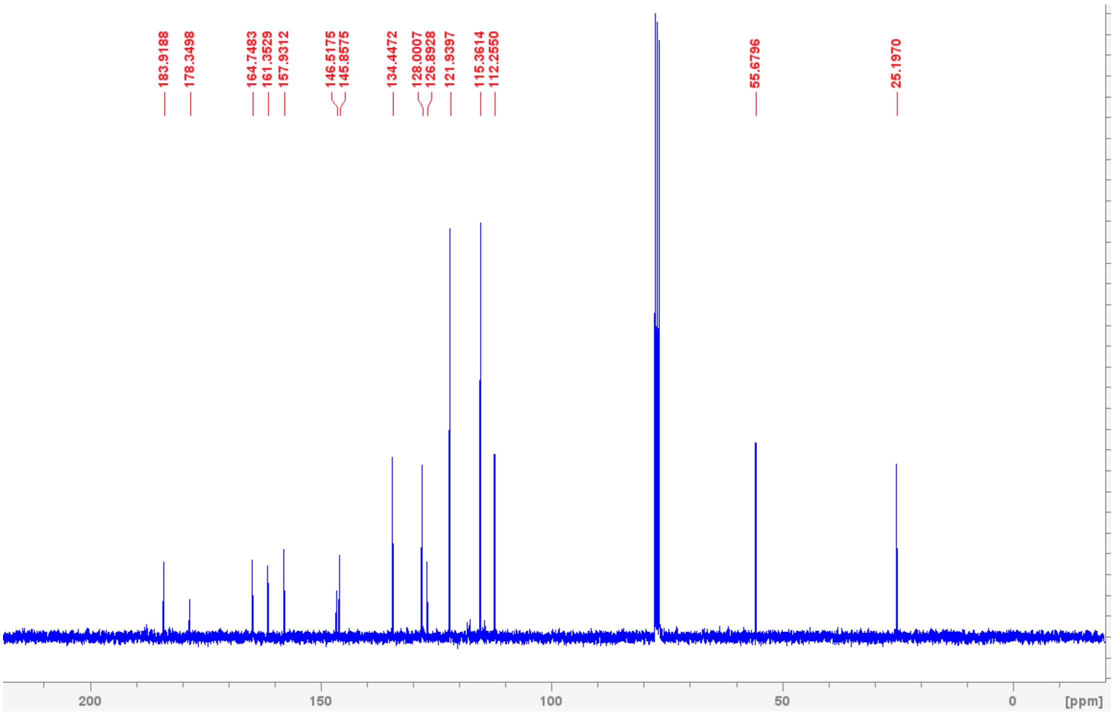

Compound 5
^{1}H NMR

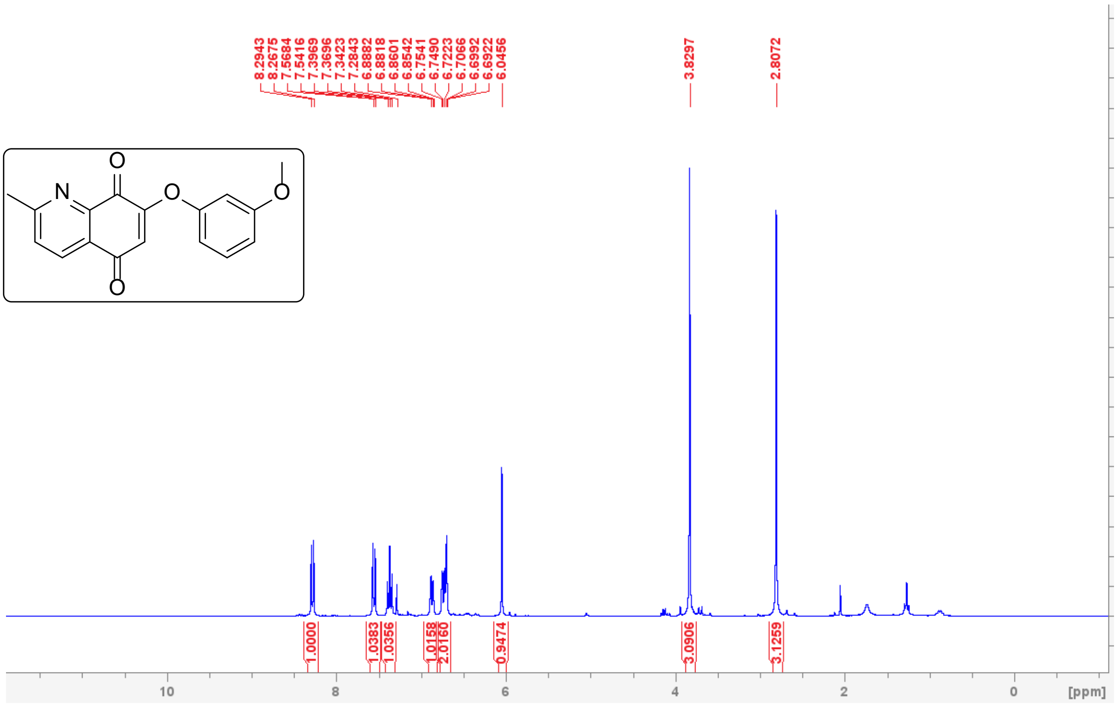

Compound 5
^{13}C NMR

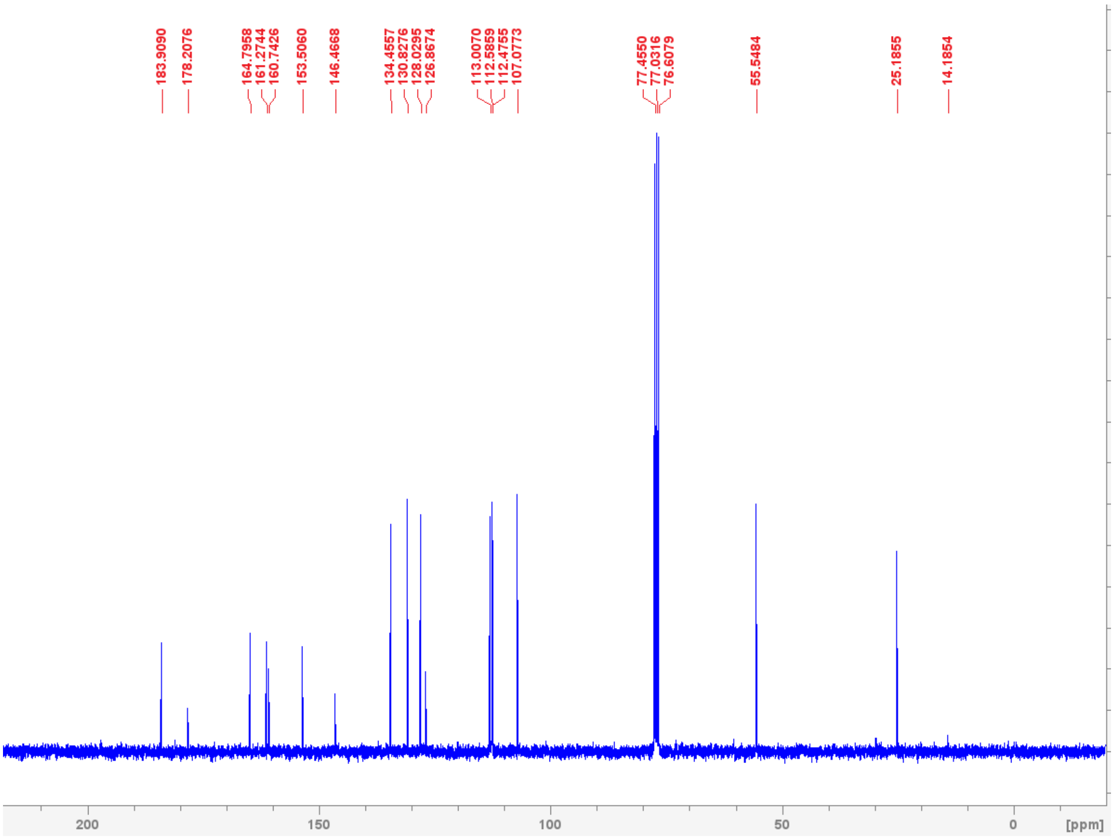

Compound 6

^{1}H NMR

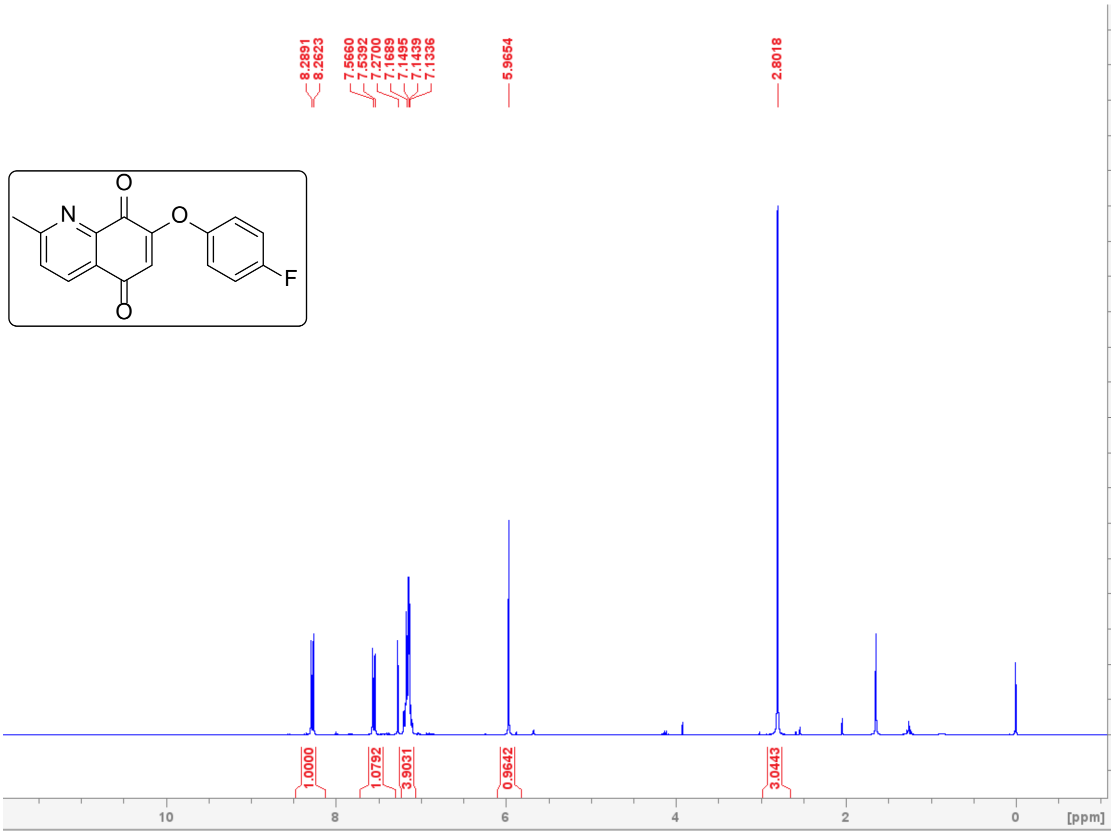

Compound 6

^{13}C NMR

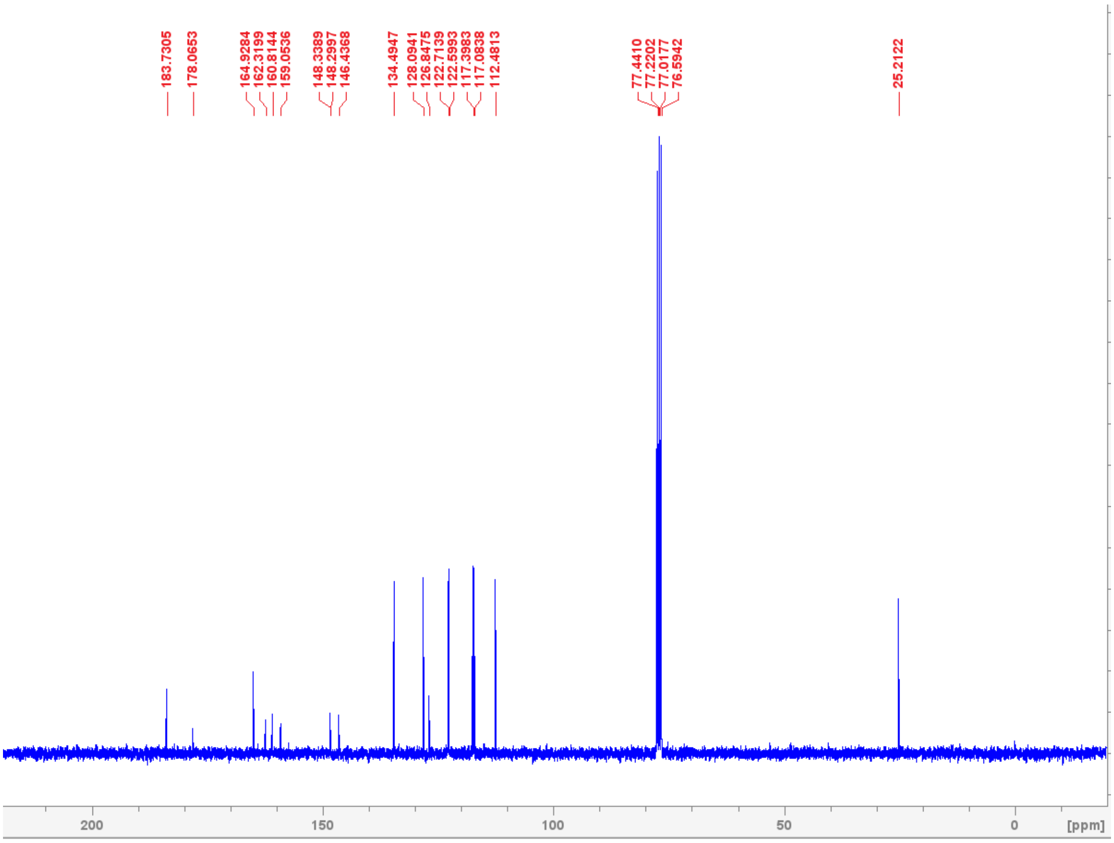

Compound 7
^{1}H NMR

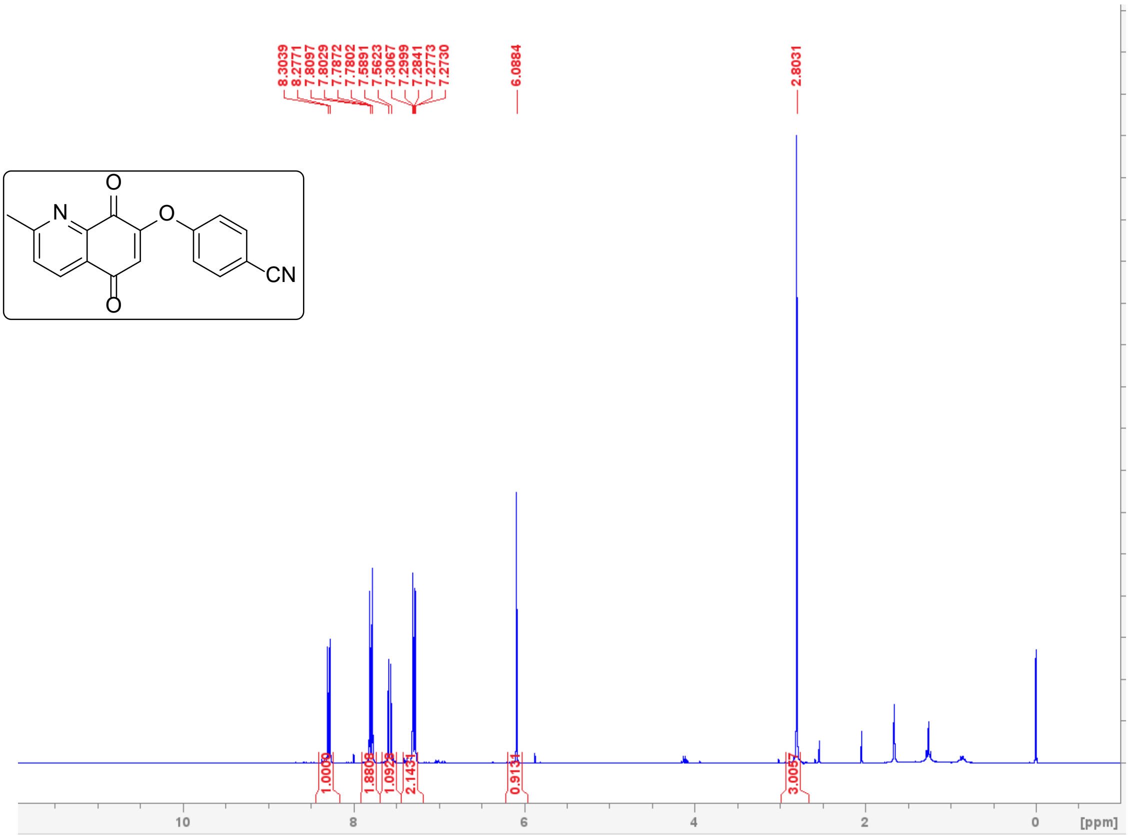

Compound 7
^{13}C NMR

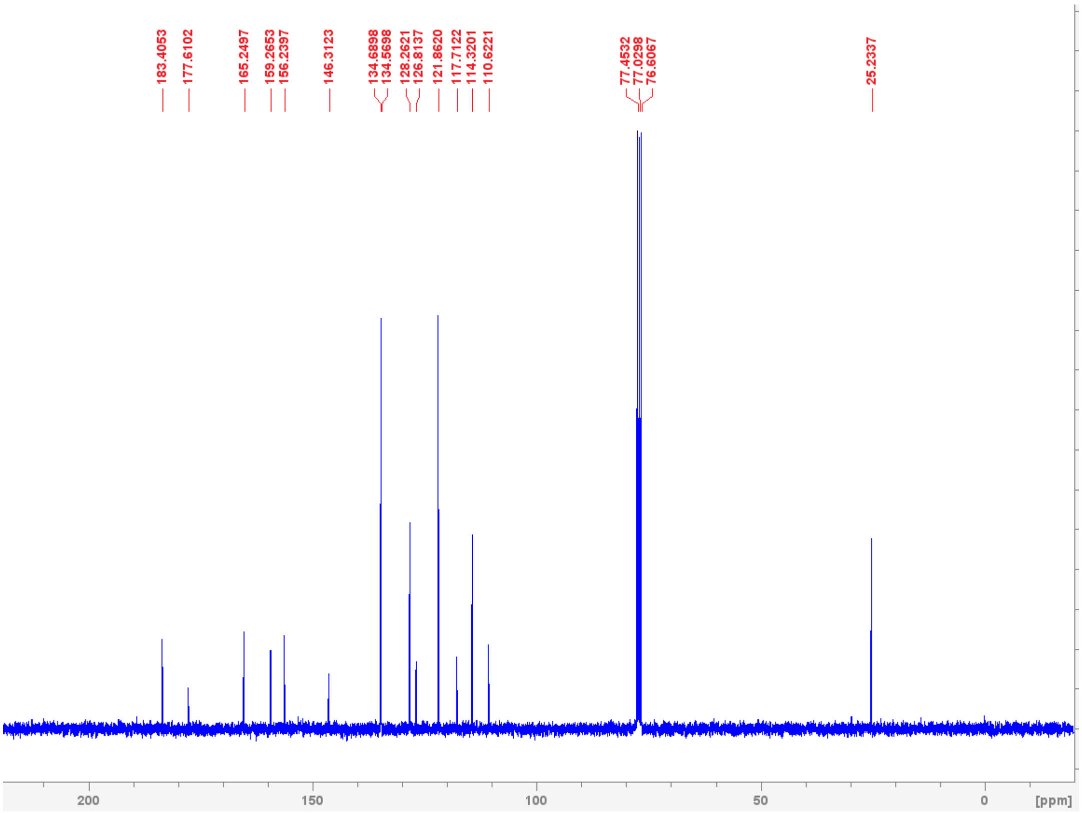

Compound 8

^{1}H NMR

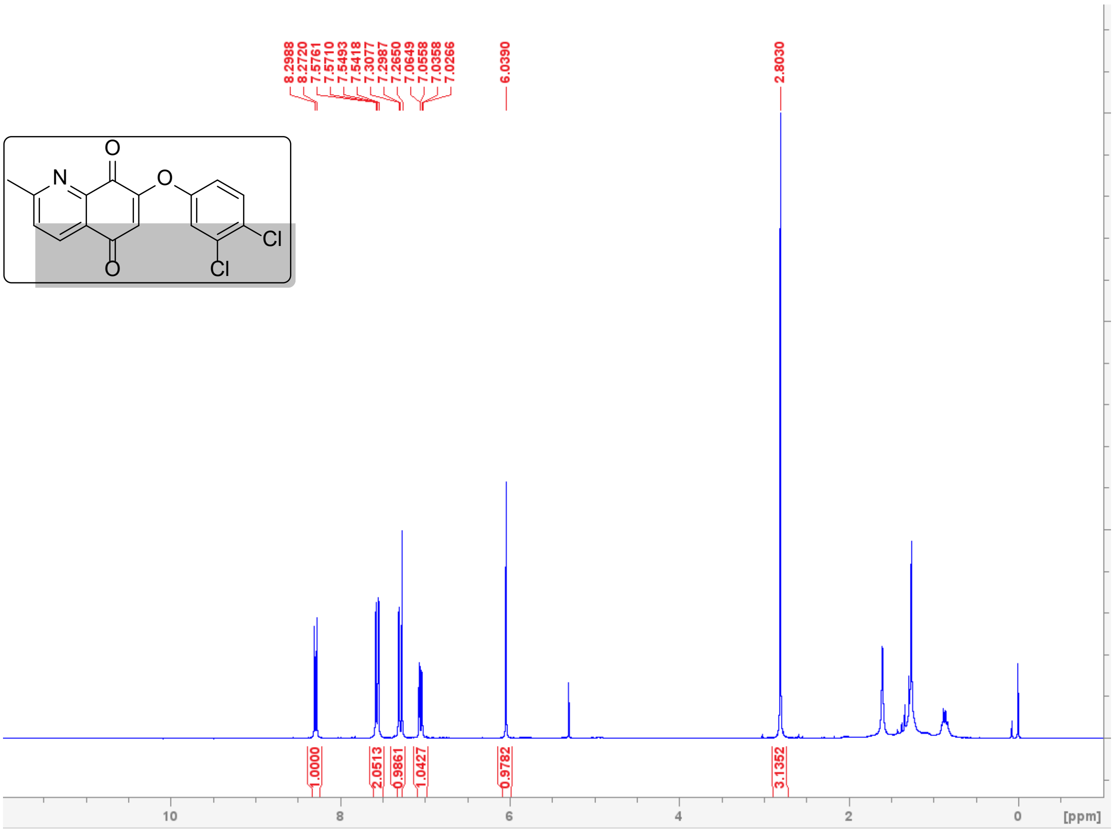

Compound 8

^{13}C NMR

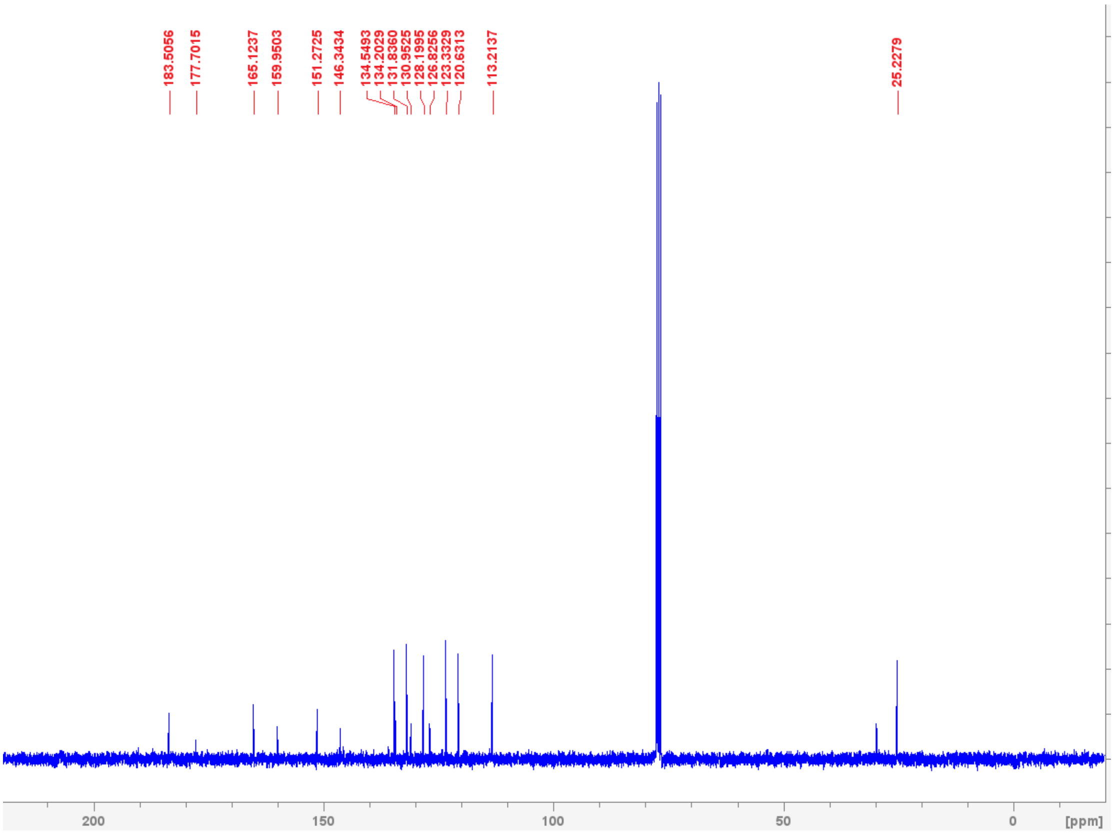

Compound 9

^{1}H NMR

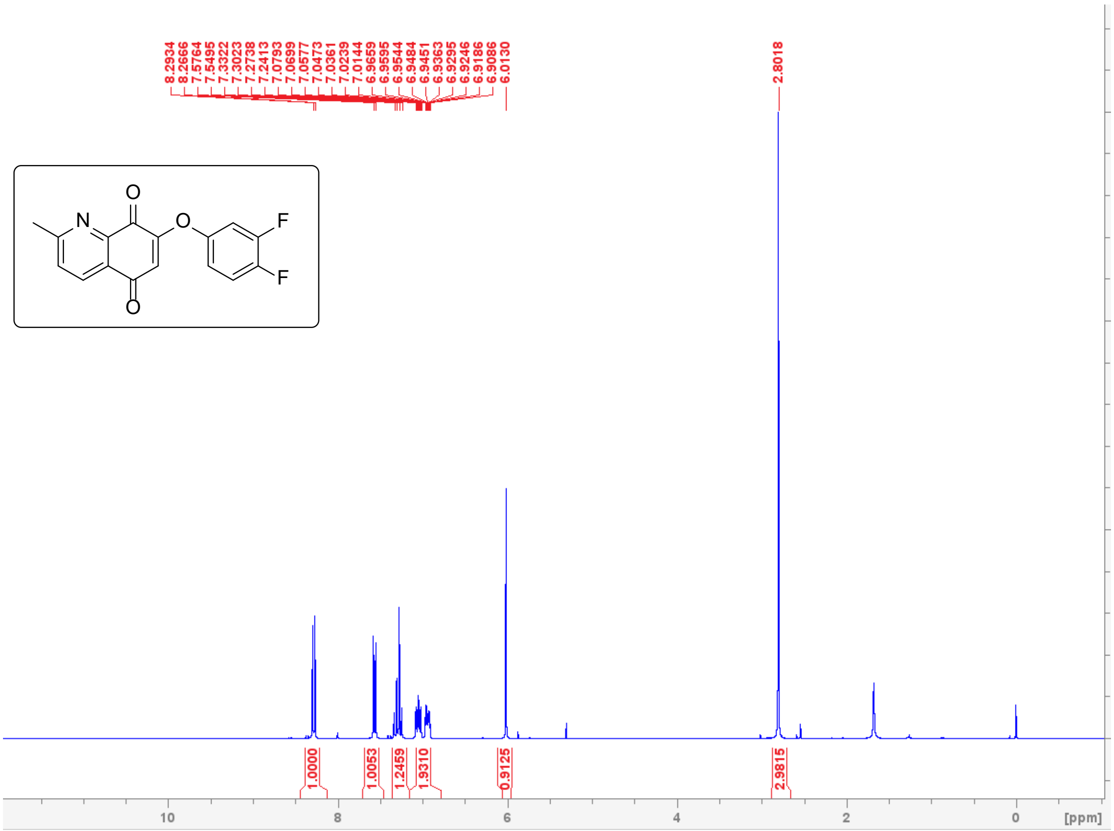

Compound 9

^{13}C NMR

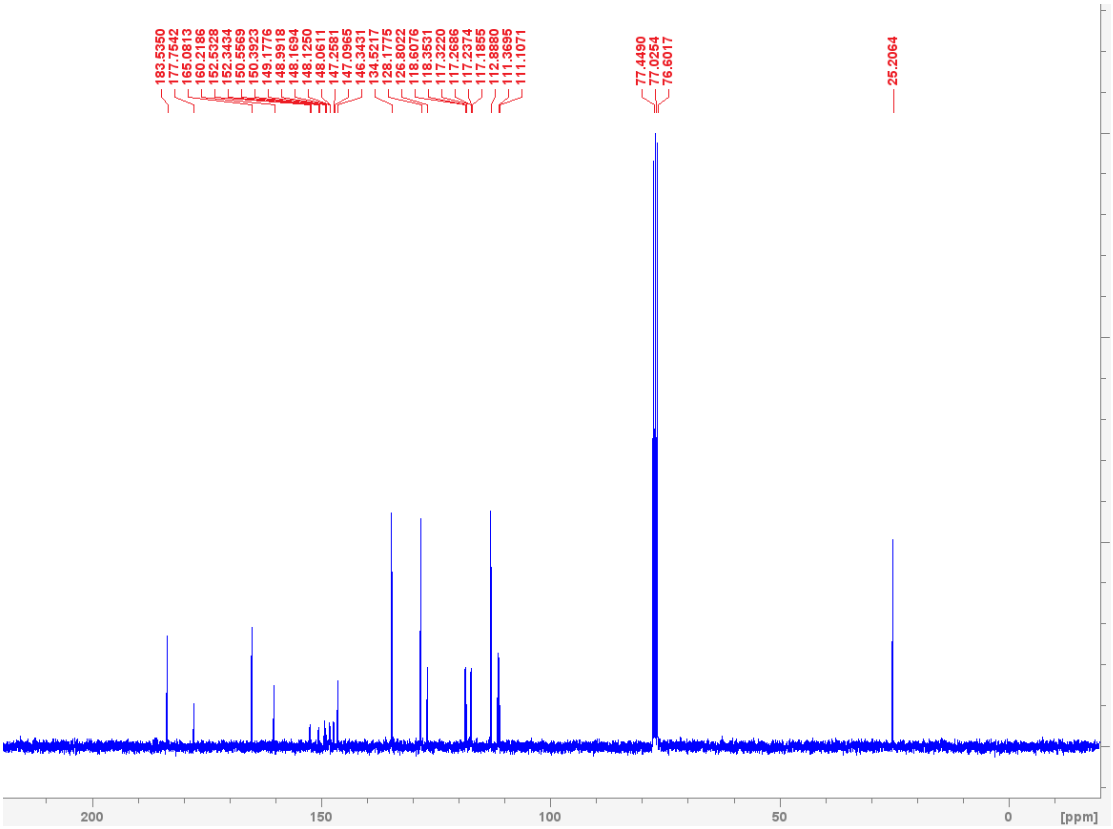

Compound 10

^{1}H NMR

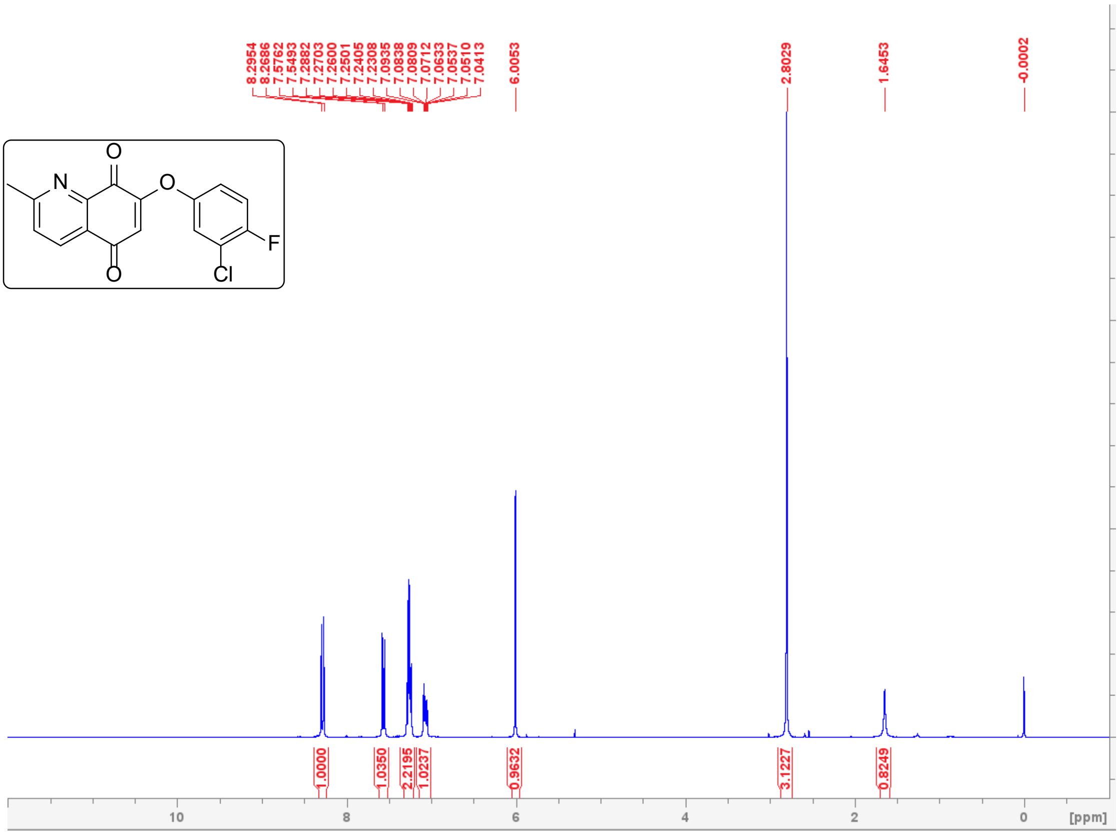

Compound 10

^{13}C NMR

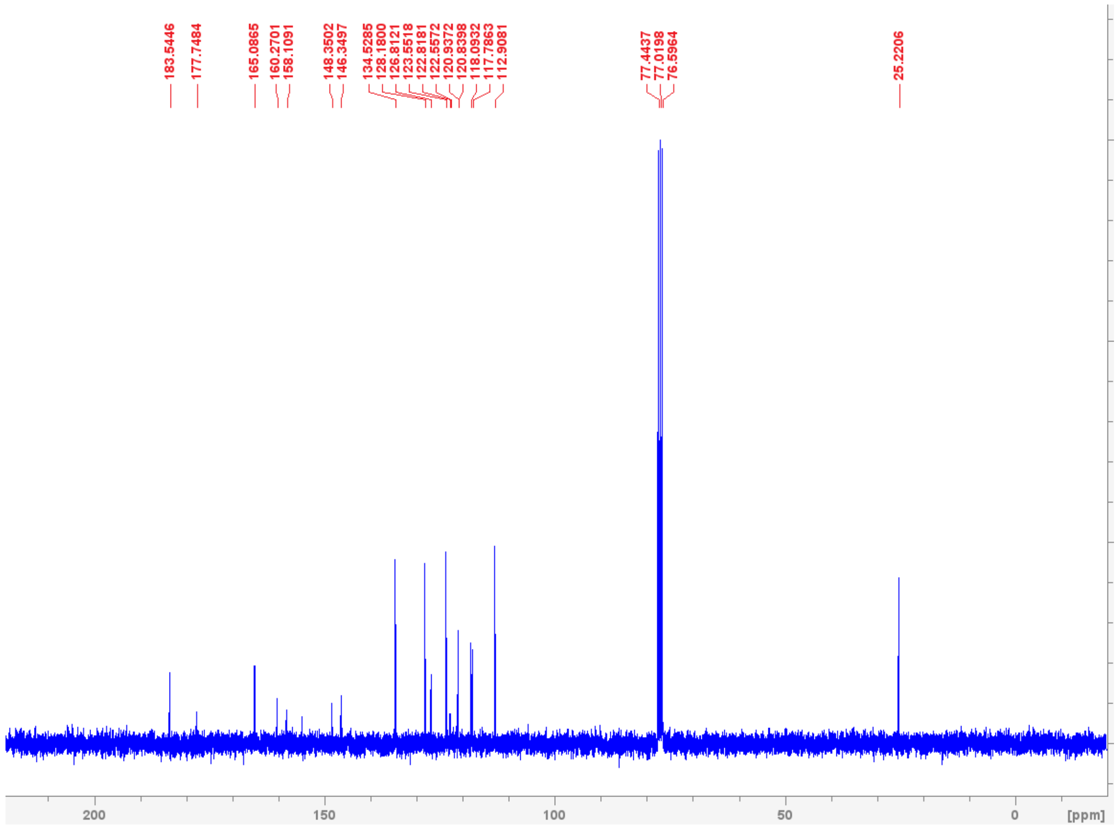

Compound 11

^{1}H NMR

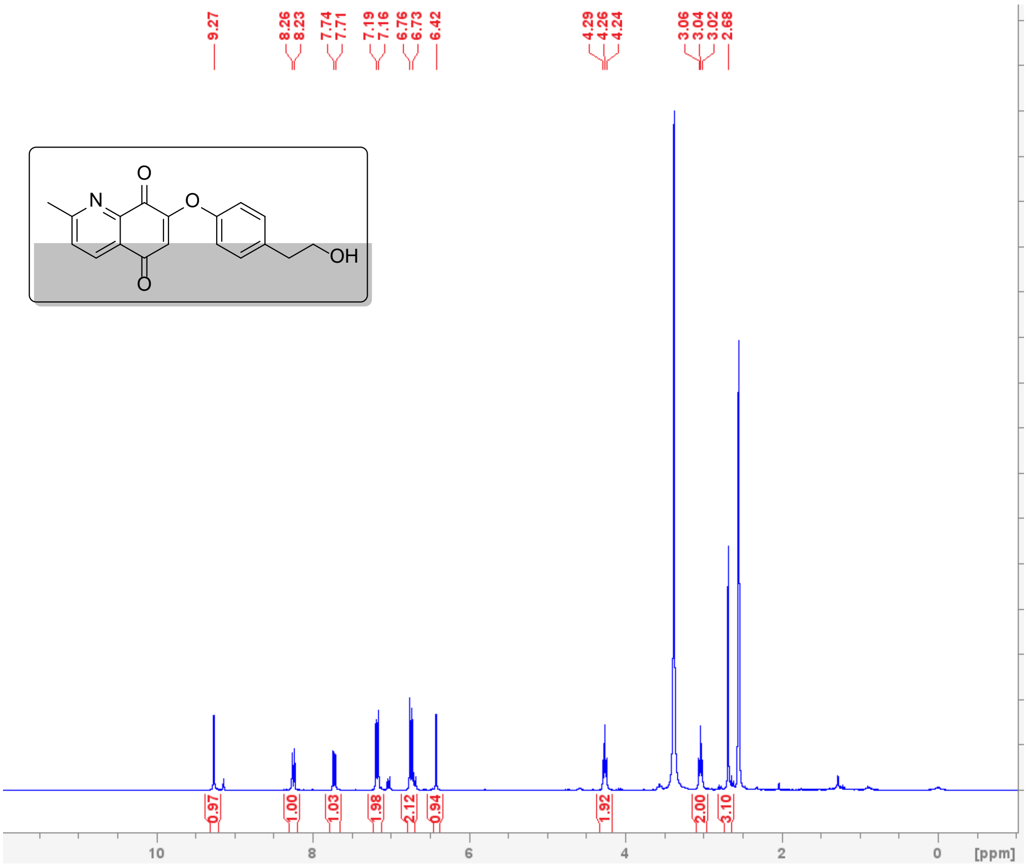

Compound 11

^{13}C NMR

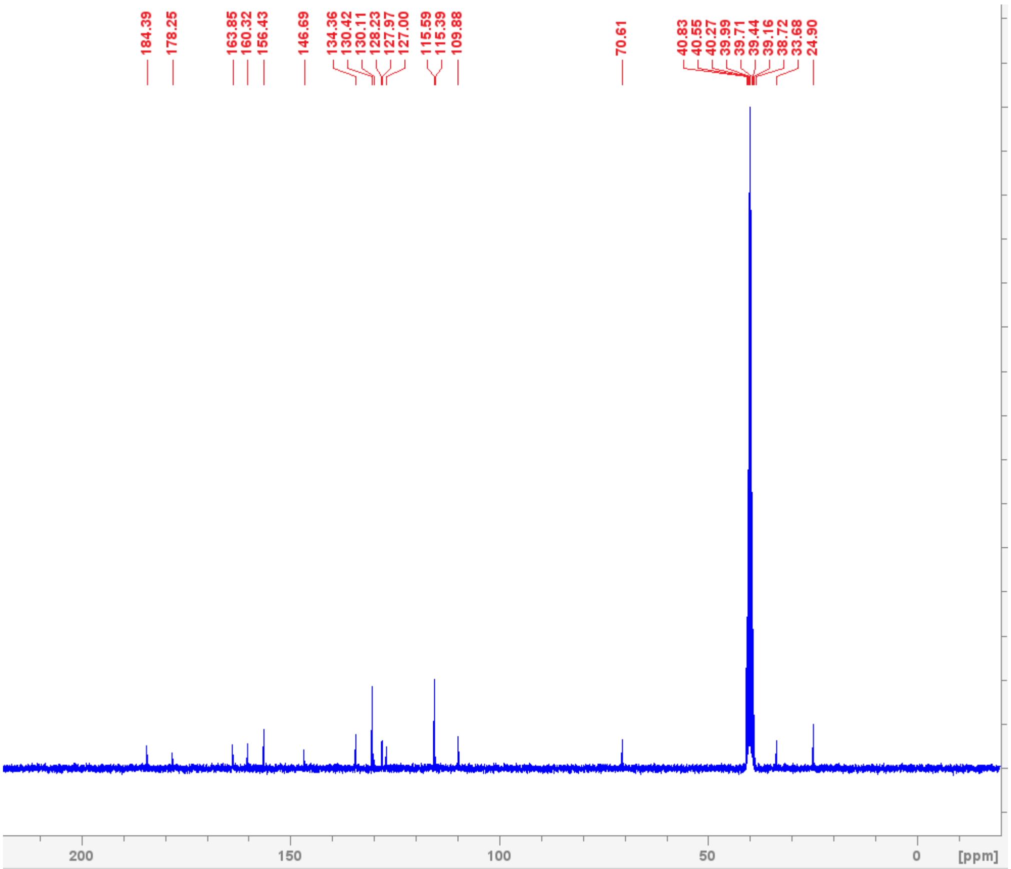

Compound 12

^{1}H NMR

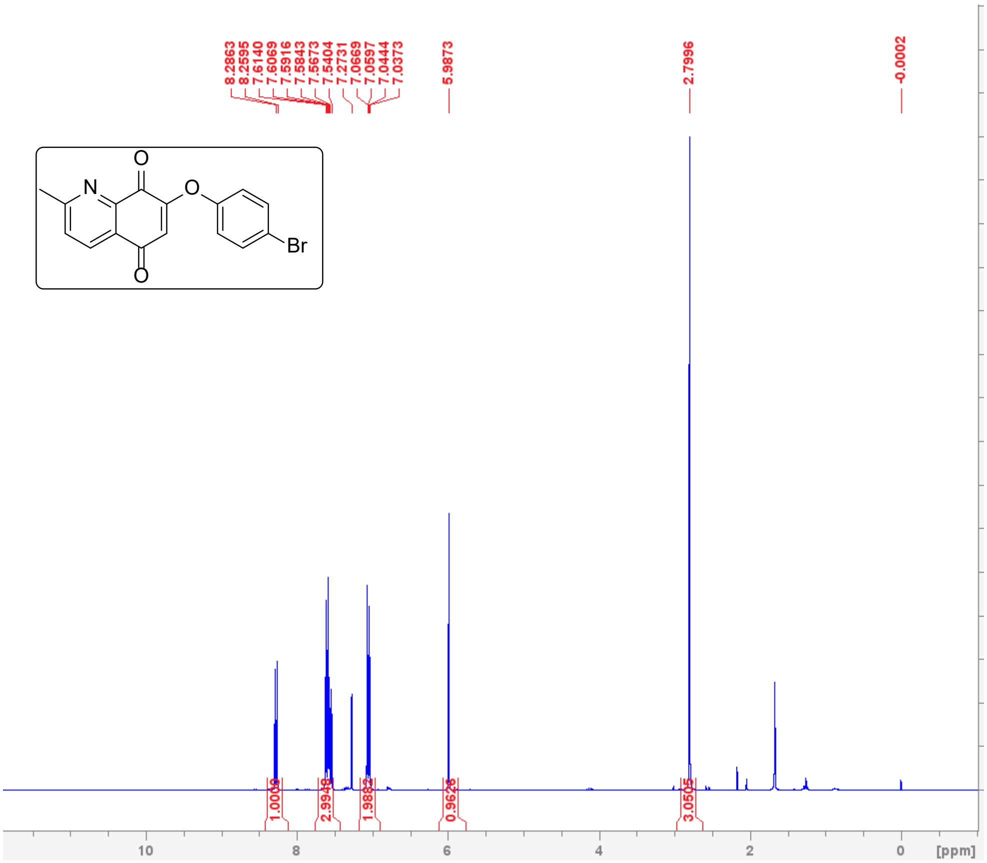

Compound 12

^{13}C NMR

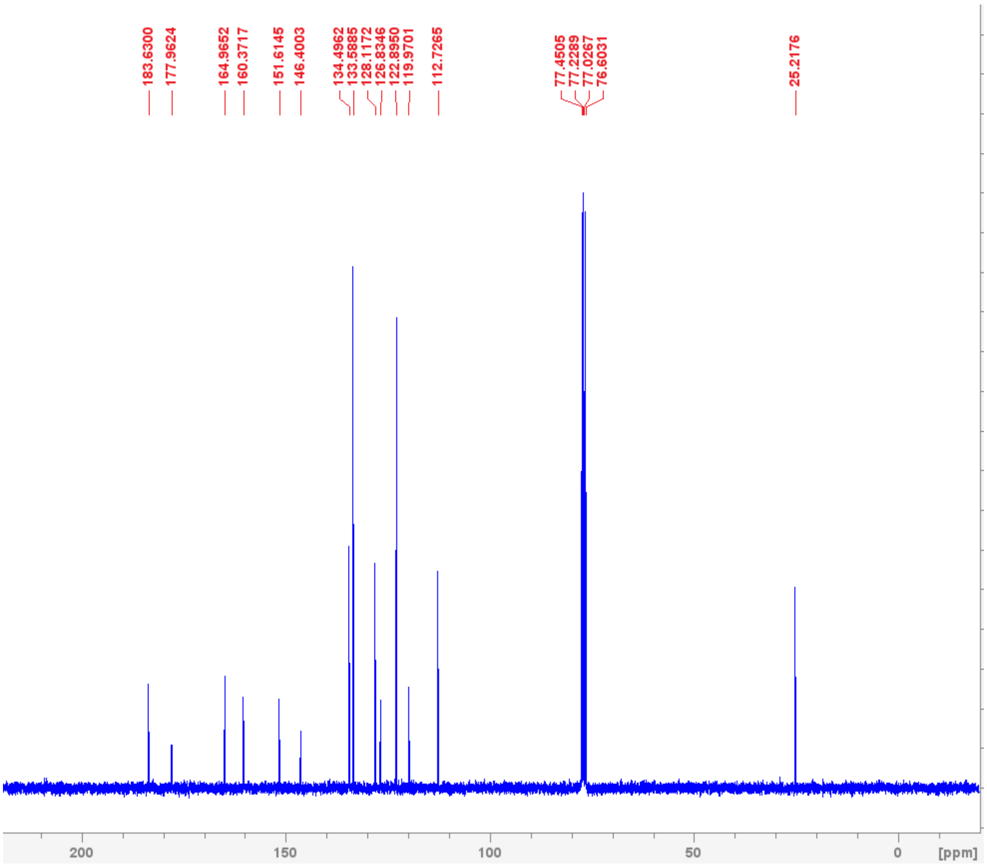

Compound 13

^{1}H NMR

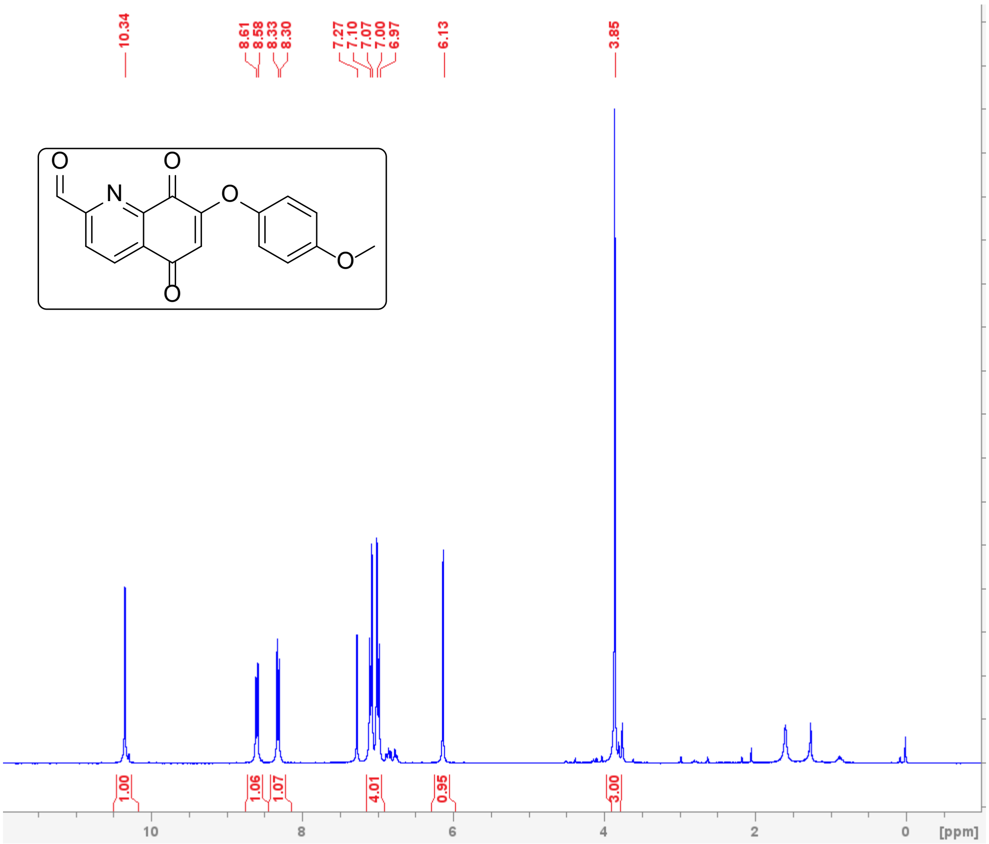

Compound 13

^{13}C NMR

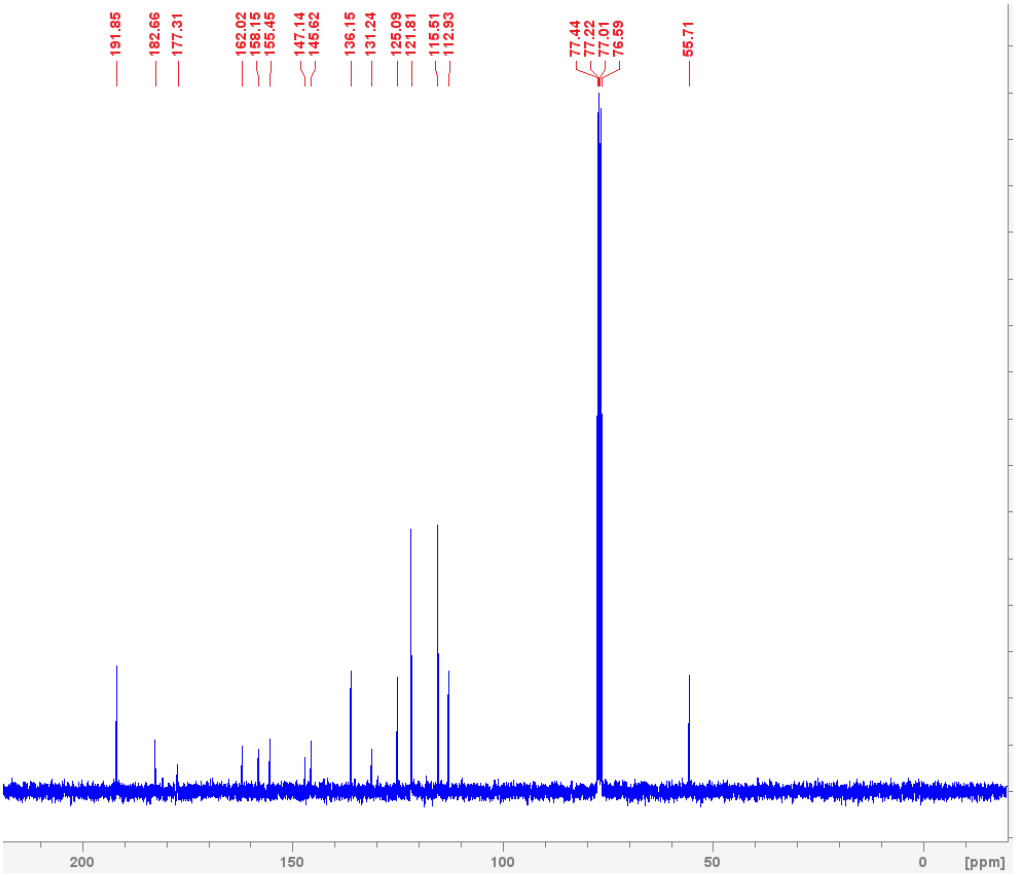

Compound 14

^{1}H NMR

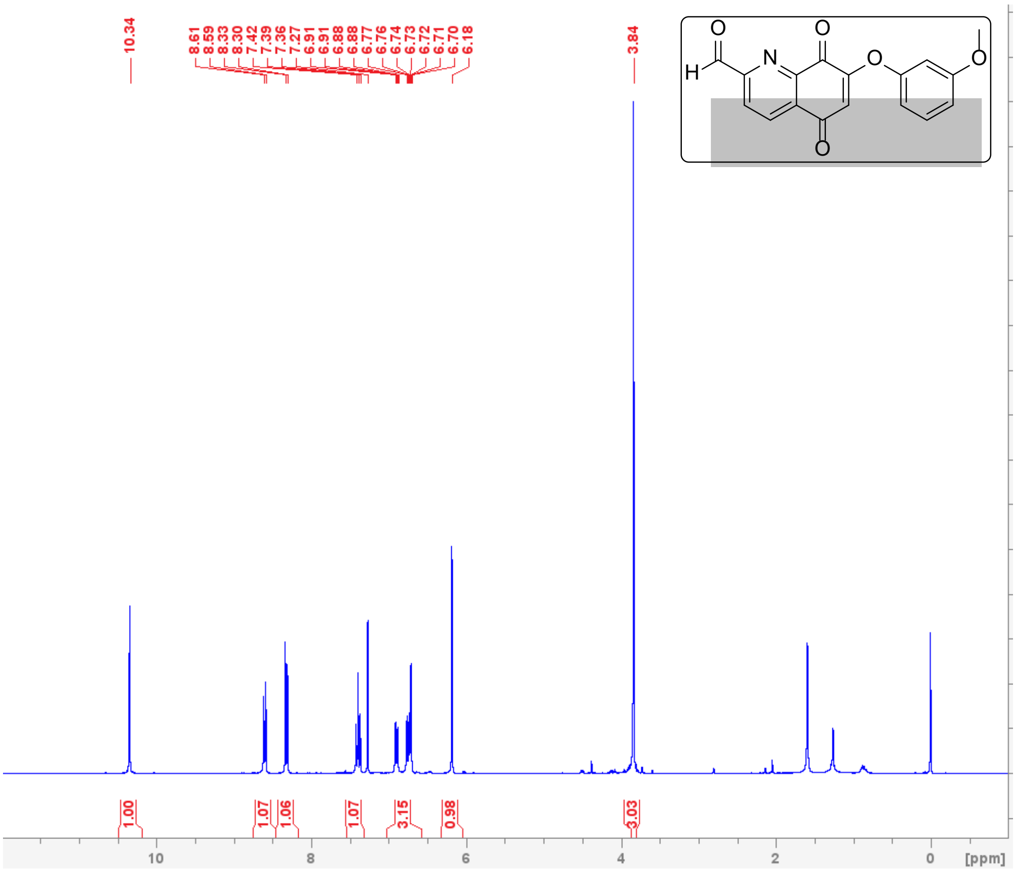

Compound 14

^{13}C NMR

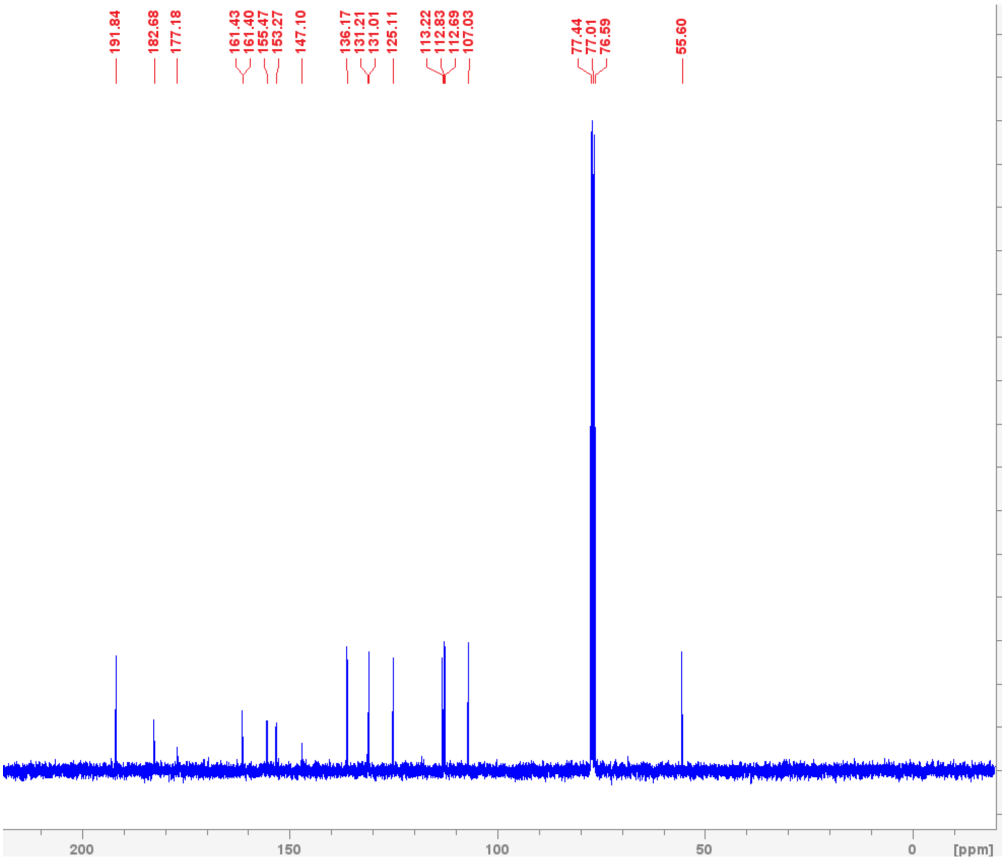

Compound 15

^{1}H NMR

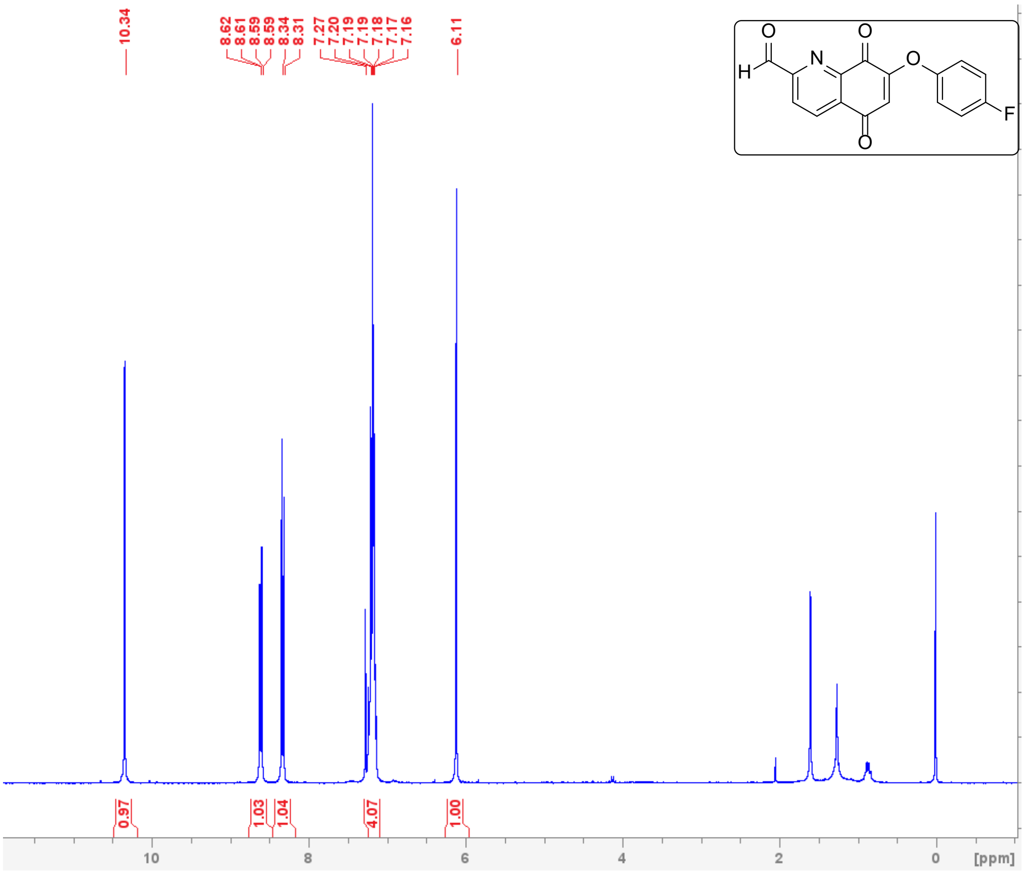

Compound 15

^{13}C NMR

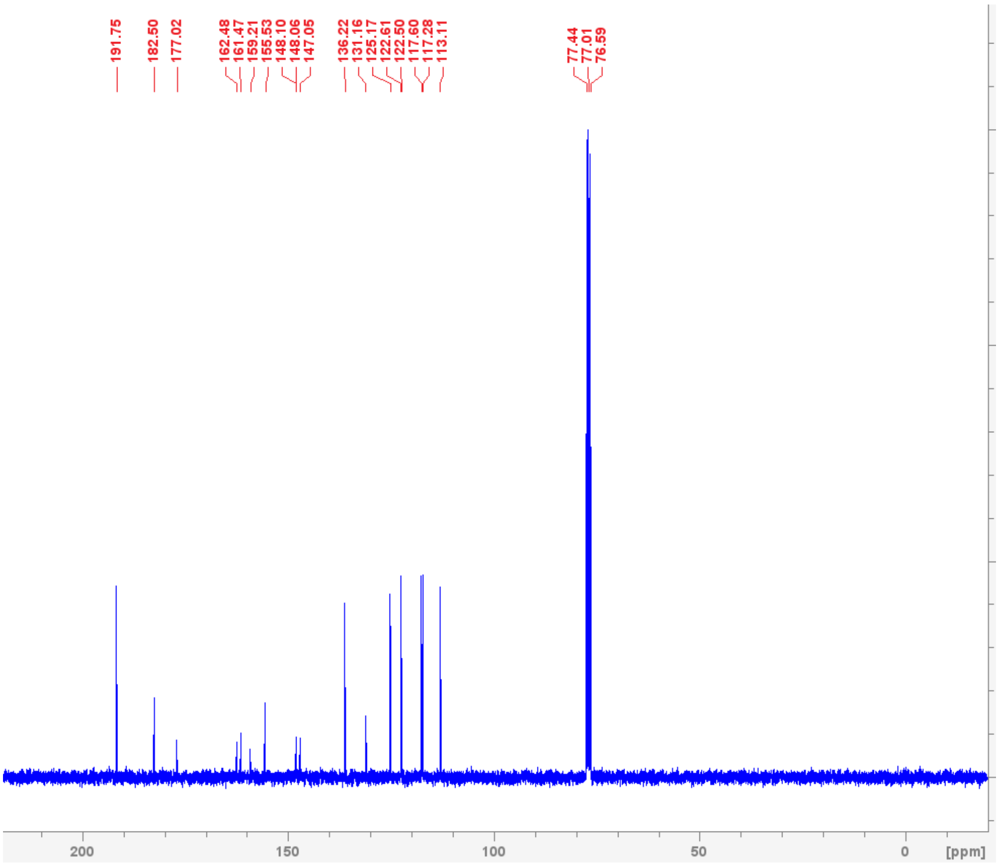

Compound 17

^{1}H NMR

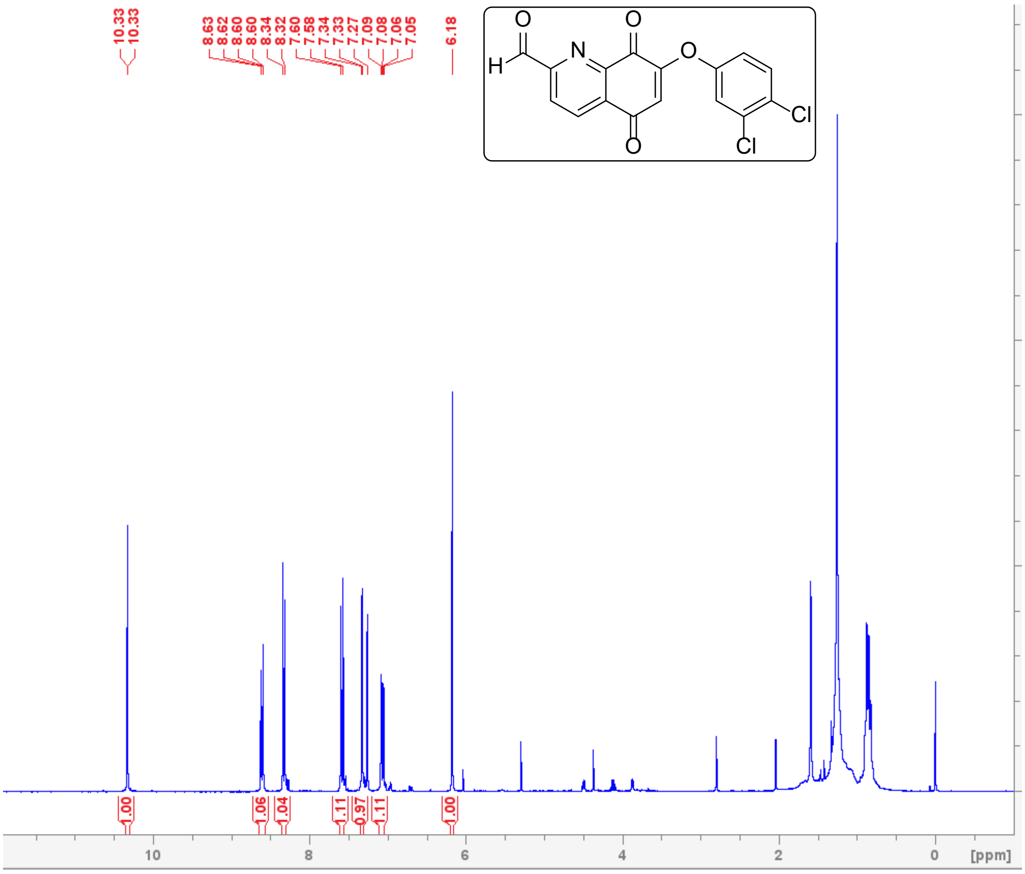

Compound 17

^{13}C NMR

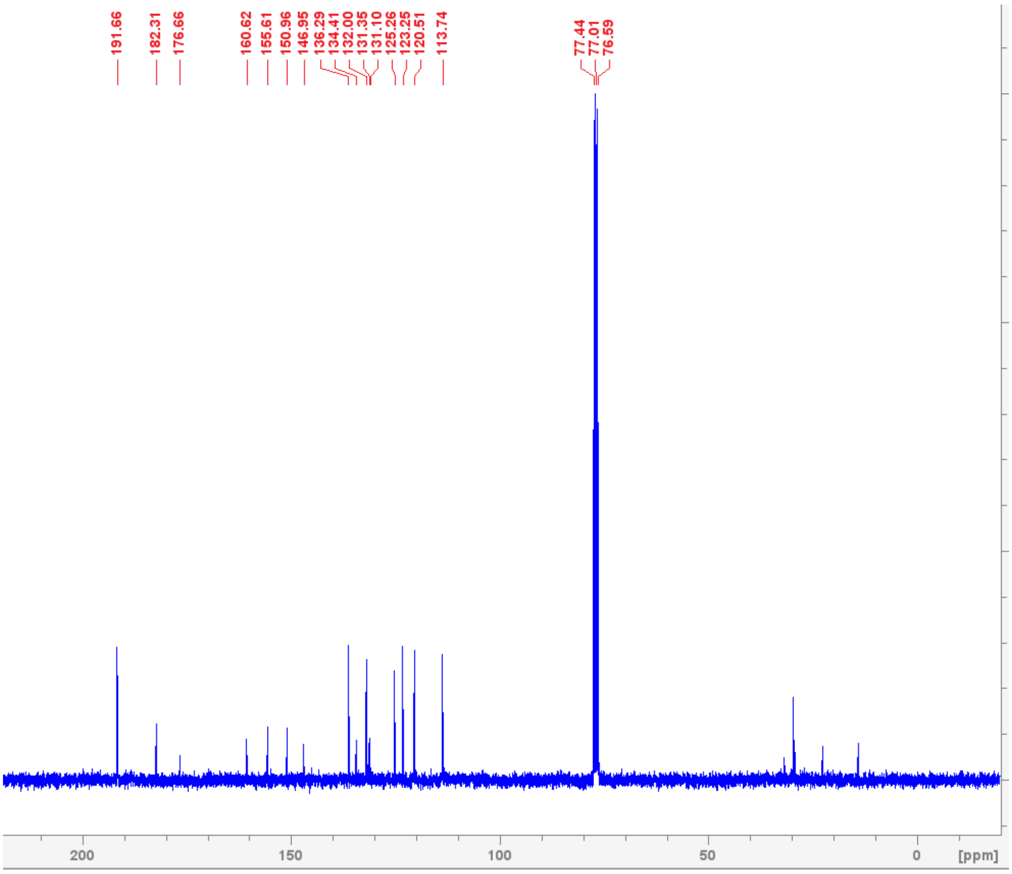

Compound 18

^{1}H NMR

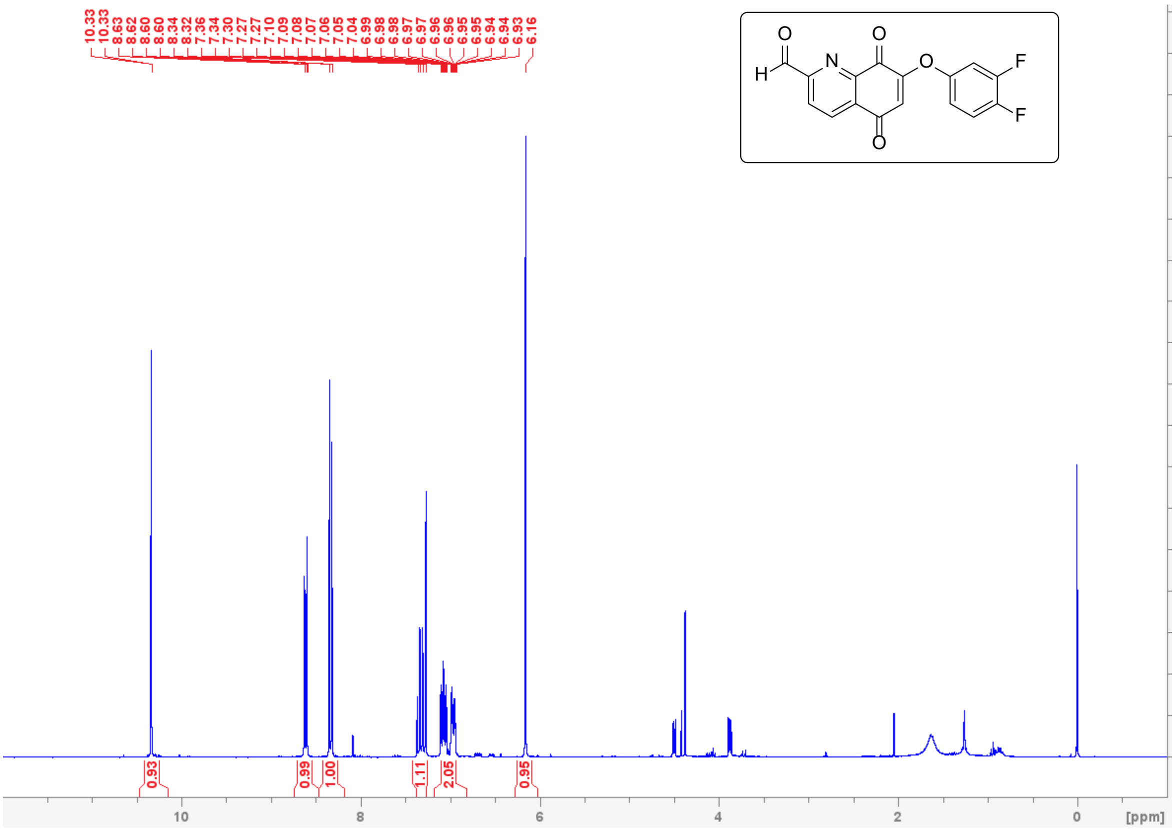

Compound 18

^{13}C NMR

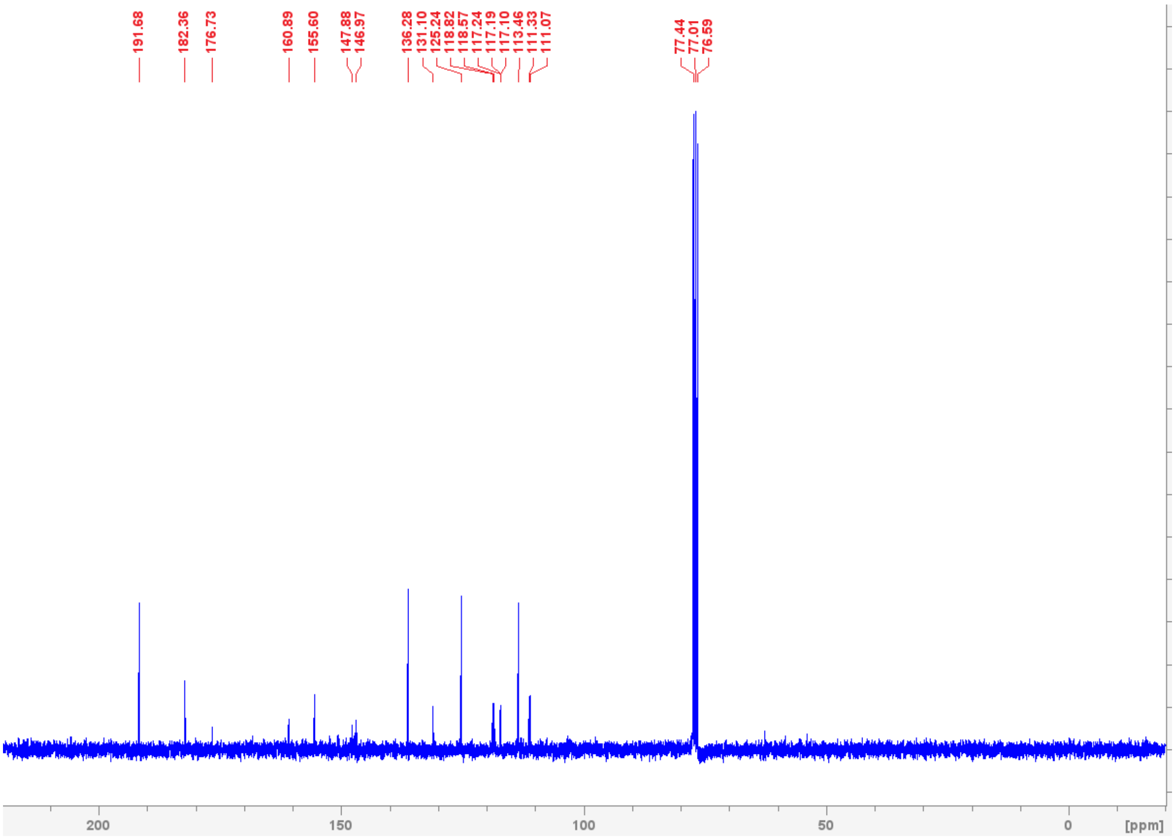

Compound 19

^{1}H NMR

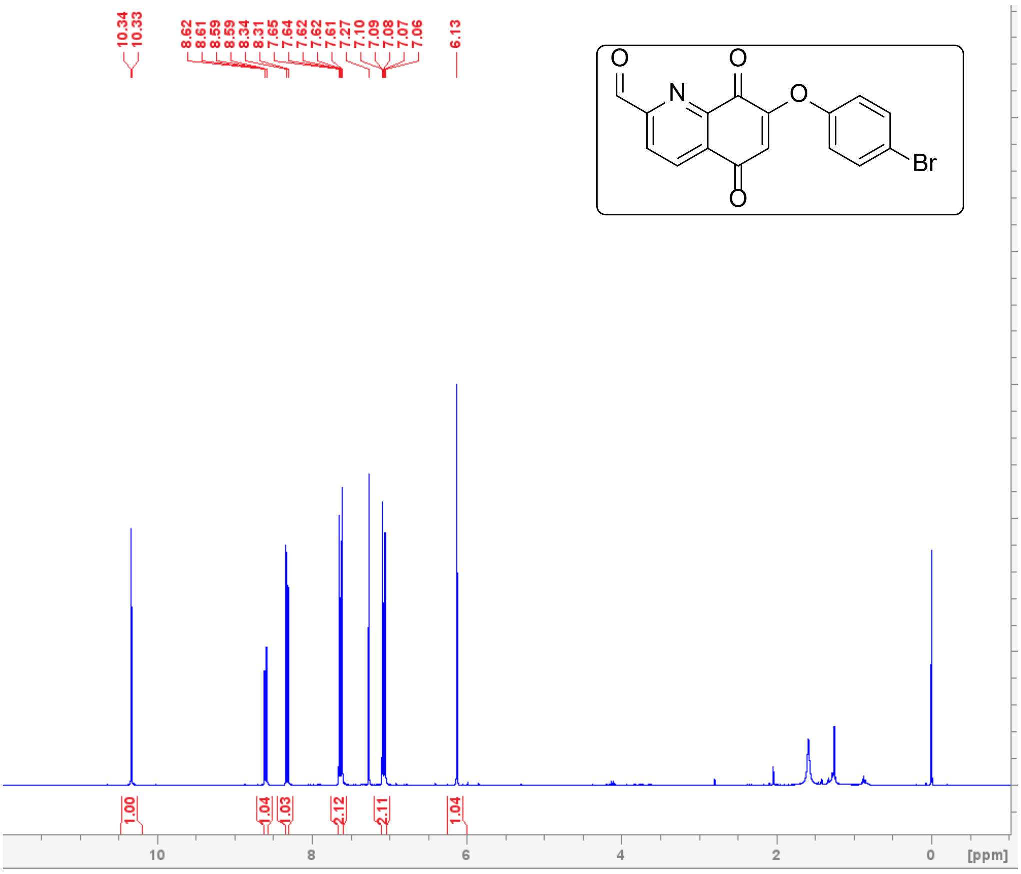

Compound 19

^{13}C NMR

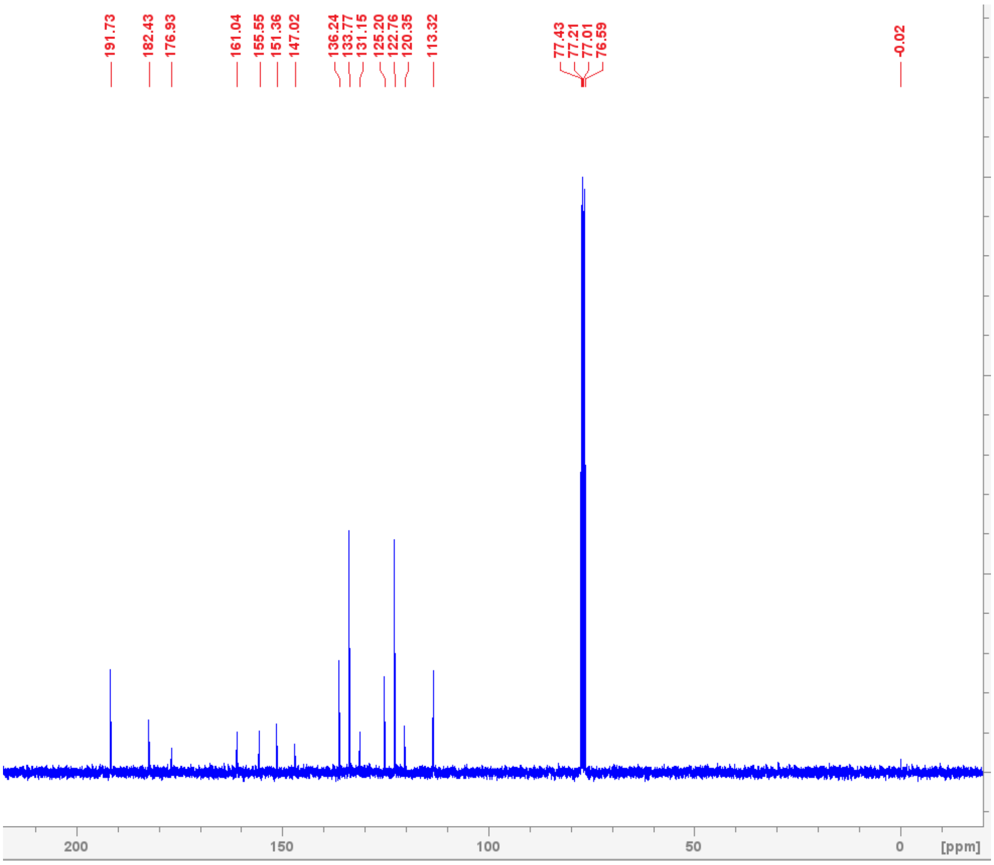

Compound 20

^{1}H NMR

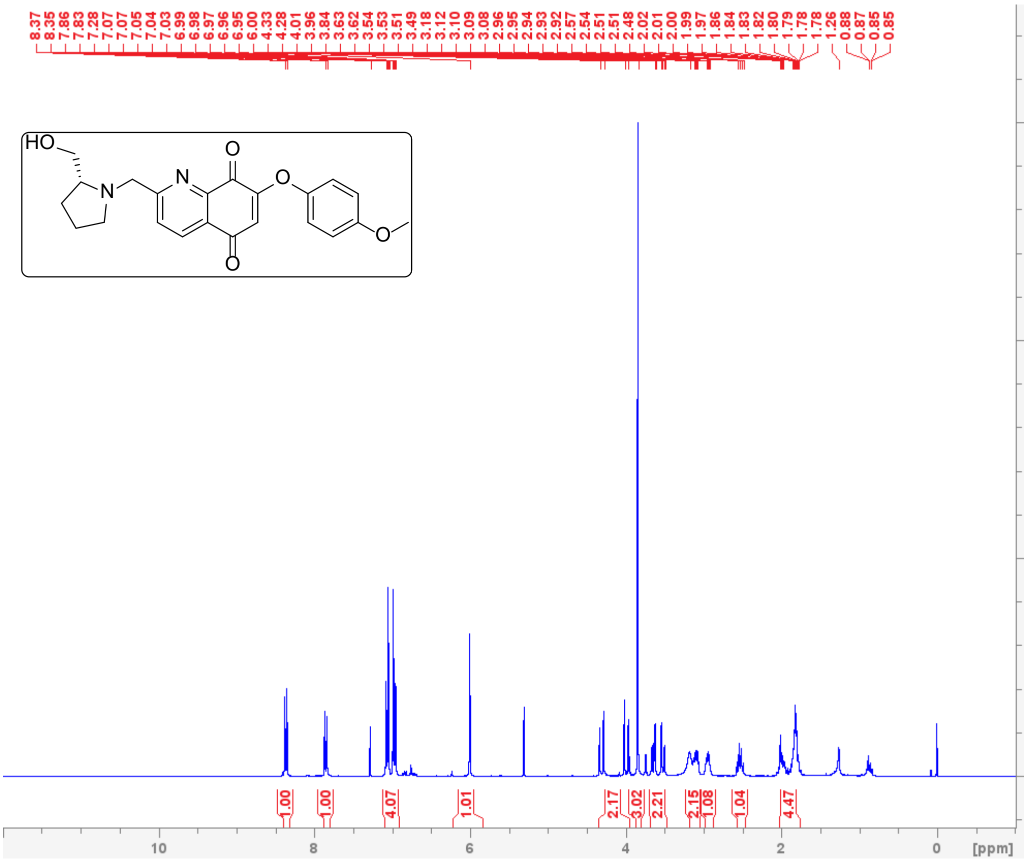

Compound 20

^{13}C NMR

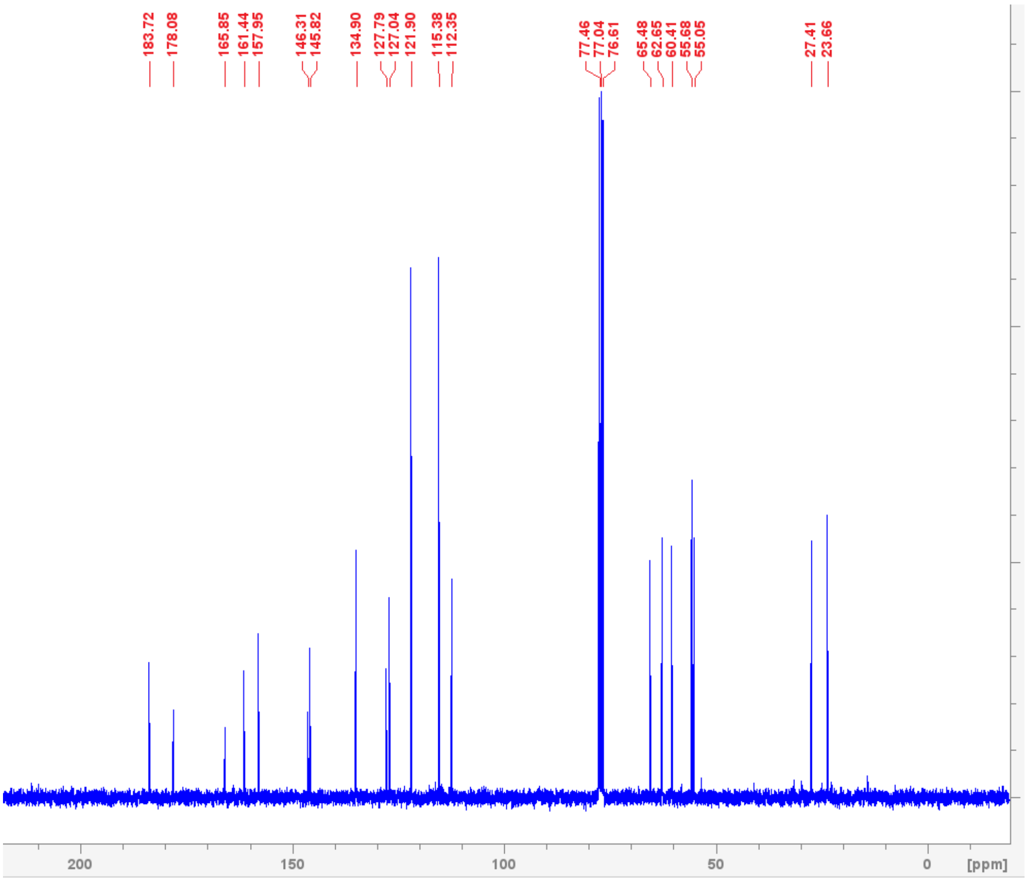

Compound 21

^{1}H NMR

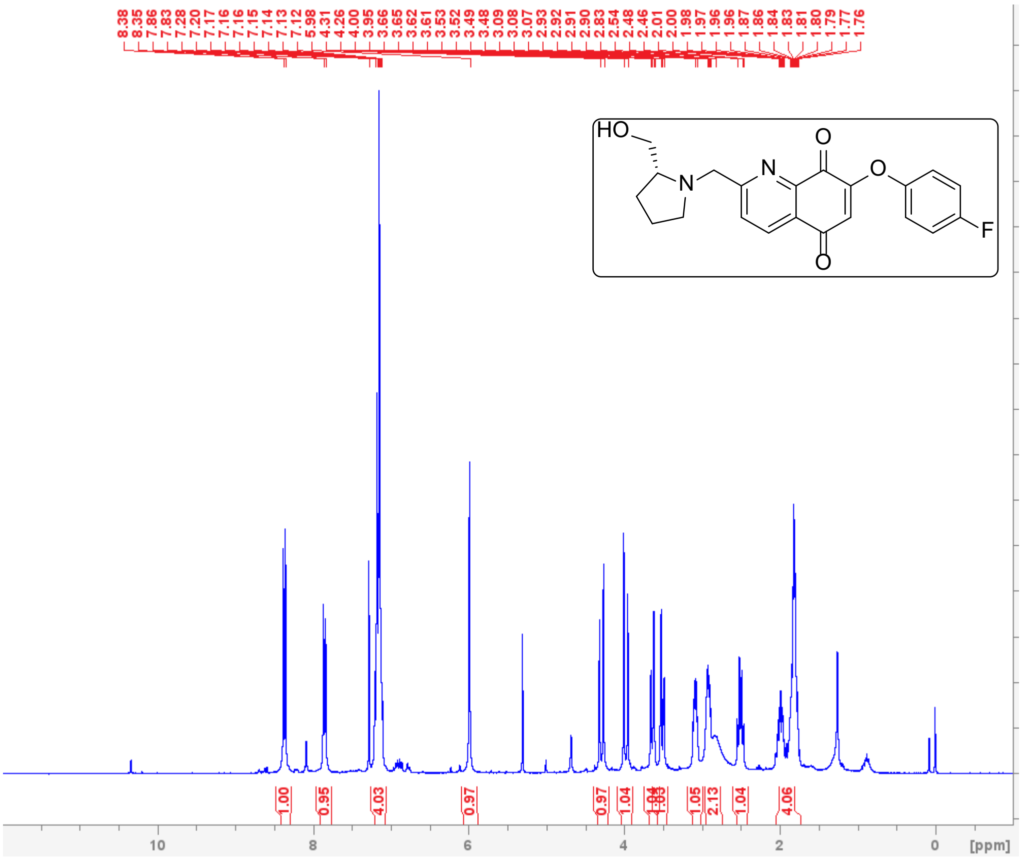

Compound 21

^{13}C NMR

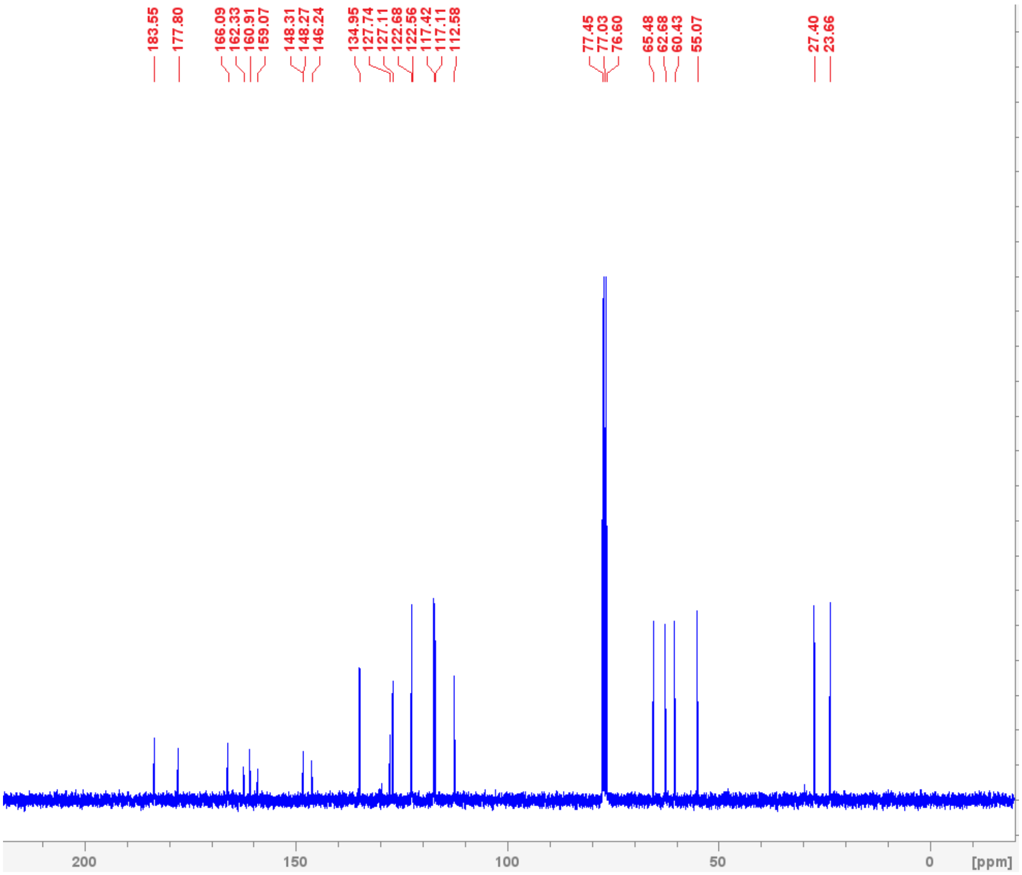

S1.2 HPLC purity of 4 and 20

Instrument: Waters Acquity I-Class

Chromatographic column: Waters Acquity peptide BEH C18 Column, 130Å, 1.7 µm, 2.1 mm X 100 mm

Mobile phase: Water (+0.1% formic acid): Acetonitrile (+0.1% formic acid)

Gradient: 0 mins 90:10; 1 mins 90:10; 12.5mins 10:90: 12.6 mins 90 :10 ; 14 mins 90 :10

Detection wavelength: 250 nm

Detection temperature: 20 °C

Injection volume: 10 µL

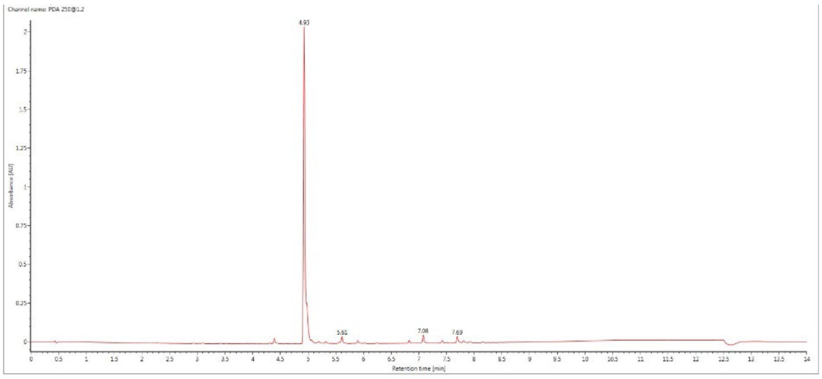

Figure S1 HPLC chromatogram of compound 4

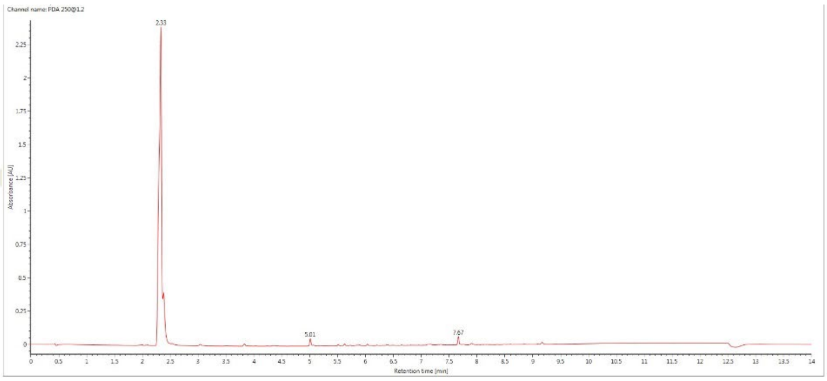

Figure S2 HPLC chromatograms of compound 20

Table S1 *Peak purity of HPLC chromatogram for compounds **4** and **20***

Peak	Retention time [mins]	Peak area [%]
**4**	4.93	98.0
**20**	2.33	96.1

S2 Sphingosine kinase inhibitory assay data

S2.1 Assay Validation and standards (Figure S3-S4; Table S2)

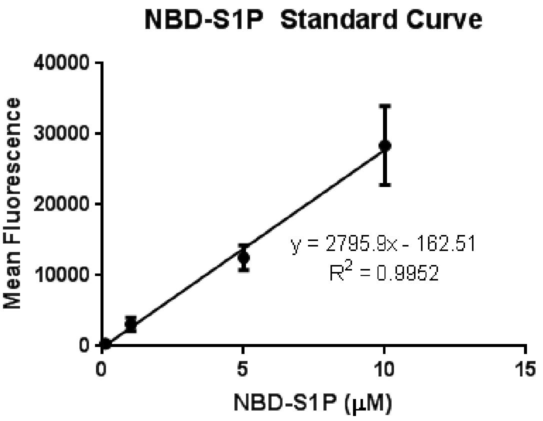

Figure S3 NBD-S1P Fluorescence Standard Curve

Validation with known inhibitors

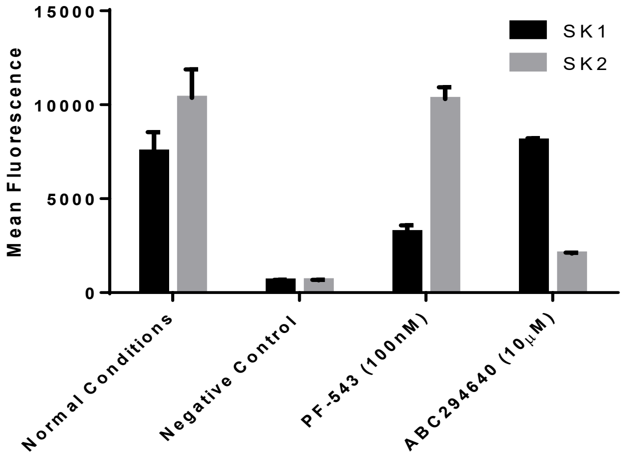

Figure S4 Assay validation and standards

Table S2 SK inhibition assay validation with control compounds PF-543 and ABC284640

Measurements of fluorescence intensity with excitation at 485 nm and emission at 535 nm								
	Spk1					Spk2		
	Average	Normalised	% Inhibition			Average	Normalised	% Inhibition
**Normal Conditions**	7501.5	6857	100.0			10373.5	9723	100.0
**Negative Control (No ATP)**	644.5	0.0	0.0			650.5	0.0	0.0
**PF-543 (100nM)**	3219.0	2574.5	62.5			10309	9658.5	0.7
**ABC294640 (10uM)**	8089.0	7444.5	-8.6			6920.5	6270.0	35.5

S2.2 SK inhibition assay data for 4-21 (*Table S3-S5*)

Table S3 SK inhibition assay data for 5-7, 11 and 20

Fluorescence intensity measurement (excitation at 485 nm / emission at 535 nm)										
		**Spk1**					**Spk2**			
		**A**	**B**	**Average**	**SD**		**A**	**B**	**Average**	**SD**
**Pos**	Measured	6765.0	8238.0				9305.0	11442.0		
	Normalised	6148.0	7566.0	6857.0			8680.0	10766.0	9723.0	
	%	0.0	0.0	0.0			0.0	0.0	**0.0**	
**Neg**	Measured	617.0	672.0	644.5			625.0	676.0		
	Normalised	0.0	0.0	0.0			0.0	0.0	0.0	
	%	100.0	100.0	**100.0**			100.0	100.0	**100.0**	
**5**	Measured	4440.0	5096.0				4953.0	6133.0		
	Normalised	3823.0	4424.0	4123.5			4328.0	5457.0	4892.5	
	%	37.8	41.5	**39.9**	2.6		50.1	49.3	**49.7**	0.6
**6**	Measured	3479.0	4092.0				4219.0	4938.0		
	Normalised	2862.0	3420.0	3141.0			3594.0	4262.0	3928.0	
	%	53.4	54.8	**54.2**	1.0		58.6	60.4	**59.6**	1.3
**7**	Measured	3442.0	4059.0				4650.0	5288.0		
	Normalised	2825.0	3387.0	3106.0			4025.0	4612.0	4318.5	
	%	54.1	55.2	**54.7**	0.8		53.6	57.2	**55.6**	2.5
**11**	Measured	5686.0	6715.0				6576.0	7733.0		
	Normalised	5069.0	6043.0	5556.0			5951.0	7057.0	6504.0	
	%	17.6	20.1	**19.0**	1.8		31.4	34.5	**33.1**	2.1
**20**	Measured	2974.0	3371.0				2256.0	2626.0		
	Normalised	2357.0	2699.0	2528.0			1631.0	1950.0	1790.5	
	%	61.7	64.3	**63.1**	1.9		81.2	81.9	**81.6**	0.5

Table S4 SK inhibition assay data for 8-10, 14-15 and 18-19

Fluorescence intensity measurements (excitation at 485 nm / emission at 535 nm)										
		**Spk1**					**Spk2**			
		**A**	**B**	**Average**	**SD**		**A**	**B**	**Average**	**SD**
**Pos**	Measured	6247.0	7910.0	7078.5			10133.0	12679.0	11406.0	
	Normalised	6061.0	7674.0	6857.5			9934.0	12477.0	11205.5	
	%	0.0	0.0	**0.0**			0.0	0.0	**0.0**	
**Neg**	Measured	186.0	236.0	221.0			199.0	202.0	200.5	
	Normalised	0.0	0.0	0.0			0.0	0.0	0.0	
	%	100.0	100.0	**100.0**			100.0	100.0	**100.0**	
**8**	Measured	5219.0	5446.0				6556.0	7789.0		
	Normalised	5033.0	5210.0	5121.5			6357.0	7587.0	6972.0	
	%	17.0	32.1	**25.3**	10.7		36.0	39.2	**37.8**	2.3
**9**	Measured	5374.0	5982.0				7414.0	9747.0		
	Normalised	5188.0	5746.0	5467.0			7215.0	9545.0	8380.0	
	%	14.4	25.1	**20.3**	7.6		27.4	23.5	**25.2**	2.7

**10**	Measured	6713.0	8406.0				10600.0	12642.0		
	Normalised	6527.0	8170.0	7348.5			10401.0	12440.0	11420.5	
	%	-7.7	-6.5	**-7.2**	0.9		-4.7	0.3	**-1.9**	3.5
**14**	Measured	6771.0	8284.0				10303.0	12425.0		
	Normalised	6585.0	8048.0	7316.5			10104.0	12223.0	11163.5	
	%	-8.6	-4.9	**-6.7**	2.7		-1.7	2.0	**0.4**	2.6
**15**	Measured	5870.0	6740.0				8331.0	9526.0		
	Normalised	5684.0	6504.0	6094.0			8132.0	9324.0	8728.0	
	%	6.2	15.2	**11.1**	6.4		18.1	25.3	**22.1**	5.0
**18**	Measured	7868.0	9381.0				12100.0	14393.0		
	Normalised	7682.0	9145.0	8413.5			11901.0	14191.0	13046.0	
	%	-26.7	-19.2	**-22.7**	5.4		-19.8	-13.7	**-16.4**	4.3
**19**	Measured	6494.0	7773.0				9461.0	12637.0		
	Normalised	6308.0	7537.0	6922.5			9262.0	12435.0	10848.5	
	%	-4.1	1.8	**-0.9**	4.1		6.8	0.3	**3.2**	4.5

Table S5 **SK inhibition assay data for 4, 13 and 21**

**Fluorescence intensity measurements (excitation at 485 nm / emission at 535 nm)**										
		**Spk1**					**Spk2**			
		**A**	**B**	**Average**	**SD**		**A**	**B**	**Average**	**SD**
**Pos**	Measured	4133.0	5243.0	4688.0			9235.0	10995.0	10115.0	
	Normalised	1933.0	2485.0	2209.0			7649.0	9342.0	8495.5	
	%	0.0	0.0	**0.0**			0.0	0.0	**0.0**	
**Neg**	Measured	2200.0	2758.0	2479.0			1586.0	1653.0	1619.5	
	Normalised	0.0	0.0	0.0			0.0	0.0	0.0	
	%	100.0	100.0	**100.0**			100	100	**100**	
**4**	Measured	3002.0	3316.0				6368.0	7601.0		
	Normalised	802.0	558.0	680.0			4782.0	5948.0	5365.0	
	%	58.5	77.5	**69.2**	13.5		37.5	36.3	**36.8**	0.8
**13**	Measured	4050.0	4682.0				6321.0	7160.0		
	Normalised	1850.0	1924.0	1887.0			4735.0	5507.0	5121.0	
	%	4.3	22.6	**14.6**	12.9		38.1	41.1	**39.7**	2.1
**21**	Measured	2935.0	3360.0				5946.0	6939.0		
	Normalised	735.0	602.0	668.5			4360.0	5286.0	4823.0	
	%	62.0	75.8	**69.7**	9.8		43.0	43.4	**43.2**	0.3

S3. Calculated Data

S3.1 Table S6 In silico SphK1 Docking Score and Lipophilicity

Compound	Score[a]	CLogP consensus (and WLOGP)[b]
**PF-543**	-109.2	4.24 (4.75)
**4**		2.34 (2.74)
**5**		2.33 (2.74)
**6**	-56.5	2.66 (3.29)
**7**		2.12 (2.60)
**8**		3.41 (4.04)
**9**		2.98 (3.85)
**10**		3.18 (3.94)
**11**		2.18 (2.27)
**12**		2.97 (3.49)
**13**		1.68 (2.24)
**14**		1.68 (2.24)
**15**		2.00 (2.80)
**16 (CN)**		N/A
**17**		2.73 (3.54)
**18**		2.30 (3.35)
**19**		2.30 (3.00)
**20**		2.02 (1.86)
**21**	-82.7	2.32 (2.41)

[*a*] Docking Scores were calculated using CLC Drug Discovery Workbench 3.0 which utilises the docking scoring function $PLANTS_{PLP}$ using PDB 4V24 as the starting point.

[*b*] Lipophilicity of compounds **4**-**21** computed via Swiss ADME.[6]

S3.2 Figure S5 *In silico ADME parameters of* **4**.

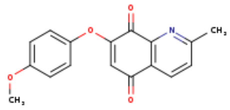

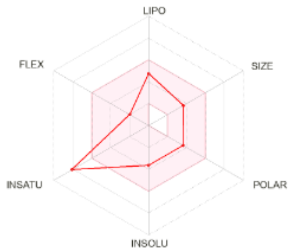

SMILES COc1ccc(cc1)OC1=CC(=O)c2c(C1=O)nc(cc2)C

Physicochemical Properties	
Formula	C17H13NO4
Molecular weight	295.29 g/mol
Num. heavy atoms	22
Num. arom. heavy atoms	12
Fraction Csp3	0.12
Num. rotatable bonds	3
Num. H-bond acceptors	5
Num. H-bond donors	0
Molar Refractivity	79.51
TPSA	65.49 Å²
**Lipophilicity**	
Log $P_{o/w}$ (iLOGP)	2.29
Log $P_{o/w}$ (XLOGP3)	2.82
Log $P_{o/w}$ (WLOGP)	2.74
Log $P_{o/w}$ (MLOGP)	0.51
Log $P_{o/w}$ (SILICOS-IT)	3.32
Consensus Log $P_{o/w}$	2.34

Water Solubility	
Log *S* (ESOL)	-3.65
Solubility	6.56e-02 mg/ml ; 2.22e-04 mol/l
Class	Soluble
Log *S* (Ali)	-3.85
Solubility	4.14e-02 mg/ml ; 1.40e-04 mol/l
Class	Soluble
Log *S* (SILICOS-IT)	-5.43
Solubility	1.11e-03 mg/ml ; 3.75e-06 mol/l
Class	Moderately soluble
**Pharmacokinetics**	
GI absorption	High
BBB permeant	Yes
P-gp substrate	No
CYP1A2 inhibitor	Yes
CYP2C19 inhibitor	Yes
CYP2C9 inhibitor	Yes
CYP2D6 inhibitor	No
CYP3A4 inhibitor	Yes
Log K_p (skin permeation)	-6.10 cm/s
**Druglikeness**	
Lipinski	Yes; 0 violation
Ghose	Yes
Veber	Yes
Egan	Yes
Muegge	Yes
Bioavailability Score	0.85
**Medicinal Chemistry**	
PAINS	1 alert: quinone_A
Brenk	0 alert
Leadlikeness	Yes
Synthetic accessibility	3.00

S3.3 Figure S6 *In silico ADME parameters of* **20**.

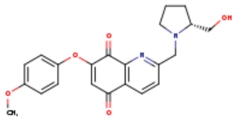

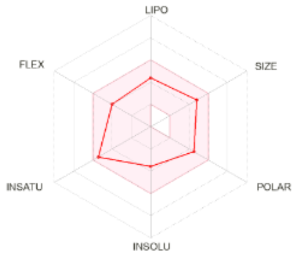

SMILES OC[C@H]1CCCN1Cc1ccc2c(n1)C(=O)C(=CC2=O)Oc1ccc(cc1)OC

Physicochemical Properties	
Formula	C22H22N2O5
Molecular weight	394.42 g/mol
Num. heavy atoms	29
Num. arom. heavy atoms	12
Fraction Csp3	0.32
Num. rotatable bonds	6
Num. H-bond acceptors	7
Num. H-bond donors	1
Molar Refractivity	109.40
TPSA	88.96 Å²
**Lipophilicity**	
Log $P_{o/w}$ (iLOGP)	3.02
Log $P_{o/w}$ (XLOGP3)	2.16
Log $P_{o/w}$ (WLOGP)	1.86
Log $P_{o/w}$ (MLOGP)	0.04
Log $P_{o/w}$ (SILICOS-IT)	3.01
Consensus Log $P_{o/w}$	2.02

Water Solubility	
Log *S* (ESOL)	-3.56
Solubility	1.10e-01 mg/ml ; 2.78e-04 mol/l
Class	Soluble
Log *S* (Ali)	-3.66
Solubility	8.61e-02 mg/ml ; 2.18e-04 mol/l
Class	Soluble
Log *S* (SILICOS-IT)	-5.42
Solubility	1.49e-03 mg/ml ; 3.77e-06 mol/l
Class	Moderately soluble
**Pharmacokinetics**	
GI absorption	High
BBB permeant	No
P-gp substrate	Yes
CYP1A2 inhibitor	No
CYP2C19 inhibitor	No
CYP2C9 inhibitor	Yes
CYP2D6 inhibitor	No
CYP3A4 inhibitor	Yes
Log K_p (skin permeation)	-7.17 cm/s
**Druglikeness**	
Lipinski	Yes; 0 violation
Ghose	Yes
Veber	Yes
Egan	Yes
Muegge	Yes
Bioavailability Score	0.55
**Medicinal Chemistry**	
PAINS	1 alert: quinone_A
Brenk	0 alert
Leadlikeness	No; 1 violation: MW>350
Synthetic accessibility	3.92

S4 NCI 60 Cell Line Growth Percent for selected compounds at 10μM

S4.1 Figure S7 NCI One dose data for Compound 4

Developmental Therapeutics Program One Dose Mean Graph	NSC: 815382 / 1	Conc: 1.00E-5 Molar	Test Date: May 20, 2019
	Experiment ID: 1905OS11		Report Date: Oct 04, 2022

Panel/Cell Line	Growth Percent
Leukemia	
CCRF-CEM	18.46
HL-60(TB)	52.05
K-562	43.45
MOLT-4	66.49
RPMI-8226	79.70
SR	51.19
Non-Small Cell Lung Cancer	
A549/ATCC	96.38
EKVX	96.53
HOP-62	89.09
HOP-92	121.75
NCI-H23	65.65
NCI-H322M	101.94
NCI-H460	86.07
NCI-H522	55.15
Colon Cancer	
HCC-2998	102.50
HCT-116	74.28
HCT-15	76.10
HT29	102.19
KM12	81.34
SW-620	94.86
CNS Cancer	
SF-268	79.24
SF-295	96.78
SF-539	89.56
SNB-19	87.75
SNB-75	53.45
U251	96.37
Melanoma	
LOX IMVI	75.55
MALME-3M	128.36
M14	87.32
MDA-MB-435	83.03
SK-MEL-2	99.48
SK-MEL-28	93.52
SK-MEL-5	83.50
UACC-257	93.76
UACC-62	83.59
Ovarian Cancer	
IGROV1	87.77
OVCAR-3	72.35
OVCAR-5	114.47
OVCAR-8	80.74
NCI/ADR-RES	87.50
Renal Cancer	
786-0	109.27
ACHN	51.41
CAKI-1	82.46
RXF 393	88.86
SN12C	88.29
TK-10	111.97
UO-31	92.35
Prostate Cancer	
PC-3	85.19
DU-145	101.61
Breast Cancer	
MCF7	72.41
MDA-MB-231/ATCC	90.08
HS 578T	83.22
BT-549	90.66
T-47D	79.87
MDA-MB-468	86.19
Mean	84.42
Delta	65.96
Range	109.90

Mean Growth Percent - Growth Percent

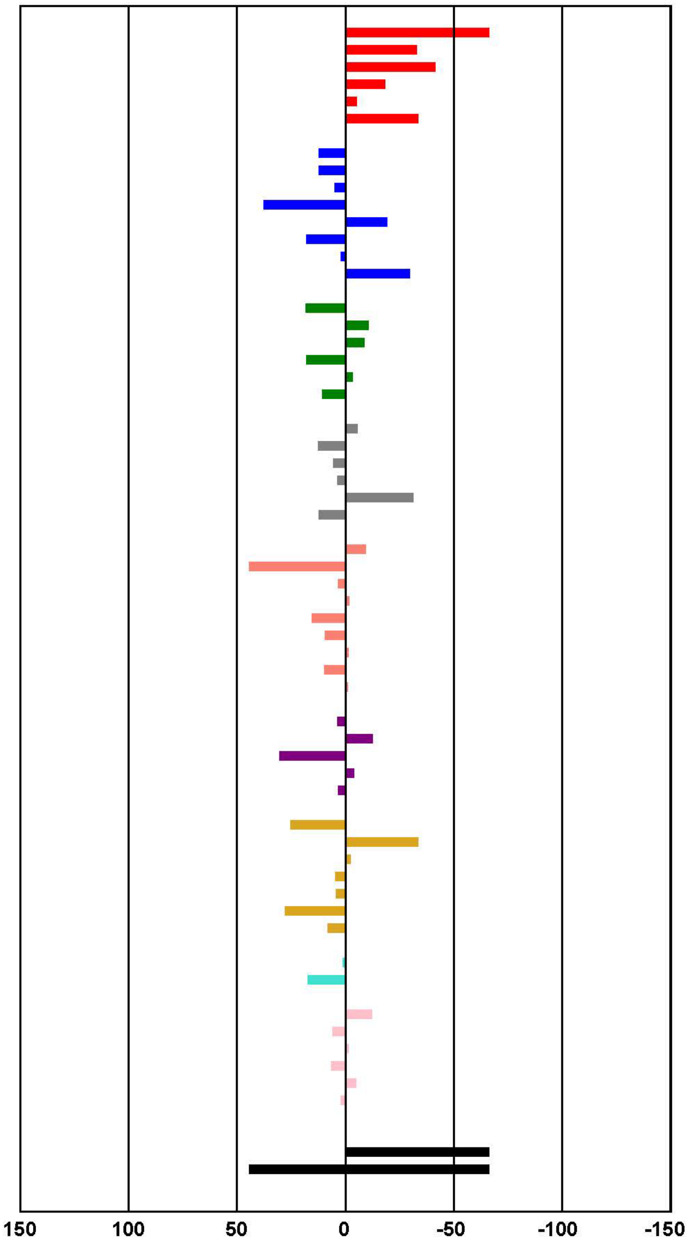

S4.2 Figure S8 NCI One dose data for Compound 5

Developmental Therapeutics Program One Dose Mean Graph	**NSC:** 815378 / 1	**Conc:** 1.00E-5 Molar	**Test Date:** May 20, 2019
	**Experiment ID:** 1905OS11		**Report Date:** Oct 04, 2022

Panel/Cell Line	Growth Percent
Leukemia	
CCRF-CEM	32.49
HL-60(TB)	50.82
K-562	58.36
MOLT-4	80.33
RPMI-8226	84.09
SR	64.44
Non-Small Cell Lung Cancer	
A549/ATCC	102.70
EKVX	96.18
HOP-62	82.62
HOP-92	132.37
NCI-H23	78.31
NCI-H322M	101.48
NCI-H460	97.02
NCI-H522	62.19
Colon Cancer	
HCC-2998	99.63
HCT-116	77.26
HCT-15	74.27
HT29	103.70
KM12	97.73
SW-620	97.74
CNS Cancer	
SF-268	90.68
SF-295	99.20
SF-539	103.26
SNB-19	93.83
SNB-75	75.60
U251	91.54
Melanoma	
LOX IMVI	76.02
MALME-3M	119.60
M14	109.58
MDA-MB-435	96.21
SK-MEL-2	100.87
SK-MEL-28	108.65
SK-MEL-5	89.76
UACC-257	92.95
UACC-62	87.23
Ovarian Cancer	
IGROV1	85.94
OVCAR-3	87.78
OVCAR-4	74.95
OVCAR-5	113.82
OVCAR-8	84.83
NCI/ADR-RES	90.96
Renal Cancer	
786-0	111.45
ACHN	58.50
CAKI-1	89.16
RXF 393	94.99
SN12C	87.97
TK-10	103.51
UO-31	87.92
Prostate Cancer	
PC-3	87.79
DU-145	110.33
Breast Cancer	
MCF7	75.15
MDA-MB-231/ATCC	71.49
HS 578T	98.10
BT-549	91.80
T-47D	65.13
MDA-MB-468	75.81
Mean	88.50
Delta	56.01
Range	99.88

Mean Growth Percent - Growth Percent

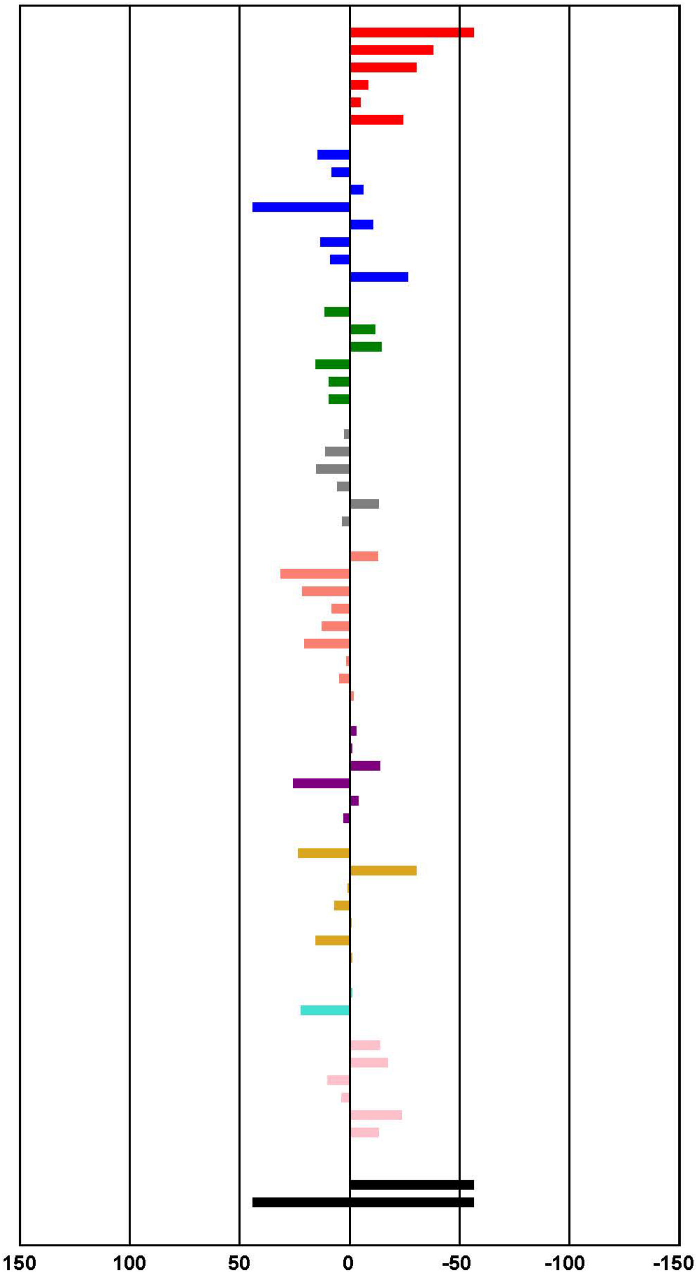

S4.3 Figure S9 NCI One dose data for Compound 6

Developmental Therapeutics Program One Dose Mean Graph	NSC: 815383 / 1	Conc: 1.00E-5 Molar	Test Date: May 20, 2019
	Experiment ID: 1905OS11		Report Date: Oct 04, 2022

Panel/Cell Line	Growth Percent
Leukemia	
CCRF-CEM	40.22
HL-60(TB)	54.03
K-562	19.99
MOLT-4	66.21
RPMI-8226	43.40
SR	64.58
Non-Small Cell Lung Cancer	
A549/ATCC	99.40
EKVX	102.90
HOP-62	124.50
HOP-92	123.39
NCI-H226	76.52
NCI-H23	94.94
NCI-H322M	100.80
NCI-H460	100.99
NCI-H522	-43.92
Colon Cancer	
HCC-2998	107.97
HCT-116	90.80
HCT-15	81.81
HT29	107.81
KM12	101.49
SW-620	84.70
CNS Cancer	
SF-268	90.83
SF-295	101.15
SF-539	95.93
SNB-19	109.98
SNB-75	82.95
U251	95.26
Melanoma	
LOX IMVI	88.53
MALME-3M	93.92
M14	103.44
MDA-MB-435	100.94
SK-MEL-2	110.09
SK-MEL-28	112.69
SK-MEL-5	91.38
UACC-257	95.48
UACC-62	99.65
Ovarian Cancer	
IGROV1	108.58
OVCAR-3	73.20
OVCAR-4	96.59
OVCAR-5	111.49
OVCAR-8	86.98
NCI/ADR-RES	97.13
Renal Cancer	
786-0	107.46
ACHN	9.92
CAKI-1	99.11
RXF 393	107.10
SN12C	100.40
TK-10	100.63
UO-31	93.92
Prostate Cancer	
PC-3	98.90
DU-145	114.28
Breast Cancer	
MCF7	81.06
MDA-MB-231/ATCC	104.90
HS 578T	105.13
BT-549	85.37
T-47D	40.00
MDA-MB-468	87.67
Mean	88.15
Delta	132.07
Range	168.42

Mean Growth Percent - Growth Percent

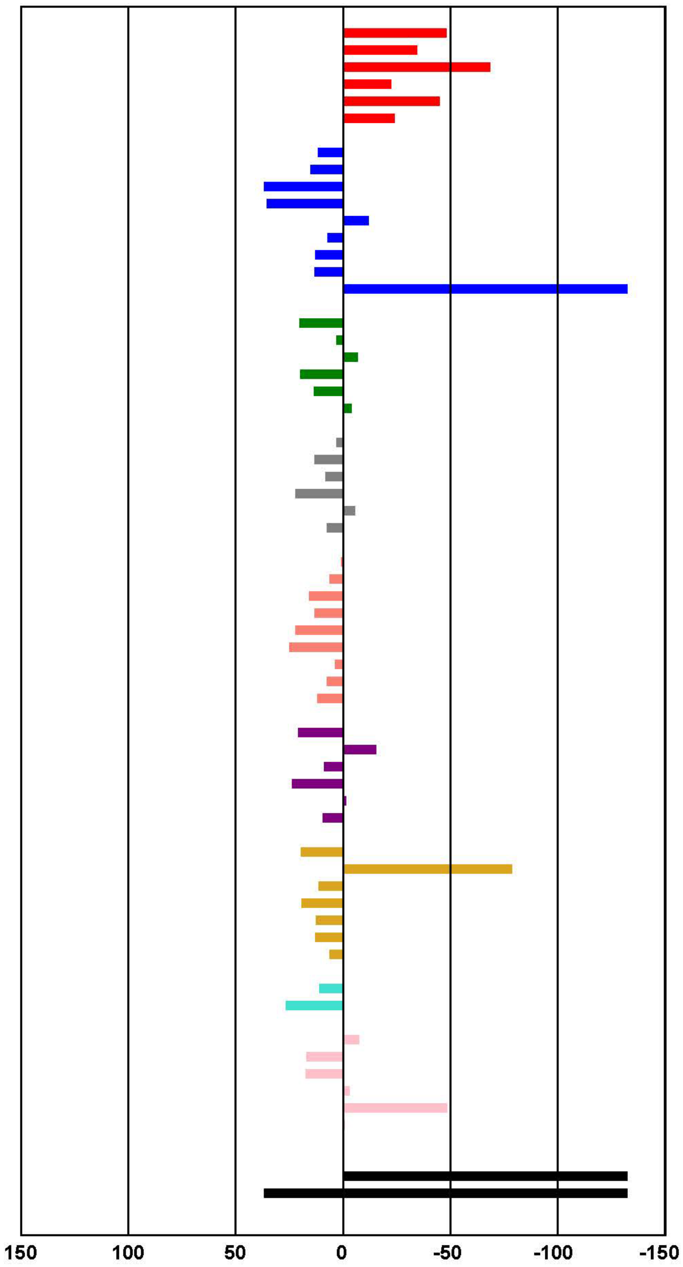

S4.4 Figure S10 NCI One dose data for Compound 7

Developmental Therapeutics Program One Dose Mean Graph	NSC: 815376 / 1	Conc: 1.00E-5 Molar	Test Date: May 20, 2019
	Experiment ID: 1905OS11		Report Date: Oct 04, 2022

Panel/Cell Line	Growth Percent
Leukemia	
CCRF-CEM	83.44
HL-60(TB)	58.78
K-562	88.04
MOLT-4	96.77
RPMI-8226	100.82
SR	79.87
Non-Small Cell Lung Cancer	
A549/ATCC	106.40
EKVX	103.81
HOP-62	109.32
HOP-92	136.09
NCI-H23	91.39
NCI-H322M	98.18
NCI-H460	99.10
NCI-H522	77.44
Colon Cancer	
HCC-2998	105.30
HCT-116	87.16
HCT-15	86.32
HT29	107.49
KM12	98.80
SW-620	102.34
CNS Cancer	
SF-268	87.98
SF-295	100.71
SF-539	102.03
SNB-19	96.67
SNB-75	83.71
U251	94.10
Melanoma	
LOX IMVI	84.99
MALME-3M	133.76
M14	94.27
MDA-MB-435	97.01
SK-MEL-2	107.30
SK-MEL-28	106.53
SK-MEL-5	96.58
UACC-257	93.65
UACC-62	107.32
Ovarian Cancer	
IGROV1	104.54
OVCAR-3	90.87
OVCAR-4	88.20
OVCAR-5	108.74
OVCAR-8	88.26
NCI/ADR-RES	94.47
Renal Cancer	
786-0	103.08
ACHN	78.37
CAKI-1	98.46
RXF 393	109.90
SN12C	98.53
TK-10	109.80
UO-31	98.74
Prostate Cancer	
PC-3	95.21
DU-145	108.80
Breast Cancer	
MCF7	79.86
MDA-MB-231/ATCC	116.06
HS 578T	108.12
BT-549	90.75
T-47D	87.40
MDA-MB-468	88.54
Mean	97.32
Delta	38.54
Range	77.31

Mean Growth Percent - Growth Percent

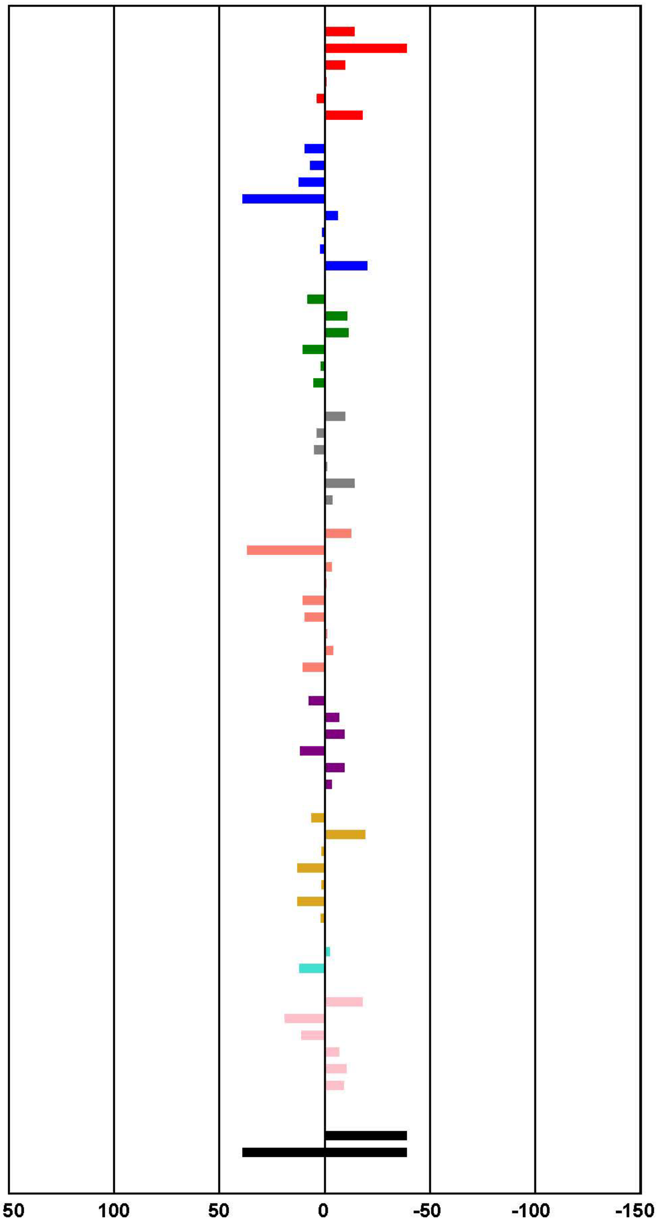

S4.5 Figure S11 NCI One dose data for Compound 12

Developmental Therapeutics Program One Dose Mean Graph	NSC: 815377 / 1	Conc: 1.00E-5 Molar	Test Date: May 20, 2019
	Experiment ID: 1905OS11		Report Date: Oct 04, 2022

Panel/Cell Line	Growth Percent
Leukemia	
CCRF-CEM	62.05
HL-60(TB)	41.05
K-562	77.05
MOLT-4	87.89
RPMI-8226	78.70
SR	92.15
Non-Small Cell Lung Cancer	
A549/ATCC	100.49
EKVX	104.07
HOP-62	116.30
HOP-92	107.60
NCI-H23	94.50
NCI-H322M	99.56
NCI-H460	99.07
NCI-H522	-27.00
Colon Cancer	
HCC-2998	100.53
HCT-116	87.32
HCT-15	84.66
HT29	97.63
KM12	102.47
SW-620	94.01
CNS Cancer	
SF-268	88.38
SF-295	102.59
SF-539	104.01
SNB-19	96.59
SNB-75	82.20
U251	94.53
Melanoma	
LOX IMVI	84.20
MALME-3M	115.21
M14	94.90
MDA-MB-435	100.60
SK-MEL-2	91.67
SK-MEL-28	116.30
SK-MEL-5	93.64
UACC-257	105.89
UACC-62	95.94
Ovarian Cancer	
IGROV1	103.88
OVCAR-3	84.17
OVCAR-4	94.49
OVCAR-5	114.49
OVCAR-8	92.27
NCI/ADR-RES	95.81
Renal Cancer	
786-0	104.31
ACHN	7.34
CAKI-1	97.53
RXF 393	103.81
SN12C	93.53
TK-10	103.30
UO-31	84.26
Prostate Cancer	
PC-3	95.04
DU-145	109.87
Breast Cancer	
MCF7	85.60
MDA-MB-231/ATCC	91.13
HS 578T	100.30
BT-549	86.52
T-47D	59.25
MDA-MB-468	91.32
Mean	90.45
Delta	117.45
Range	143.30

Mean Growth Percent - Growth Percent

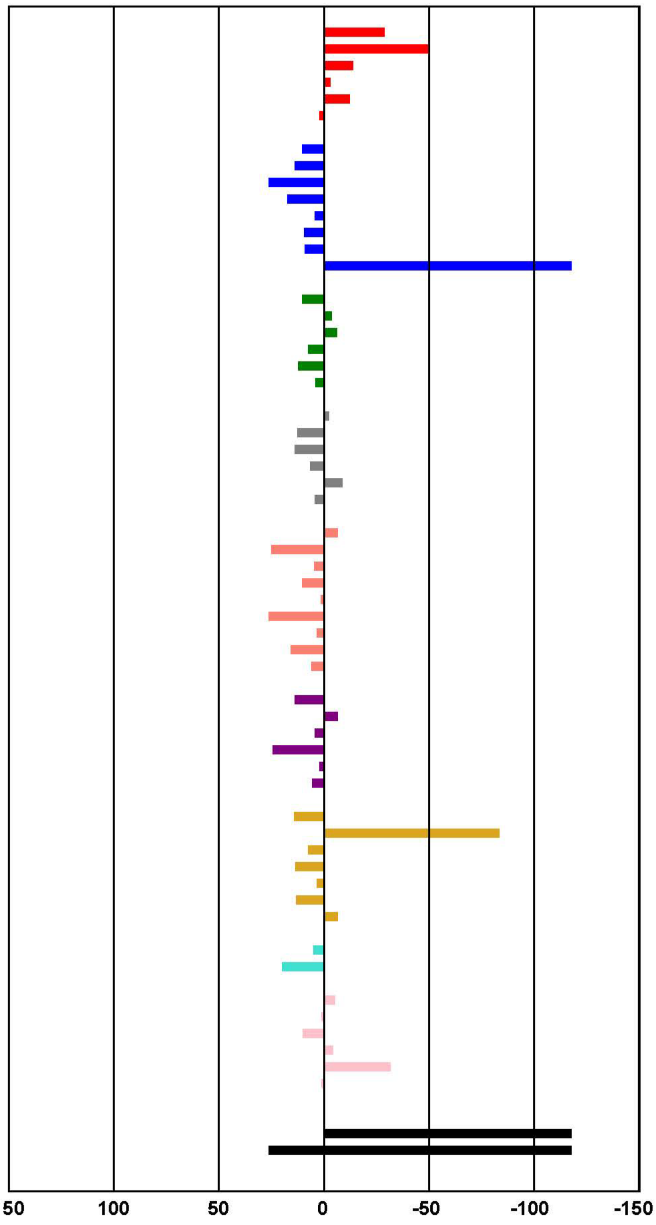

Supplement: Supplementary file 1 [file pharmaceuticals-18-00268-s001.zip › pharmaceuticals-3450654-supplementary.pdf]
